# Long‐Lived Charge‐Transfer State in Spiro Compact Electron Donor–Acceptor Dyads Based on Pyromellitimide‐Derived Rhodamine: Charge Transfer Dynamics and Electron Spin Polarization

**DOI:** 10.1002/anie.202203758

**Published:** 2022-04-21

**Authors:** Xi Chen, Andrey A. Sukhanov, Yuxin Yan, Damla Bese, Cagri Bese, Jianzhang Zhao, Violeta K. Voronkova, Antonio Barbon, Halime Gul Yaglioglu

**Affiliations:** ^1^ State Key Laboratory of Fine Chemicals School of Chemical Engineering Dalian University of Technology 2 Ling Gong Road Dalian 116024 P. R. China; ^2^ Zavoisky Physical-Technical Institute FRC Kazan Scientific Center of Russian Academy of Sciences Kazan 420029 Russia; ^3^ Dipartimento di Scienze Chimiche Università degli Studi di Padova 35131 Padova Italy; ^4^ Department of Engineering Physics Faculty of Engineering Ankara University 06100, Beşevler Ankara Turkey; ^5^ Department of Physics Engineering Hacettepe University 06800 Beytepe Ankara Turkey

**Keywords:** Charge Transfer, Charge-Separated State, Electron Transfer, Intersystem Crossing, Triplet State

## Abstract

We observed a long‐lived charge transfer (CT) state in a novel orthogonal compact electron donor–acceptor dyads, with closed form of rhodamine (Rho) as electron donor and pyromellitimide (PI),or thionated PI, as electron acceptor. The two parts in the dyads are connected via a spiro quaternary carbon atom, thus the torsion between the donor and acceptor is completely inhibited, which is beneficial to reduce the reorganization energy and to exploit the Marcus inverted region effect to prolong the CT state lifetime. Femtosecond transient absorption spectra show that the charge separation is rather fast, while nanosecond transient absorption spectra confirmed the formation of long‐lived CT state (2.6 μs). Time‐resolved electron paramagnetic resonance (TREPR) spectra determined the spin multiplicity of the long living state and assigned it to a ^3^CT state. Replacement of an oxygen atom in the PI part with a sulfur atom favoring classical intersystem crossing processes, causes a consistently shortening of the lifetime of the ^3^CT state (0.29 μs).

## Graphical abstract

A novel fully rigid spiro electron donor–acceptor dyad is prepared by linking the electron donor and acceptor via a spiro quaternary carbon atom, thus the torsion between the electron donor and acceptor is completely inhibited. The dyad shows a long‐lived ^3^CT state (2.6 μs) with a high energy level (ca. 2.61 eV), the electron spin multiplicity of the CT state was confirmed with time‐resolved EPR spectra.

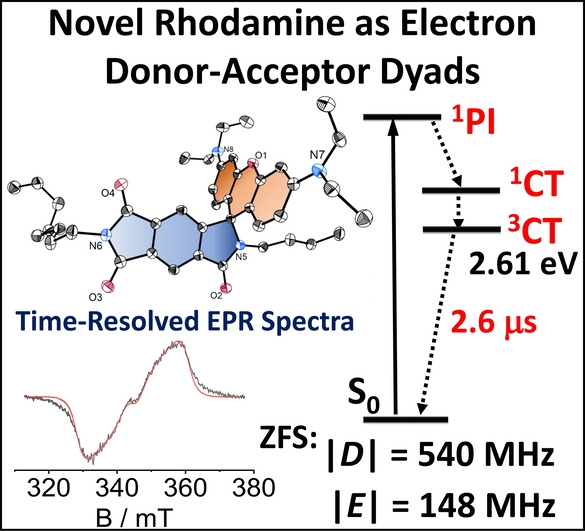

## Introduction

Accessing long‐lived charge‐transfer (CT) state is crucial for artificial photosynthesis, photocatalysis and photovoltaics, etc,[^1^, ^2^, ^3^, ^4^, ^5^] because the efficiency of the photophysical processes can be improved with long‐lived CT state. A few methods have been developed to prolong the CT state lifetimes.[^4^, ^5^] In conventional electron donor–acceptor dyads, the linkers between the electron donor and acceptor are long or multiple electron donors with different oxidation potentials were used,[^4^, ^6^] as a result, the weak electronic coupling of the final CT state will prolong the CT state lifetimes (Eq. (1)), but it is with the expense of CT state energy.^[4]^ Using Marcus inverted region effect is also able to prolong the CT state lifetimes,[^7^, ^8^] but the high CT state energy in this case push the absorption of the dyads to the blue/UV spectral range, which is clearly a disadvantage for the applications. Moreover, very often the Marcus inverted region effect is less significant than the theoretically predicted.[^9^, ^10^, ^11^, ^12^, ^13^, ^14^] The electron transfer (ET) can be described by Equation 1, according to Marcus theory:

(1) $$k_{\mathrm{ET}}={\left(\frac{{4\pi }^{3}}{h^{2}\lambda k_{B}T}\right)}^{\frac{1}{2}}H_{\mathrm{AB}}^{2}\exp\;\left[-\frac{{\left(\Delta G{{}^{\circ }}_{\mathrm{ET}}+\lambda \right)}^{2}}{4\lambda k_{B}T}\right]$$

where *λ* is the reorganization energy, Δ*G*°_ET_ is the free reaction energy and *H*_AB_ is the electronic coupling matrix element.

New approaches to prolong the CT state lifetimes are highly desired, especially those based on simple molecular structures. One factor that is not considered in empirical Equation (1),^[8]^ but may play a significant role in dictating the CT state lifetime, is the *electron spin control* in the charge separation (CS) and charge recombination (CR).[^4^, ^15^] The CS and CR processes are characterized by electron spin conservation. For instance, given the singlet excited state (locally excited state: ^1^LE) is the precursor of CT, the formation of ^1^CT state is bound to prevail than formation of the ^3^CT state.^[4]^ On the other hand, the CR of ^1^CT→S_0_ (ground state: S_0_) is an internal conversion (IC), which is intrinsically faster than the ^3^CT→S_0_, an electron spin forbidden intersystem crossing (ISC) process. Thus, ^3^CT state should be intrinsically longer‐lived than the ^1^CT state.[^4^, ^5^]

Formation of ^3^CT state requires ^3^LE state as precursor of CT. As such, transition metal complexes with ultrafast ISC (≈fs) to form ^3^MLCT state (MLCT: metal‐to‐ligand charge transfer),[^14^, ^16^, ^17^, ^18^, ^19^] or organic chromophore with intrinsic fast ISC (e.g. anthraquinone),^[20]^ were used to achieve the electron spin control effect to access the long‐lived ^3^CT state. However, drawbacks exist for these methods, e.g. using of the chromophores with intrinsic ISC capability limits the availability of the suitable electron donor and acceptors to construct the dyads. Moreover, in some of the previously reported molecular systems, very often spin correlated radical pairs (SCRP) are formed. Without sufficient electron spin‐spin interaction, no stable ^3^CT state will be formed, even with ^3^LE precursor of CT.[^19^, ^21^] Moreover, normally the electron spin multiplicity of the CT state was not clarified with pulsed laser excited time‐resolved electron paramagnetic resonance (TREPR) spectroscopy.^[4]^

Recently we reported a new approach to attain the ^3^CT state without invoking of a chromophore showing intrinsic ISC ability.^[22]^ The approach is based on the spin‐orbit charge transfer ISC (SOCT‐ISC) in a compact electron donor‐acceptor dyad, in which the donor π‐conjugation plane adopts *orthogonal* geometry against the electron acceptor π‐conjugation plane. Under this circumstance, the CR between the donor and acceptor is accompanied with molecular orbital angular momentum change, which offsets the electron spin angular momentum of ISC, thus efficient CR‐induced ISC is achieved, i.e. SOCT‐ISC occurs.[^23^, ^24^, ^25^, ^26^] With this strategy, we attained a ^3^CT state with lifetime of 0.93 μs in fluid solution at room temperature with a rhodamine (Rho)‐naphthalimide (NI) *compact* dyad.^[22]^ The promising aspects of this strategy are that the intrinsic ISC of the chromophores are not mandatory to achieve the electron spin control, and the feasible synthesis, and the high CT state energy (no cascade ET processes are required). However, the molecular geometry of the reported dyads is not *fully rigid*, torsion between the donor and acceptor moieties is still possible, which is detrimental to attain efficient SOCT‐ISC. Moreover, rhodamine derivatives with large π‐conjugation framework at the 9‐position of the xanthene moiety, instead of the very often used phenyl moiety, were rarely reported. Although a spiro electron donor‐acceptor dyad was reported recently,^[27]^ yet the electron donating or accepting feature is not optimized for ET, and ^3^LE state, not ^3^CT state, was observed for that dyad.

**Scheme 1:**
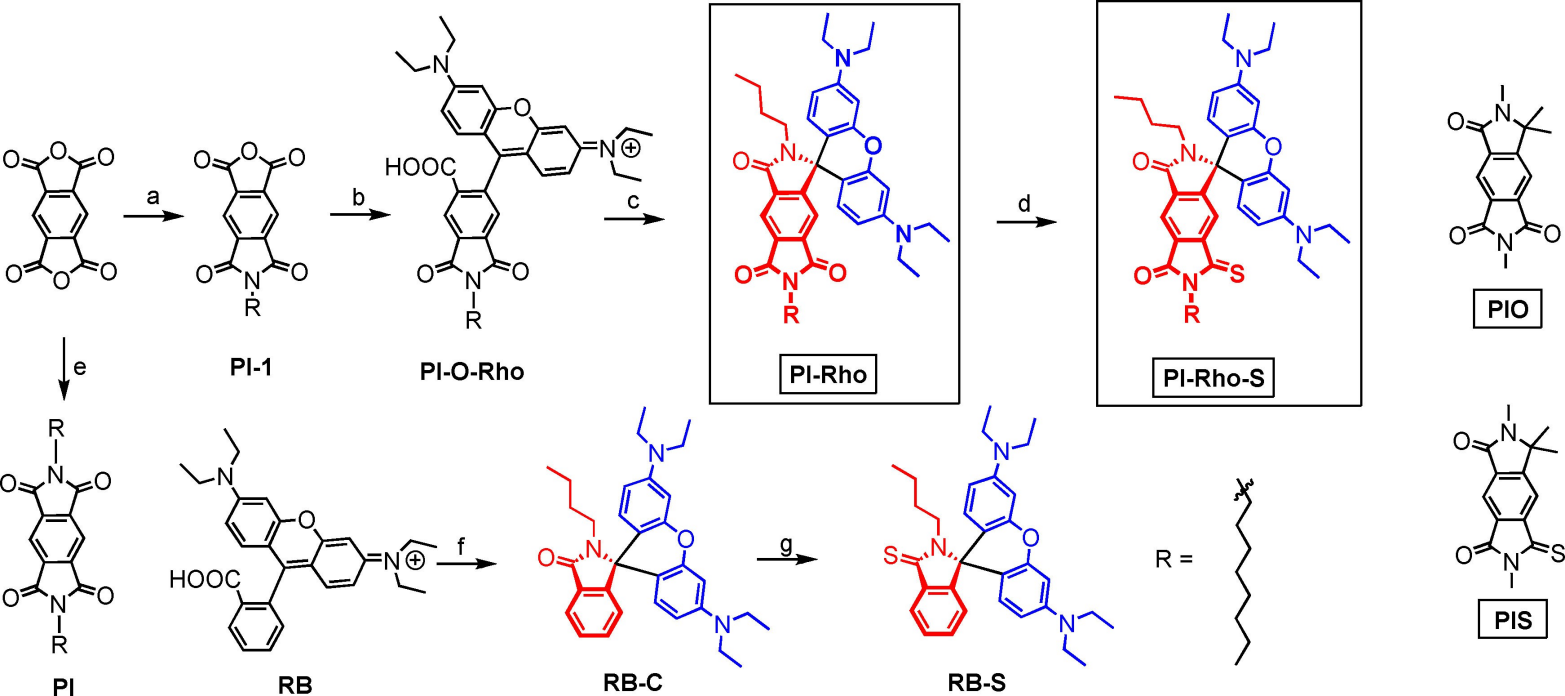
Synthesis of spiro electron donor–acceptor dyads **PI‐Rho** and **PI‐Rho‐S**. The two reference molecular structures **PIO** and **PIS** are also presented (note these compounds were not synthesized). a) *n*‐Octylamine, DMF, 80 °C, 10 h, under N_2_, yield: 30 %; b) 3‐diethylaminophenol, H_2_SO_4_, 180 °C, 4 h, under N_2_, yield: 35 %; c) POCl_3_, 1,2‐dichloroethane, 80 °C, 6 h; *n*‐butylamine, acetonitrile (ACN), Et_3_N, reflux, 25 h; yield: 40 %; d) Lawesson's Reagent, *p*‐xylene, 150 °C, 3 h, under N_2_, yield: 20 %; e) Similar to step (a), yield: 81 %; f) Similar to step (c), yield: 90 %; g) Similar to step (d), yield: 95 %.

In order to address these challenges and to prepare an electron donor‐acceptor dyad, with fully fixed geometry, herein we propose a general molecular structural motif of using pyromellitimide (PI) and the rhodamine to prepare a compact dyad (Scheme 1). The rhodamine derivative (**PI‐Rho**) contains two units, the lactam form of the rhodamine unit (electron donor) and the substituted PI unit (electron acceptor), and the two units are connected by a *spiro* quaternary carbon atom, therefore the geometry is completely fixed and no torsion is possible, this is a novel molecular structure for electron donor‐acceptor dyad, as well as for rhodamine chromophore. The photophysical properties of the dyads were studied with steady state and time‐resolved absorption and emission spectroscopic methods, as well as TREPR spectra. Long‐lived ^3^CT state was observed in fluid solution at room temperature.

## Results and Discussion

Rhodamine is one of the mostly investigated chromophores, due to its strong absorption of visible light, and feasible derivatization.^[28]^ However, most of the derivatization is to attach different moieties on the amide *N* position, derivatization on the phenyl moiety to extend the π‐conjugation framework is rare.[^29^, ^30^]

We envisaged that PI, an electron acceptor, can be used to prepare rhodamine derivatives (Scheme 1). The electron donor moiety (xanthene amine) and the PI are connected with a spiro quaternary carbon atom in **PI‐Rho**. The advantages of this molecular design are as followings: 1) PI moiety is a strong electron acceptor (*E*_Red_=−1.37 V, Fc/Fc^+^), and the lactam rhodamine part is an electron donor (*E*_Ox_=+0.54 V, Fc/Fc^+^);[^31^, ^32^] 2) the electron acceptor (PI) is connected with the electron donor via *spiro* quaternary carbon, thus the orthogonal geometry is completely fixed, the SOCT‐ISC efficiency can be maximized; the ^3^CT state may be stabilized as well; 3) native PI moiety is with high T_1_ state energy (*E*_T1_=ca. 2.45 eV),^[31]^ thus a low‐lying ^3^CT state maybe formed; 4) the electron donor–acceptor are separated by two σ‐bonds to reduce the electronic coupling between them, which is also beneficial for prolongation of the CT state lifetimes.[^4^, ^5^] In order to enhance the electron accepting ability,^[33]^ and to move the absorption wavelength to lower energy range,[^34^, ^35^] thionation of the carbonyl group in PI moiety was carried out and **PI‐Rho‐S** was prepared (Scheme 1).

The synthesis of the dyads is based on routine derivatization chemistry of rhodamine,^[36]^ with pyromellitic monoanhydride (**PI‐1**) as the starting material, the lactam form **PI‐Rho** was obtained. This is a novel structure for electron donor‐acceptor dyad and rhodamine derivatives. We used 2D ^1^H detected heteronuclear multiple bond correlation (HMBC) NMR data to elucidate the molecular structure (Figure S14). Reference compounds **RB‐C** and **RB‐S** were also prepared for comparison in study of the photophysical properties. The molecular structures of the compounds were verified with single‐crystal X‐ray diffraction and ^1^H NMR, ^13^C NMR spectra and HR MS, etc.

**Figure 1:**
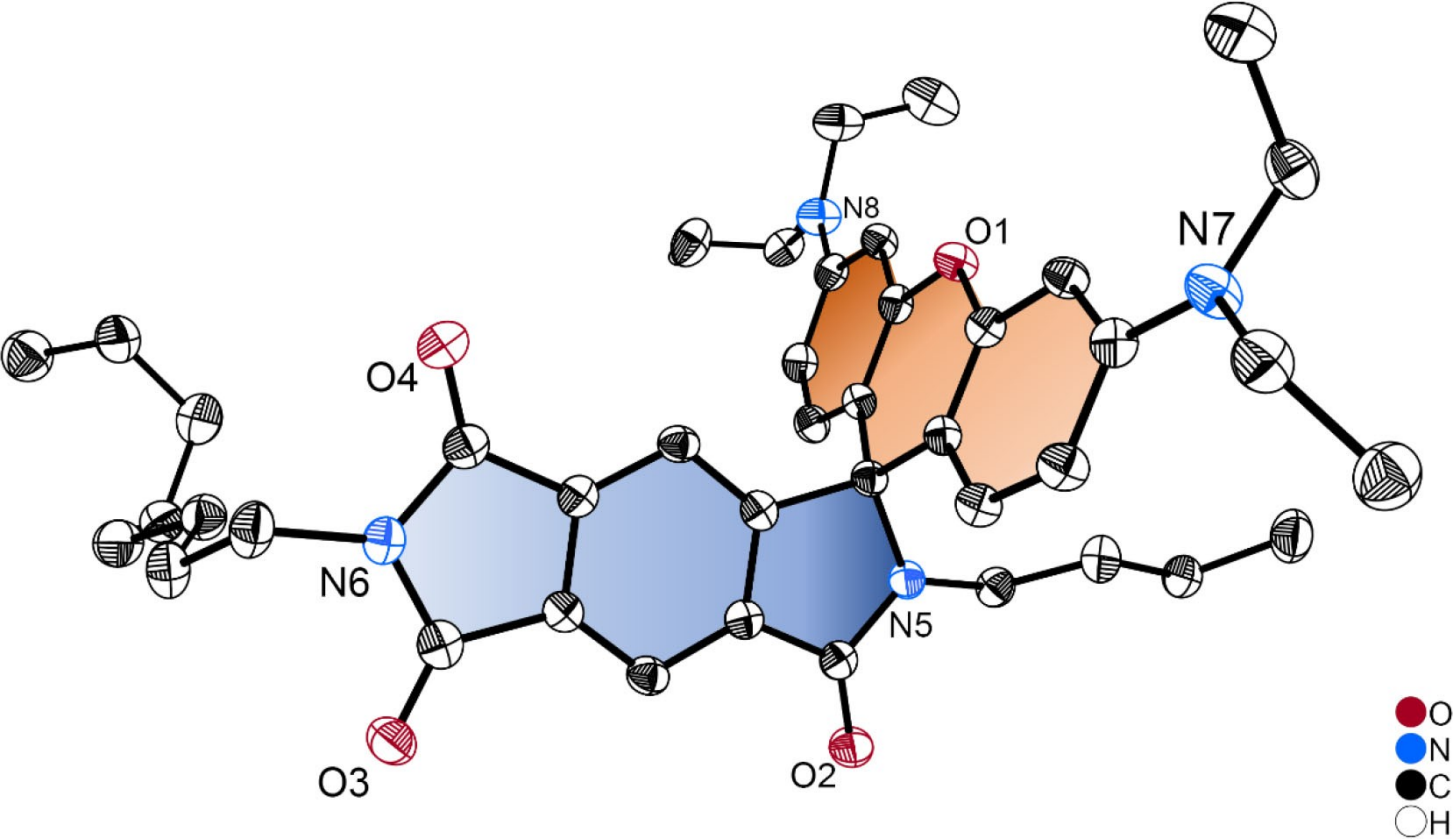
ORTEP view of the molecular structure of **PI‐Rho** determined by single‐crystal X‐ray diffraction. Hydrogen atoms are omitted for clarity. Thermal ellipsoids are set at 50 % probability. CCDC number: 2075565 contains detailed information.^[37]^

The single crystal of **PI‐Rho** was obtained by slow evaporation of the solution in dichloromethane (DCM)/HEX. The molecular structure of **PI‐Rho** was verified by single‐crystal X‐ray diffraction (Figure 1). The xanthene π‐conjugation framework is a coplanar structure, the PI moiety is also in a planar geometry. The two parts are connected with a spiro quaternary carbon atom and the π‐planes of the two units adopt a dihedral angle of 87° (Figure S20). We used DFT computation to optimize the S_0_ geometry of the compounds, similar results were obtained. For **PI‐Rho‐S**, the dihedral angle between the planes of the xanthene and the PI moieties is 90° (see later section for details).

**Figure 2:**
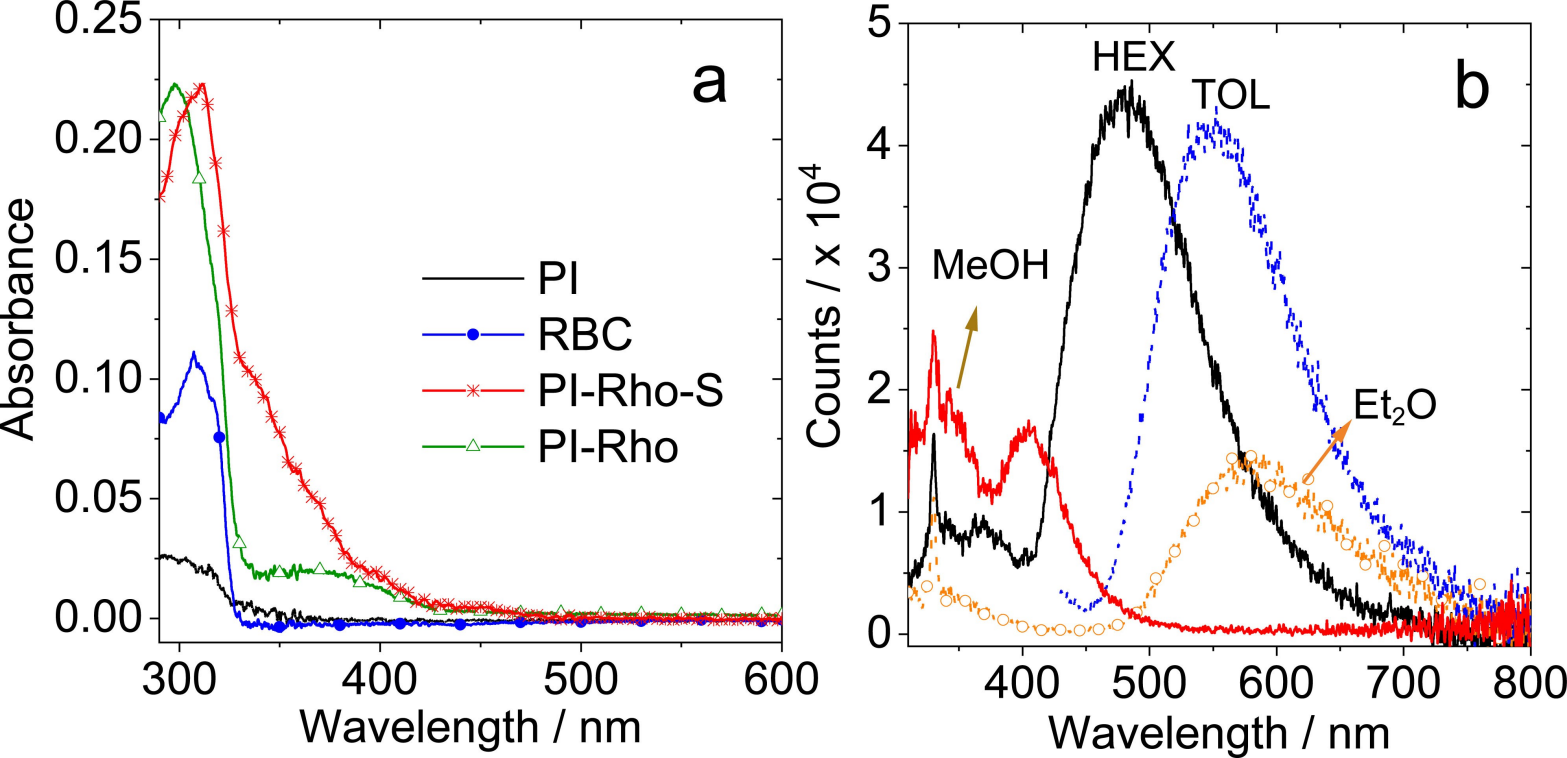
a) UV/Vis absorption spectra of **PI**, **RB‐C**, **PI‐Rho** and **PI‐Rho‐S** in *n*‐hexane (HEX). b) Fluorescence emission spectra of **PI‐Rho** (optically matched solutions were used, *A*=0.106, *λ*_ex_=300 nm. *c*≈1.0×10^−5^ M), 20 °C. The solvents used are HEX, toluene (TOL), diethyl ether (Et_2_O), and methanol (MeOH). The peaks at 331 nm in (b) represent Raman scattering of the solvent.

The UV/Vis absorption spectra of the compounds were studied (Figure 2). The closed form of **PI‐Rho** doesn't show any absorption in the visible spectral region. A strong absorption band at 297 nm was observed, which is attributed to the Pl moiety. Moreover, the weaker, broad absorption band in the range of 330–450 nm is assigned to a CT absorption of the transition of S_0_→^1^CT. Based on the CT absorption band, the *H*_AB_ of the S_0_ and Franck–Condon ^1^CT state was calculated as 1397 cm^−1^ in HEX (Table S2).^[4]^ The thionated analogue **PI‐Rho‐S** shows a similar CT absorption band, with *H*_AB_=1484 cm^−1^. This is an interesting result, because the electron donor‐acceptor is separated by two σ‐bonds,^[38]^ but still significant electronic coupling at S_0_ state was observed. In the presence of acid, a strong absorption band at 565 nm was observed for **PI‐Rho**. The closure‐opening transformation of the two newly prepared rhodamine compound in the presence of base and acid, respectively, is reversible (Figure S24).

The weak fluorescence emission in the range of 300–400 nm of **PI‐Rho** is attributed to the PI moiety (Figure 2b), supported by the reference **PI** (Figure S25). Moreover, a stronger, broad emission band centered at 481 nm was observed in HEX, and this band is red‐shifted in polar solvents, for instance it is red‐shifted to 581 nm in Et_2_O. This emission band is attributed to a CT fluorescence (^1^CT→S_0_ transition). Based on the CT emission band, the *H*_AB_ of the relaxed ^1^CT state and S_0_ was calculated as 1629 cm^−1^ in HEX. Negligible fluorescence was observed for **PI‐Rho‐S**, which is supposed due to the enhanced ISC upon thionation.[^34^, ^35^] In the presence of acid, a strong fluorescence emission band centered at 585 nm was observed. This is the featured fluorescence emission of rhodamine compounds.^[30]^ Similar results were observed for **PI‐Rho‐S** (Figure S25). The photophysical parameters are summarized in Table 1.

**Table 1:**
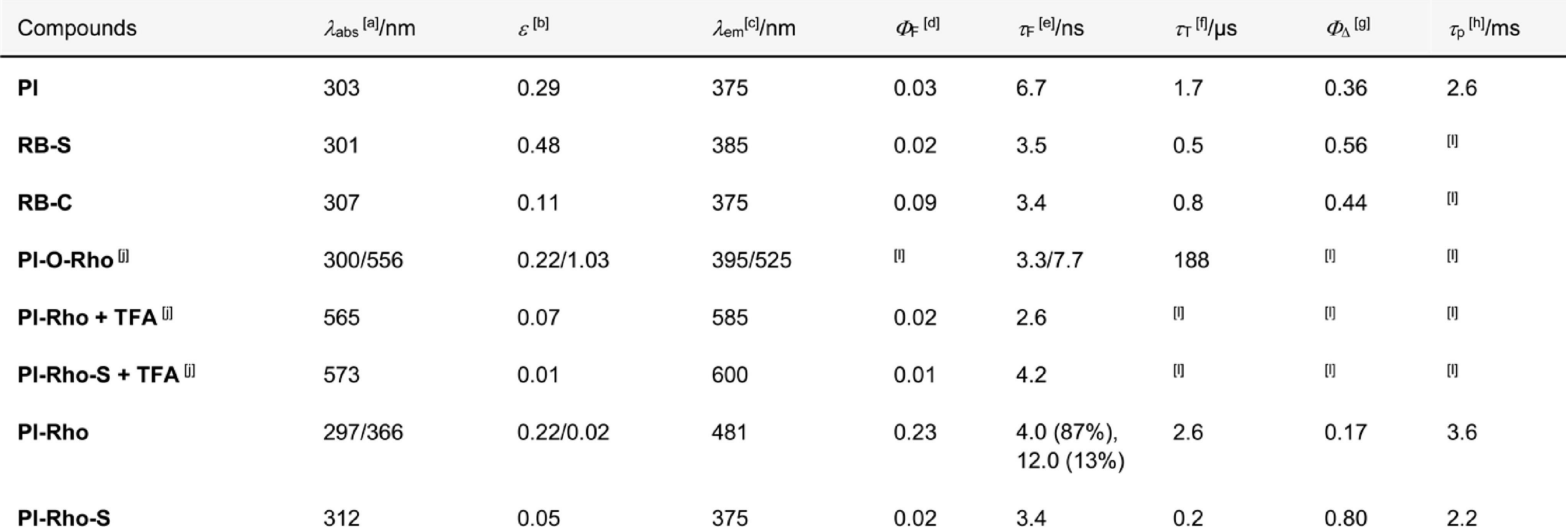
Photophysical properties of the compounds.

[a] In HEX (*c*=1.0×10^−5^ M, 25 °C). [b] Molar absorption coefficient (*ϵ*=10^5^ M^−1^ cm^−1^). [c] Fluorescence emission in HEX, *A*=0.106, *λ*_ex_=300 nm. [d] Absolute fluorescence quantum yield, *λ*_ex_=300 nm. [e] Fluorescence lifetimes, *c*=1.0×10^−5^ M, 25 °C, *λ*_ex_=340 nm. [f] Triplet lifetime and ^3^CT lifetime, in TOL, under N_2_ atmosphere. [g] Singlet oxygen quantum yield in TOL measured with Ru(bpy)_3_[PF_6_]_2_ as standard (*Φ*_Δ_=0.57 in DCM). [h] Phosphorescence lifetimes, at 77 K in 2‐methyltetrahydrofuran (2‐MeTHF), *λ*_ex_=340 nm (determined with microsecond flash lamp). *λ*_dec_=560 nm. [i] Not applicable. [j] In MeOH.

The native PI gives moderate singlet oxygen (^1^O_2_) quantum yield (*Φ*_Δ_=36 %. Table 2),^[31]^ **PI‐Rho‐S** gives higher *Φ*_Δ_ (*Φ*_Δ_=80 %) than **PI‐Rho** (*Φ*_Δ_=17 %). **PI‐Rho** shows higher *Φ*_Δ_ values in HEX and TOL, but negligible *Φ*_Δ_ in DCM and ACN. Similar trend was observed for **PI‐Rho‐S**. Considering the high T_1_ state energy of PI (2.45 eV),^[31]^ the formation of low‐lying CT state is possible, which is probably responsible for the moderate ^1^O_2_ photosensitizing ability, especially in polar solvents.

**Table 2:**
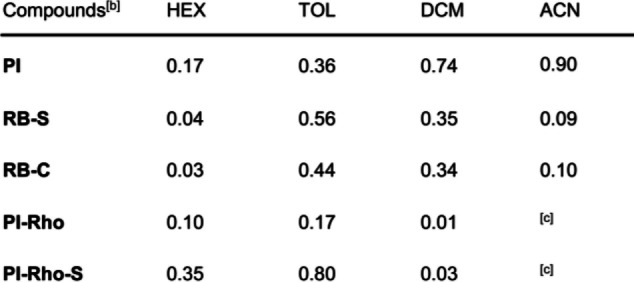
Singlet oxygen quantum yields (*Φ*_Δ_) of the compounds in different solvents.^[a]^

[a] The *E*_T_ (30) values of the solvents are HEX (31.0), TOL (33.9), DCM (40.7) and ACN (45.6), in kcal mol^−1^. [b] *Φ*_Δ_ with Ru(bpy)_3_[PF_6_]_2_ as standard (*Φ*_Δ_=0.57 in DCM). [c] Not observed.

**Figure 3:**
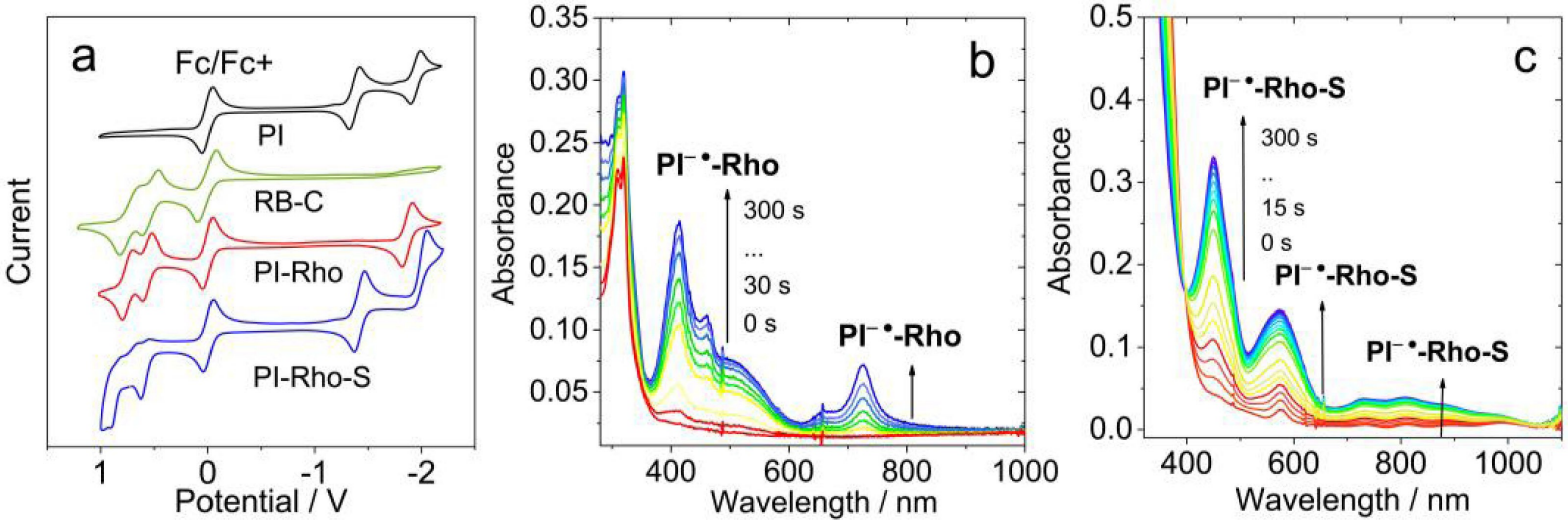
a) Cyclic voltammograms of **PI**, **PI‐Rho**, **PI‐Rho‐S** and **RB‐C**. Condition: in deaerated DCM containing 0.10 M Bu_4_N[PF_6_] as supporting electrolyte, Ag/AgNO_3_ as reference electrode. Ferrocene (Fc/Fc^+^) was used as internal reference. Scan rates: 50 mV s^−1^. *c*=1.0×10^−5^ M, 20 °C. Spectroelectrochemistry spectra of b) **PI‐Rho** upon potential of −1.90 V(Ag/AgNO_3_) applied and c) **PI‐Rho‐S** upon reduction potential of −1.27 V (Ag/AgNO_3_) applied, in deaerated DCM containing 0.10 M Bu_4_N[PF_6_] as supporting electrolyte, Ag/AgNO_3_ as reference electrode. *c*=4.0×10^−5^ M, 20 °C.

The redox potentials of compounds were studied with cyclic voltammogram (Figure 3a). **PI** shows two reversible reduction waves at −1.37 V and −1.95 V (vs. Fc/Fc^+^), respectively. For **PI‐Rho**, one reversible reduction wave at −1.86 V was observed. This reduction potential is more negative than the native PI (−1.37 V). Reversible oxidation waves at +0.56 and +0.75 V were observed, which are similar to that of native rhodamine (**RB‐C**), thus these oxidation waves are assigned to the xanthene moiety. **PI‐Rho‐S** shows a reversible reduction wave at −1.42 V (vs. Fc/Fc^+^) as well as an irreversible reduction wave at −2.05 V (vs. Fc/Fc^+^). This result indicates that thionated PI moiety is a stronger electron acceptor as compared to the PI unit in **PI‐Rho** (the reduction wave is at −1.86 V, vs. Fc/Fc^+^).^[33]^ The first reduction potential of **PI‐Rho‐S** is similar to that of native **PI**. This anodically shifted reduction potential of PI unit in **PI‐Rho‐S** is also beneficial for attaining low‐lying CT states.^[4]^

We studied the absorption of the radical anion of the dyads by spectroelectrochemistry (Figure 3b and c). With a potential of −1.90 V (vs. Ag/AgNO_3_) applied, the **PI‐Rho** solution shows new absorption bands centered at 413 nm, 460 nm, 489 and 724 nm, which are attributed to PI^−.^. Interestingly, the substituted PI^−.^ (there are only three carbonyl groups) shows absorption bands similar to the native PI radical anion (Figure S29).^[32]^ For **PI‐Rho‐S**, absorption bands centered at 450 nm and 574 nm were observed (Figure 3c), as well as weak absorption band in the range of 700–1000 nm, which are different from **PI‐Rho**. The radical cation absorption of the dyads was observed by imposing positive potential on the compounds (Figure S29). The radical cation absorption of **PI‐Rho**, **PI‐Rho‐S** and **RB‐C** all show sharp absorption band centered at 575 nm (Figure S29).

Based on the redox potentials, the Δ*G*_CS_ of the photo‐induced ET were calculated (Table 3). The results show that the CT is thermodynamically allowed even in HEX. For instance, the Δ*G*_CS_ is −1.18 eV in HEX, −1.30 eV in TOL and −1.75 eV in ACN. Considering the high T_1_ state energy of the PI moiety (2.45 eV),^[31]^ we anticipate formation of CT states for **PI‐Rho** and **PI‐Rho‐S**, especially in polar solvents.

**Table 3:**
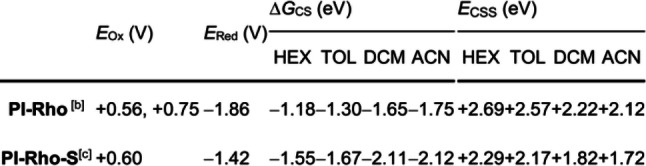
Redox potentials, driving forces of CS process (Δ*G*_CS_) and the energy of the CT states of the compounds in different solvents.^[a]^

[a] Cyclic voltammetry in N_2_‐saturated DCM containing a 0.10 M Bu_4_NPF_6_ supporting electrolyte; Pt electrode was used as the counter electrode; the working electrode is glassy carbon electrode; Ag/AgNO_3_ couple is the reference electrode. *E*_00_ is the energy level approximated with the crossing point of UV/Vis absorption and fluorescence emission spectra after normalization. [b] *E*_00_ is the crossing point of the normalized UV/Vis absorption and fluorescence spectra of compound. *E*_00_=3.88 eV. [c] *E*_00_=3.84 eV. The value was obtained by setting the oxidation potential of Fc^+^/Fc as 0 V.

**Figure 4:**
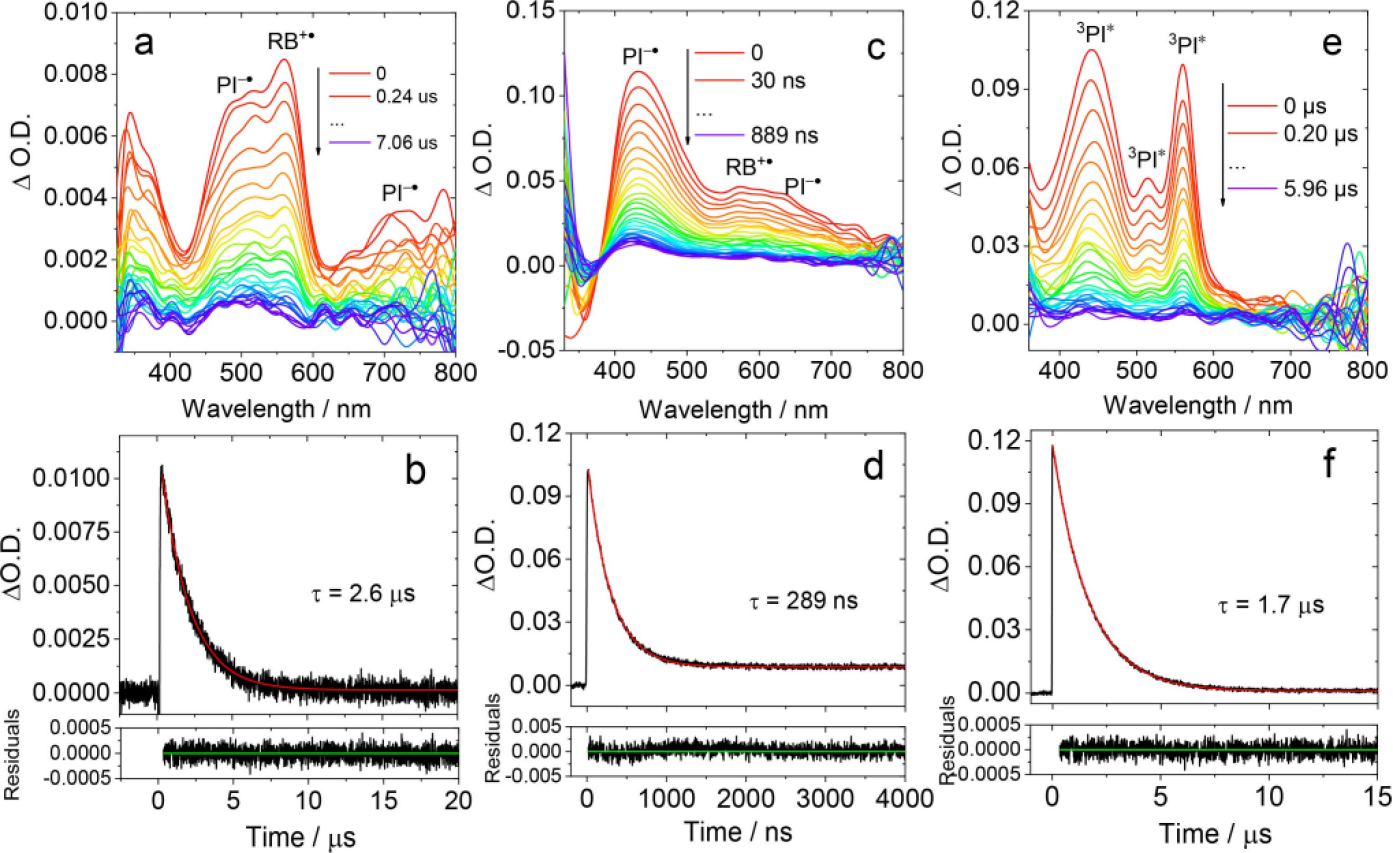
Nanosecond transient absorption spectra of a) **PI‐Rho**, c) **PI‐Rho‐S** in deaerated TOL and e) **PI** in deaerated DCM. The corresponding decay traces are b) **PI‐Rho** at 560 nm, d) **PI‐Rho‐S** at 430 nm and f) **PI** at 430 nm. *λ*_ex_=355 nm, *c*=1.0×10^−4^ M, 20 °C.

In order to study the formation of the CT state, nanosecond transient absorption (ns‐TA) spectra were studied (Figure 4). For the reference compound **PI**, excited state absorption (ESA) bands centered at 442, 514 and 559 nm were observed, and the three bands decay with the same kinetics, indicating they are from the same species (Figure 4e). The triplet state lifetime was determined as 1.7 μs. The lifetime was shortened to 823 ns in aerated solution (Figure S31), confirming the triplet spin multiplicity of the transient species.[^5^, ^39^]

For **PI‐Rho**, positive absorption bands centered at 489 nm, 559 nm and 724 nm were observed (Figure 4a), the 559 nm is attributed to the xanthene radical cation (RB^+.^),^[22]^ and the 489 nm and 724 nm are attributed to the PI radical anion (PI^−.^, Figure 3b).[^32^, ^40^] Thus, the transient species observed for **PI‐Rho** upon nanosecond laser excitation in deaerated TOL is a CT state. The lifetime of the CT state was determined as 2.6 μs by monitoring the decay trace at 560 nm (Figure 4b). In aerated solution, the CT state lifetime is shortened to 117 ns (Figure S31). It is known that the ^3^CT state can be quenched by O_2_.^[5]^ Assignment of the transient species as ^3^CT state, not ^1^CT state, is also supported by the CT fluorescence lifetime, which is due to ^1^CT state (Table 1). To the best of our knowledge, observation of long‐lived CT state in compact electron donor‐acceptor dyads is rare. Previously with some compact dyads, CT states were observed with lifetimes of 0.1–3.35 μs.[^4^, ^12^, ^14^, ^15^, ^25^] For **PI‐Rho**, same ns‐TA spectrum was observed in HEX, and the CT state lifetime was determined as 867 ns (Figure S32). The CT state lifetime of **PI‐Rho** (2.6 μs) is longer than the recently reported **NI‐Rho** dyad (0.94 μs).^[22]^

In polar solvent ACN, no ^3^LE state or CT state were observed, due to the fast CR, confirmed by the femtosecond transient absorption spectra. For **PI‐Rho‐S** (Figure 4c), positive absorption bands centered at 430 nm, 574 nm and 637 nm were observed, the bands centered at 430 nm and 637 nm are attributed to the PI^−.^ (Figure 3c and Figure S45). The positive absorption band centered at 574 nm is attributed to the Rho^+.^ (Figure S29). Therefore, the transient species observed for **PI‐Rho‐S** upon pulsed laser excitation in TOL is a CT state. The lifetime of the CT state was determined as 289 ns by monitoring the decay trace at 430 nm, which is shorter than that of **PI‐Rho**, probably because of the accelerated ISC due to the thionation of carbonyl group.[^34^, ^35^] In aerated solution, the CT lifetime was reduced to 165 ns (Figure S31). In ACN, similar results were obtained (Figure S32). Similar to that in TOL, triplet states absorption were also observed for **PI‐Rho‐S** in HEX and ACN, and the decay curves were 228 ns and 68 ns, respectively (Figure S32).

In order to unambiguously confirm that the transient species observed for **PI‐Rho** and **PI‐Rho‐S** with ns‐TA spectra are CT states, we used 5,10‐dimethyldihydrophenazine (PZ) as electron donor (*E*_Ox_=+0.5 V, vs. Fc/Fc^+^) and 7,7,8,8‐tetracyano‐quinodimethane (TCNQ) as electron acceptor (*E*_Red_=−0.26 V, vs. Fc/Fc^+^),^[41]^ to quench the radical cation and radical anion in **PI‐Rho** and **PI‐Rho‐S**, respectively. Diffusion‐controlled intermolecular ET between PZ (or TCNQ) and the dyads were observed. In the presence of PZ, the radical cations of the dyads were quenched, and the PZ^+.^ was observed,^[42]^ which shows absorption band centered at 535 nm and 645 nm (Figure S34 and S35). In the presence of TCNQ, the radical anions of the dyads were quenched, and the absorption band of TCNQ^−.^ centered at 435 nm and 666 nm was observed (Figure S34 and S35).^[43]^

**Figure 5:**
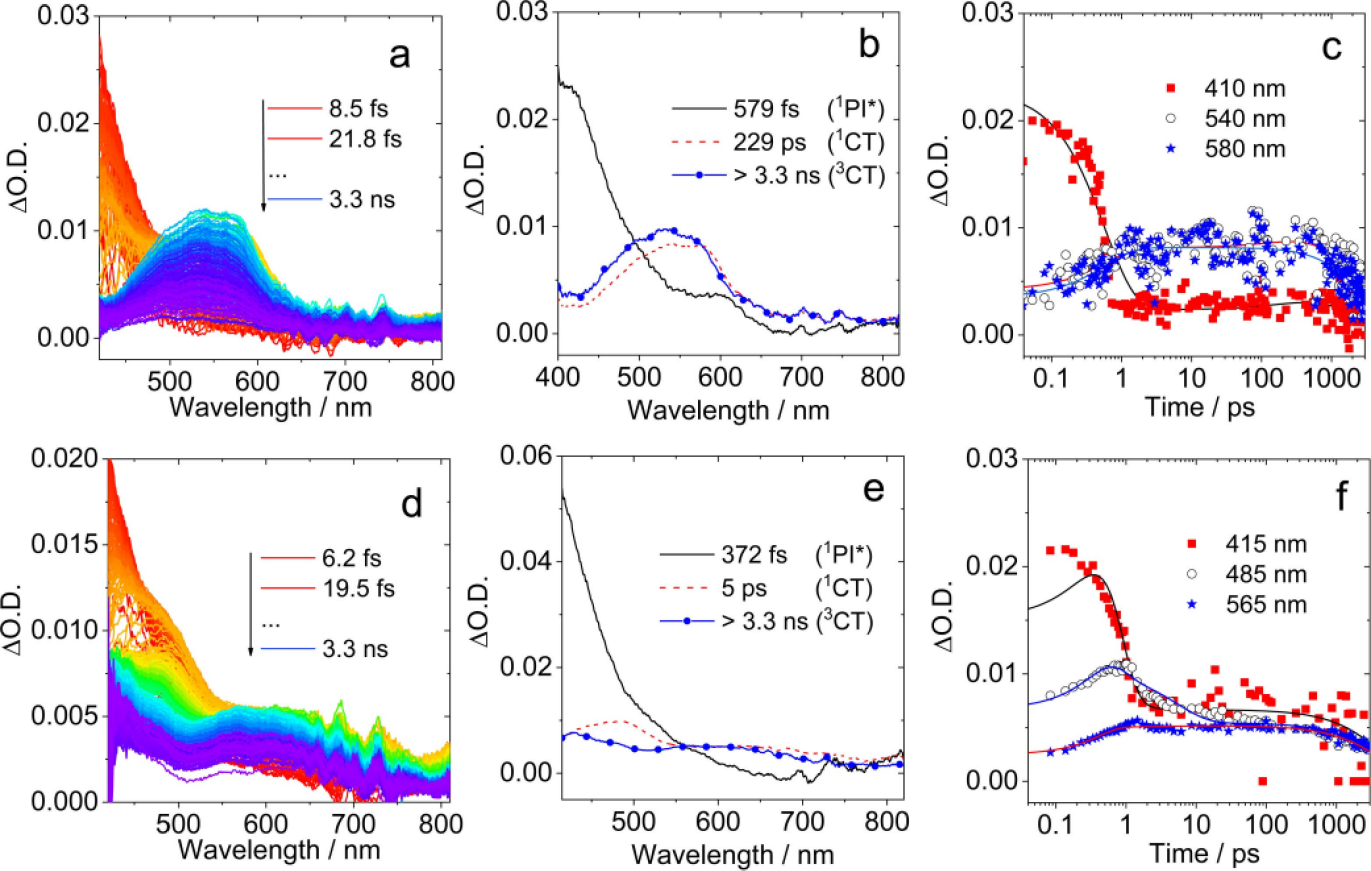
Femtosecond transient absorption spectra of a) **PI‐Rho**, color code goes from red to blue covering the time interval from 8.5 fs to 3.3 ns and d) **PI‐Rho‐S**, color code goes from red to blue covering the time interval from 6.2 fs to 3.3 ns. SADS of b) **PI‐Rho** and e) **PI‐Rho‐S** obtained from global analysis. Decay kinetics of c) **PI‐Rho** (at 410 nm, 540 nm and 580 nm) and f) **PI‐Rho‐S** (at 415 nm, 485 nm and 565 nm). In deaerated TOL, *λ*_ex_=330 nm, *c*=1.0×10^−3^ M, 20 °C.

Femtosecond transient absorption (fs TA) spectroscopy of the compounds was studied (Figure 5). The excitation was performed at 330 nm to promote the S_0_ state to the ^1^LE state of PI moiety. In order to study the solvent effect on ET, the spectra of the compounds in solution of HEX, TOL and ACN were measured. Species‐associated difference spectra (SADS) using sequential model were obtained by global fitting.

A positive absorption band centered at 410 nm was observed for **PI‐Rho** upon photoexcitation in TOL (Figure 5a), which is attributed to the ^1^PI* state. After 579 fs, new positive absorption bands centered at 530 and 580 nm emerged, which are attributed to PI^−.^ and RB^+.^, respectively (Figure 5b). As the time delay increased to 229 ps, the CT absorption signal is shifted from 530 to 490 nm, and it is assigned as a ^1^CT state. These bands are persistent in the time window of the set up (>3.3 ns). According to the global fitting and the resulted SADS, we tentatively propose that the rate constant for ^1^LE→^1^CT is 1.7×10^12^ s^−1^, and the rate constant for ^1^CT→^3^CT was 4.4×10^9^ s^−1^. **PI‐Rho** in ACN shows only one ESA band centered at 540 nm, which is attributed to CT state (Figure S40). This signal appears at almost zero time delay indicates that the CT is very fast, and CR is also fast (the CT state lifetime: 10 ps) in ACN that is why there is no triplet state observed in ns‐TA spectrum.

For **PI‐Rho‐S** in TOL, an ESA band centered at 415 nm was observed (Figure 5d), which is attributed to singlet‐excited state (^1^PI*). Then a positive absorption band centered at 480 and 637 nm emerged after 372 fs, which is attributed to PI^−.^ (Figure 5e). After 5 ps, the PI^−.^ signal shifted from 480 to 430 nm. The lifetime of the final species is much longer than maximum time range of the spectrometer (3.3 ns) which suggested this species is a ^3^CT state. According to the global analysis, the rate constant for ^1^LE→^1^CT is 2.7×10^12^ s^−1^, the rate constant for ^1^CT→^3^CT is 2.0×10^11^ s^−1^. **PI‐Rho‐S** in ACN shows similar positive absorption bands at 470 nm and 620 nm, which are attributed to CT state (3.6 ps). CR in ACN takes much longer time than the maximum time range of the spectrometer (>3.3 ns. Figure S39).

**Figure 6:**
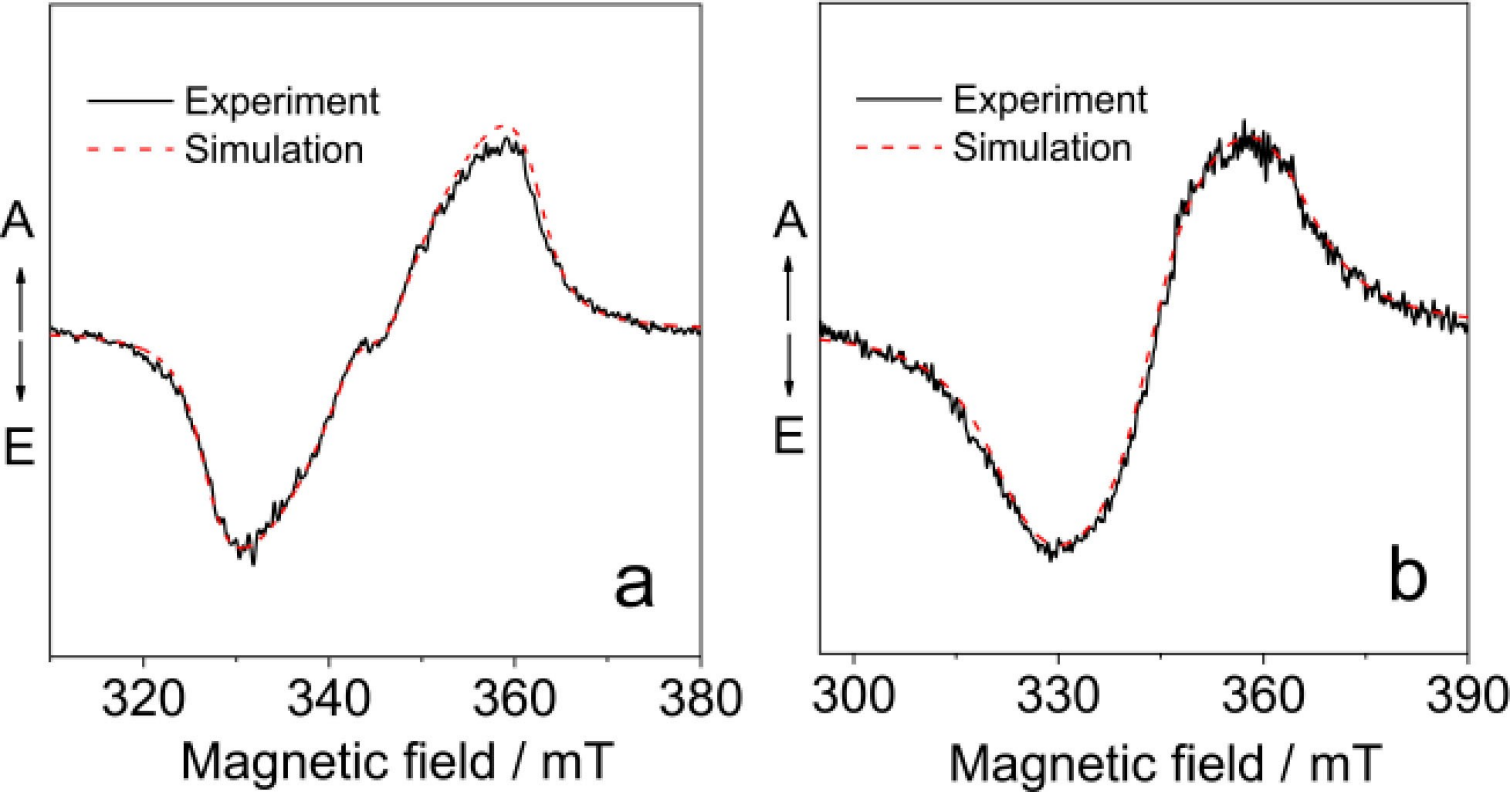
TREPR spectra of a) **PI‐Rho** and b) **PI‐Rho‐S**. Determined with X‐band EPR spectrometer, at 85 K. The delay time is 0.6 μs following a 355 nm laser pulse, *c*=5.0×10^−4^ M in mixed solvent TOL/2‐MeTHF (3/1, v/v). The red lines are computer simulations of the triplet‐state spectra with parameters supplied in Table 4.

In order to study the electron spin multiplicity of the CT state, pulsed laser excited TREPR spectra were studied (Figure 6).[^2^, ^4^, ^25^, ^44^, ^45^, ^46^] For the dyads, structureless TREPR spectra with *E*/*A* phase pattern were observed (*E* stands for emissive and *A* stands for enhanced absorption). No sharp peaks at canonical orientation were observed. This spectral feature is different from SCRP with very small exchange interaction (*J*) values.^[20]^ The spectrum is originated from a ^3^CT state.[^47^, ^48^] Simulation show that there is a dominant species with unusually small zero field splitting (ZFS) *D* parameter, i.e. |*D*|=540 MHz or 600 MHz for **PI‐Rho** and **PI‐Rho‐S**, respectively (Table 4). The ZFS |*E*| values are 148 MHz and 162 MHz, respectively. This is unusual for a small chromophore as PI because the triplet state with wave function confined on a small π‐conjugation system should show a large *D* value (ca. 3230 MHz). Previously the triplet state TREPR spectra of native PI chromophore was measured, the ZFS |*D*| and |*E*| values are 3234 MHz and 898 MHz, respectively.^[49]^ In comparison, the |*D*|value of naphthalimide is in the range of 1428–2590 MHz,[^31^, ^50^] and the triplet state of naphalenediimide shows a |*D*| value of 2225 MHz.^[31]^ Therefore, the transient species we observed is attributed to a ^3^CT state.^[51]^ No TREPR spectral signal was detected in fluid solution, i.e. no polarized radicals is formed (without any electron spin‐spin interaction).

**Table 4:**
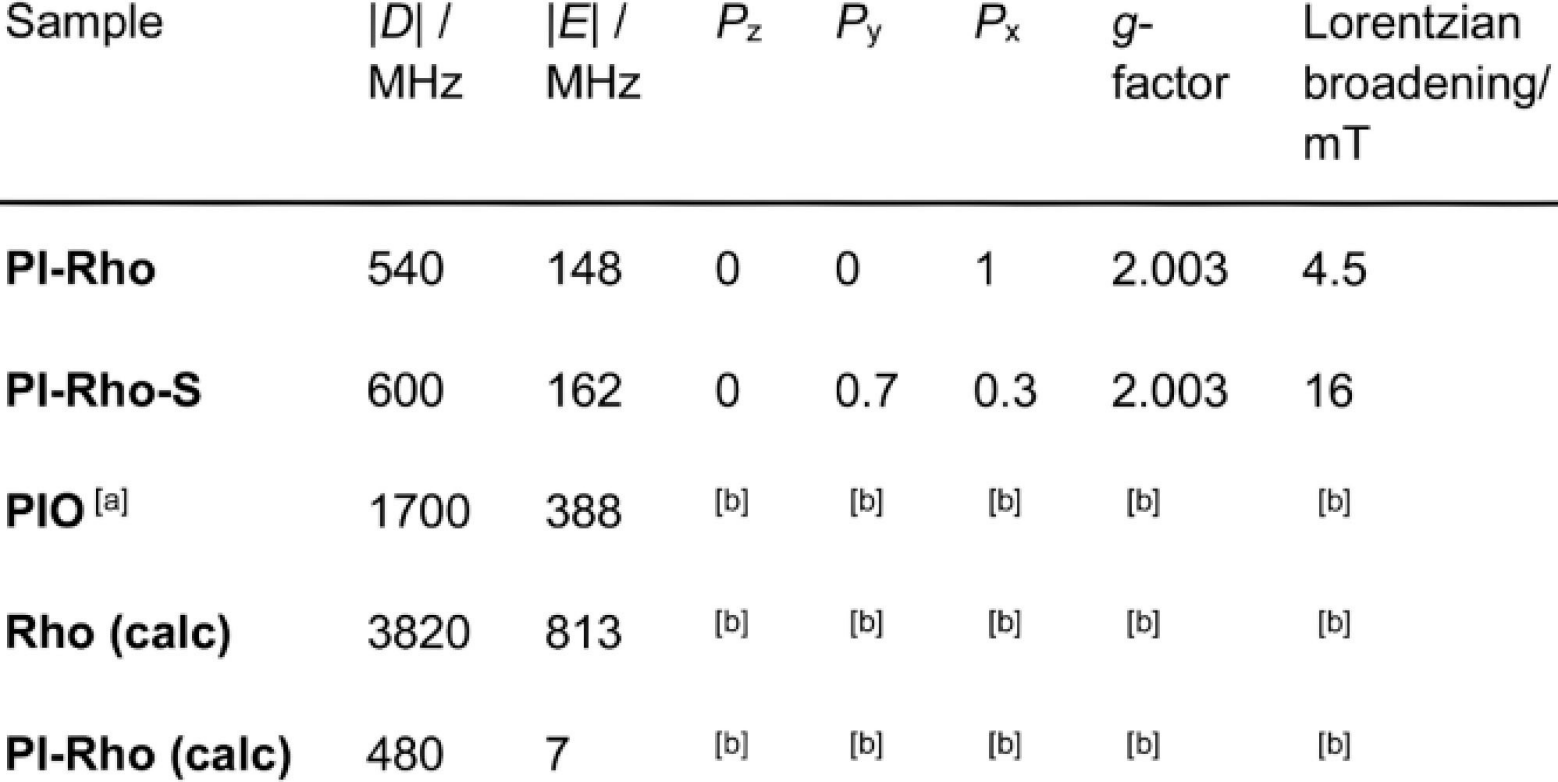
Experimental ZFS parameter, population rates of the three sublevels of the triplet states and *g*‐factor of the dyads. Calculated ZFS parameters of a dyad and of the reference compounds.

[a] An oxygen atom of one carboxy group of a PI molecule has been substituted with two methyl groups. [b] Not studied.

**Figure 7:**
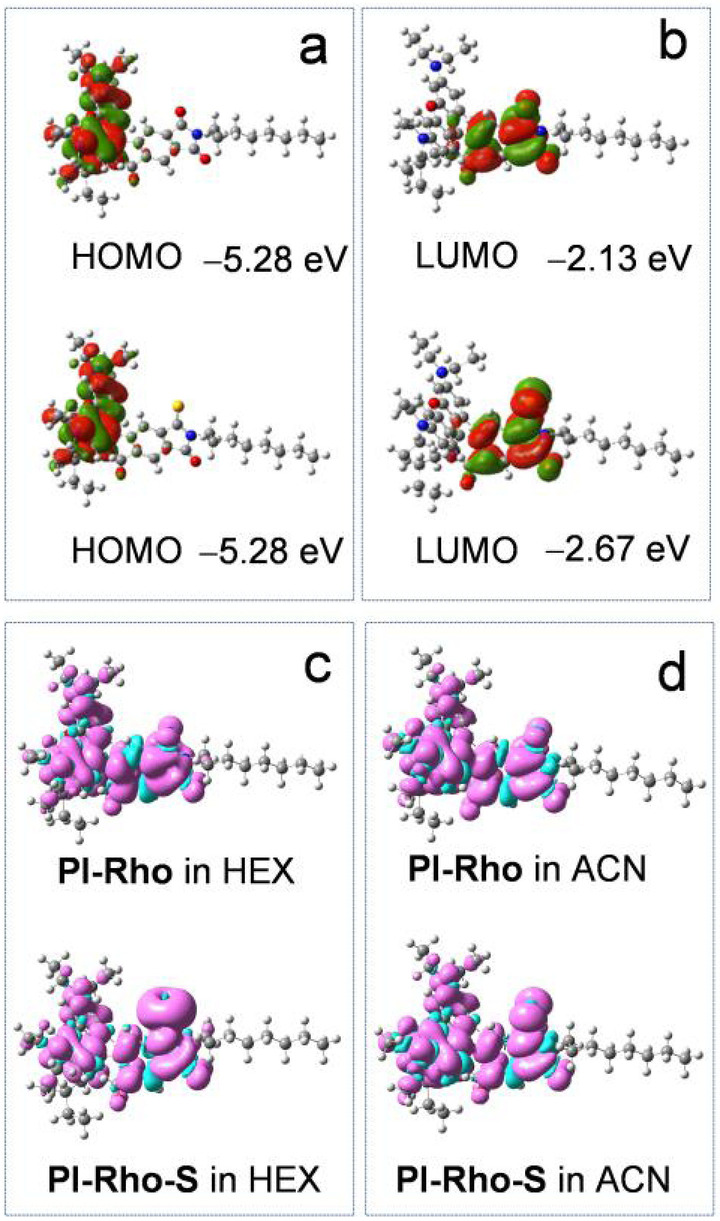
Contour map and energy [eV] of the a) HOMO and b) LUMO of **PI‐Rho** (upper row in each frame) and **PI‐Rho‐S** (bottom row in each frame) at the optimized S_0_ geometry. Triplet state spin density surfaces of **PI‐Rho** (upper row in each frame) and **PI‐Rho‐S** (bottom row in each frame) in c) HEX and d) ACN (CPCM model) (isovalue=0.0004 a.u.). Calculated by DFT at B3LYP/6‐31G(d) level with Gaussian 09.

Calculation on a putative reference molecule (**PIO**, Scheme 1, resembling the PI group in **PI‐Rho**) gives |*D*| of 1700 MHz (see Table 4), which is 2‐ or 3‐fold that of the experimental values observed for **PI‐Rho** and **PI‐Rho‐S**. Therefore, the transient species we observed with the TREPR spectra is not a ^3^LE state localized on the PI moieties. As for the **Rho**, the calculation shows that in this small molecule the ZFS parameters are also larger, with |*D*|=3820 MHz (see Table 4). The Highest Occupied Molecular Orbital–Lowest Unoccupied Molecular Orbital (HOMO–LUMO) electronic configuration describes a CT state for the **PI‐Rho** dyad (see Figure 7). The calculation of the ZFS parameters of this state leads to |*D*|=480 MHz, which is close to the experimental result (|*D*|=540 MHz for **PI‐Rho**). We can then conclude that the experimental *D*‐value found for **PI‐Rho** dyad is fully compatible with a CT state.

The optimization of the S_0_ gives dihedral angles between the π‐planes of the xanthene and PI moieties of 90° for both **PI‐Rho** and **PI‐Rho‐S**, which are close to the single crystal X‐ray diffraction analysis (87°). For **PI‐Rho**, the HOMO is confined on the xanthene moiety (Figure 7a), and the LUMO is confined on the PI moiety. Similar results were observed for **PI‐Rho‐S** (Figure 7b). Note the energy of LUMO of **PI‐Rho‐S** is −2.67 eV, which is lower by −0.54 eV than **PI‐Rho** that of (Figure 7).

The molecular geometry for **PI‐Rho** and **PI‐Rho‐S** doesn't change at excited states (Figure S49), as compared to the S_0_. This is the advantage of the fully rigid *spiro*‐dyad for formation of long‐lived CT state, because with the rigid structure, the inner‐reorganization is minimized. Moreover, the compact dyad structure will minimize the outer‐shell‐reorganization energy, thus the total reorganization energy will be minimized, as a result, the CR will easily go to the Marcus inverted region.

The electron spin density surfaces are delocalized on both moieties in the dyads. The xanthene moiety is with triplet state energy (3.42 eV, for **RB‐C**) much higher than the PI moiety in **PI‐Rho** (3.14 eV for **PIO**; 2.16 eV for **PIS**, by TDDFT computation, Table S5), and both are much higher than the T_1_ state of the dyad (2.61 eV for **PI‐Rho**, and 1.97 eV for **PI‐Rho‐S**), therefore, the delocalized triplet states should be a ^3^CT state, not ^3^RB/^3^PI states in equilibrium.[^52^, ^53^] We studied a previously reported anthraquinone‐phenothiazine dyads with similar approach (Figure S52).^[20]^ In THF, the T_1_ state is predicted as a ^3^CT state, whereas in TOL, the T_1_ state was predicted as a ^3^LE state, which are fully consistent with the previous experimental observations.^[20]^ The spin density of radical cations and radical anions were computed (Figure S50). The radical cation is confined on the xanthene part, and the radical anion is confined on the PI or the thionated PI part. These results are in agreement with the ns‐TA and fs‐TA spectral studies.

**Scheme 2:**
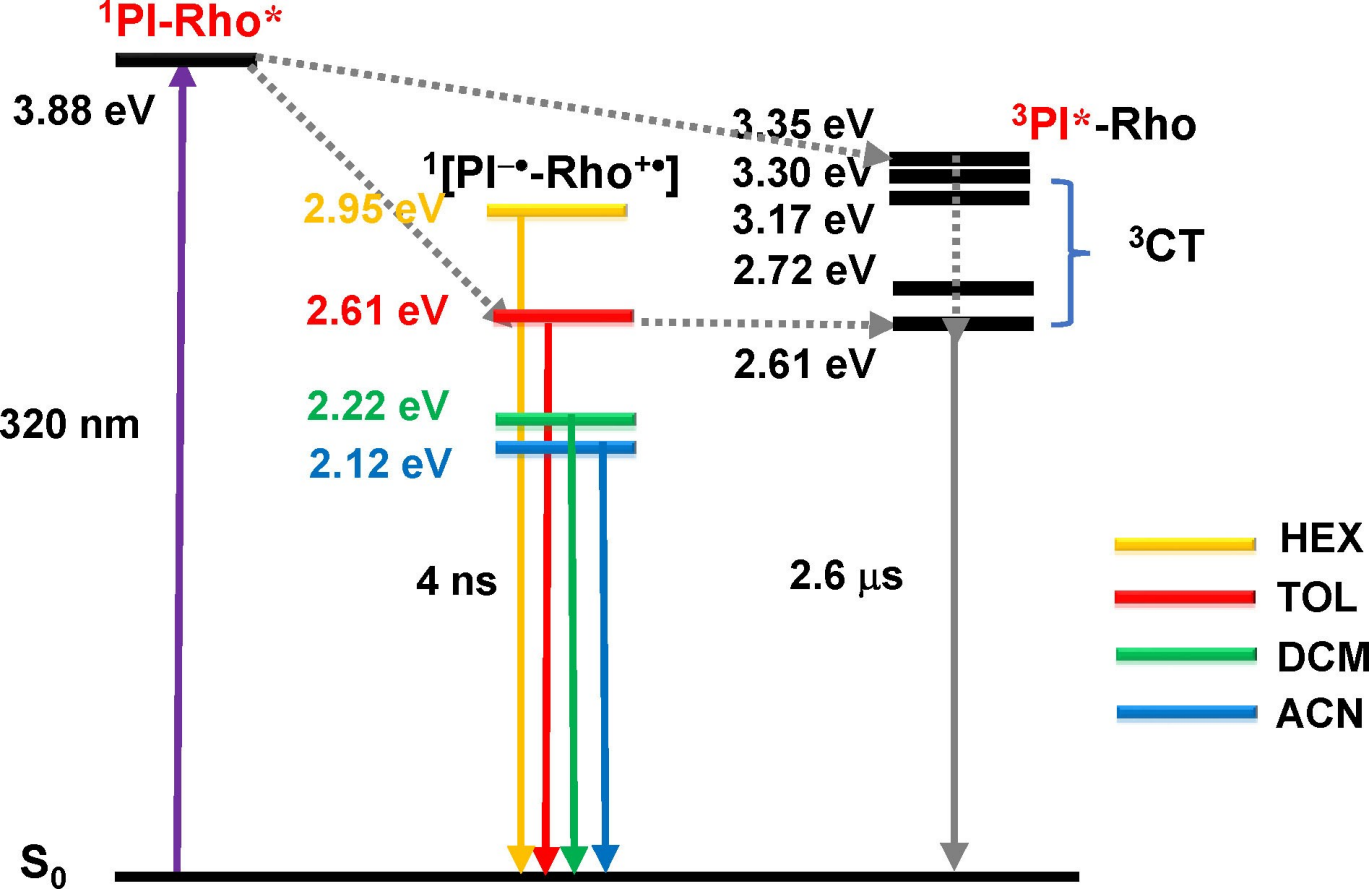
Simplified Jablonski diagram illustrating the photophysical processes involved in **PI‐Rho**. ^1^ PI‐Rho* energy level was obtained by the crossing point of the normalized LE state UV/Vis absorption and fluorescence spectra.^1^CT energy levels were calculated based on UV/Vis CT absorption band and CT fluorescence spectra in HEX and TOL. ^1^CT energy levels were calculated based on the electrochemical data in DCM and ACN. TDDFT calculations were performed at the B3LYP/6‐31G(d) level in vacuum by using Gaussian 09.

The photophysical processes of PI‐Rho is summarized in Scheme 2. The singlet state energy of the PI moiety in PI‐Rho is 3.88 eV (approximated with the red end of the LE absorption; 3.78 eV according to the TDDFT computation). The ^1^CT state energy was approximated as 2.61 eV by the UV/Vis absorption ^1^PI^*^ is populated, and the fast CT occurs. The ns‐TA spectra demonstrated the formation of long‐lived CT state, the TREPR spectra support a ^3^CT assignment. The triplet state of native PI chromophore is 2.45 eV (determined by phosphorescence method).^[31]^ For the PI moiety in PI‐Rho (note there are only three carbonyl groups), the T_1_ state energy was approximated as 2.20 eV by phosphorescence method (Figure S28). Based on the CT state energy determined by electrochemical/optical spectral methods (2.12–2.95 eV), observation of the CT state in ns‐TA spectra is reasonable. The long lifetime (2.6 μs) of the CT state in the compact electron donor‐acceptor can be explained by the electron spin control, because the CR process of ^3^CT→S_0_ is electron spin forbidden.[^4^, ^17^, ^18^, ^22^, ^54^]

## Conclusion

In summary, we prepared two spiro novel rhodamine compounds with pyromellitimide (PI). In these compact electron donor–acceptor dyads, the closed‐ring rhodamine (xanthenes) unit is the electron donor, the PI or the thionated PI act as the electron acceptor. The electron donor and acceptor parts are connected by a *spiro* quaternary carbon atom, thus the torsion of the donor and acceptor parts is completely inhibited, such a fully rigid geometry is beneficial to form triplet charge transfer state (^3^CT state). Although the electron donor and acceptor parts are separated by two σ‐bands, the electronic coupling between the two parts in demonstrated by CT absorption and emission bands. Femtosecond transient absorption spectra indicated the charge separation takes 0.58 ps. Nanosecond transient absorption spectra show the formation of the CT state, with lifetimes of 2.6 μs. In the dyad with the thionated PI unit, the CT lifetime is shortened to 289 ns. Time‐resolved electron paramagnetic resonance (TREPR) spectra demonstrated the spin multiplicity of the CT state as triplet (^3^CT state), which shows much smaller zero field splitting (ZFS) |*D*| parameter (540 MHz) than the related localized triplet state (^3^LE), for which the |*D*| parameters are in the range of 1700 –3234 MHz. Thus, the long CT state lifetime is attributed to the electron spin control effect. Our results are useful for design of compact electron donor–acceptor dyads to access the long‐lived CT state.

## Conflict of interest

The authors declare no conflict of interest.

1

## Supporting information

As a service to our authors and readers, this journal provides supporting information supplied by the authors. Such materials are peer reviewed and may be re‐organized for online delivery, but are not copy‐edited or typeset. Technical support issues arising from supporting information (other than missing files) should be addressed to the authors.

Supporting Information

Supporting Information

## Data Availability

The data that support the findings of this study are available in the Supporting Information of this article.

## References

[1] D. Gust , T. A. Moore , A. L. Moore , Acc. Chem. Res. 1993, 26, 198–205.

[2] H. Levanon , J. R. Norris , Chem. Rev. 1978, 78, 185–198.

[3] E. Vauthey , ChemPhysChem 2012, 13, 2001–2011. 22473631 10.1002/cphc.201200106

[4] J. Verhoeven , J. Photochem. Photobiol. C 2006, 7, 40–60.

[5] J. W. Verhoeven , H. J. van Ramesdonk , M. M. Groeneveld , A. C. Benniston , A. Harriman , ChemPhysChem 2005, 6, 2251–2260. 16273579 10.1002/cphc.200500029

[6] T. Takada , K. Kawai , M. Fujitsuka , T. Majima , Proc. Natl. Acad. Sci. USA 2004, 101, 14002. 15381780 10.1073/pnas.0402756101 PMC521111

[7] E. H. Yonemoto , R. L. Riley , Y. I. Kim , S. J. Atherton , R. H. Schmehl , T. E. Mallouk , J. Am. Chem. Soc. 1992, 114, 8081–8087.

[8] D. I. Schuster , P. Cheng , P. D. Jarowski , D. M. Guldi , C. Luo , L. Echegoyen , S. Pyo , A. R. Holzwarth , S. E. Braslavsky , R. M. Williams , G. Klihm , J. Am. Chem. Soc. 2004, 126, 7257–7270. 15186163 10.1021/ja038676s

[9] N. J. Turro , J. C. Scaiano , Principles of Molecular Photochemistry: An Introduction, University Science Books, Sausalito, 2009.

[10] D. M. Guldi , Chem. Commun. 2000, 321–327.

[11] J. Hankache , O. S. Wenger , Chem. Eur. J. 2012, 18, 6443–6447. 22532315 10.1002/chem.201200199

[12] S. Fukuzumi , H. Kotani , K. Ohkubo , S. Ogo , N. V. Tkachenko , H. Lemmetyinen , J. Am. Chem. Soc. 2004, 126, 1600–1601. 14871068 10.1021/ja038656q

[13] S. Fukuzumi , Pure Appl. Chem. 2007, 79, 981–991.

[14] K. Ohkubo , H. Kotani , J. Shao , Z. Ou , K. M. Kadish , G. Li , R. K. Pandey , M. Fujitsuka , O. Ito , H. Imahori , S. Fukuzumi , Angew. Chem. Int. Ed. 2004, 43, 853–856; 10.1002/anie.200352870 14767957

[15] X. Zhang , X. Chen , J. Zhao , Dalton Trans. 2021, 50, 59–67. 33338095 10.1039/d0dt03737k

[16] J. E. McGarrah , Y. J. Kim , M. Hissler , R. Eisenberg , Inorg. Chem. 2001, 40, 4510–4511. 11511190 10.1021/ic015559u

[17] S. Suzuki , R. Sugimura , M. Kozaki , K. Keyaki , K. Nozaki , N. Ikeda , K. Akiyama , K. Okada , J. Am. Chem. Soc. 2009, 131, 10374–10375. 19722616 10.1021/ja904241r

[18] B. Geiß , C. Lambert , Chem. Commun. 2009, 1670–1672. 10.1039/b820744e 19294257

[19] S.-H. Lee , C. T.-L. Chan , K. M.-C. Wong , W. H. Lam , W.-M. Kwok , V. W.-W. Yam , J. Am. Chem. Soc. 2014, 136, 10041–10052. 24988327 10.1021/ja5040073

[20] A. Karimata , H. Kawauchi , S. Suzuki , M. Kozaki , N. Ikeda , K. Keyaki , K. Nozaki , K. Akiyama , K. Okada , Chem. Lett. 2013, 42, 794–796.

[21] S. Chakraborty , T. J. Wadas , H. Hester , R. Schmehl , R. Eisenberg , Inorg. Chem. 2005, 44, 6865–6878. 16180842 10.1021/ic0505605

[22] D. Liu , A. M. El-Zohry , M. Taddei , C. Matt , L. Bussotti , Z. Wang , J. Zhao , O. F. Mohammed , M. Di Donato , S. Weber , Angew. Chem. Int. Ed. 2020, 59, 11591–11599; 10.1002/anie.202003560 PMC7496792 32270586

[23] D. J. Gibbons , A. Farawar , P. Mazzella , S. Leroy-Lhez , R. M. Williams , Photochem. Photobiol. Sci. 2020, 19, 136–158. 31995088 10.1039/c9pp00399a

[24] M. A. Filatov , Org. Biomol. Chem. 2020, 18, 10–27. 10.1039/c9ob02170a 31750502

[25] Y. Hou , X. Zhang , K. Chen , D. Liu , Z. Wang , Q. Liu , J. Zhao , A. Barbon , J. Mater. Chem. C 2019, 7, 12048–12074.

[26] V.-N. Nguyen , Y. Yan , J. Zhao , J. Yoon , Acc. Chem. Res. 2021, 54, 207–220. 33289536 10.1021/acs.accounts.0c00606

[27] M. Lv , Y. Yu , M. E. Sandoval-Salinas , J. Xu , Z. Lei , D. Casanova , Y. Yang , J. Chen , Angew. Chem. Int. Ed. 2020, 59, 22179–22184; 10.1002/anie.202009439 32840046

[28] X. Chen , Y. Zhou , X. Peng , J. Yoon , Chem. Soc. Rev. 2010, 39, 2120–2135. 20502801 10.1039/b925092a

[29] X. Zhang , Y. Xiao , X. Qian , Angew. Chem. Int. Ed. 2008, 47, 8025–8029; 10.1002/anie.200803246 18792904

[30] H. Yu , Y. Xiao , H. Guo , X. Qian , Chem. Eur. J. 2011, 17, 3179–3191. 21312299 10.1002/chem.201002498

[31] G. P. Wiederrecht , W. A. Svec , M. R. Wasielewski , T. Galili , H. Levanon , J. Am. Chem. Soc. 2000, 122, 9715–9722.

[32] Q. Mi , E. T. Chernick , D. W. McCamant , E. A. Weiss , M. A. Ratner , M. R. Wasielewski , J. Phys. Chem. A 2006, 110, 7323–7333. 16759120 10.1021/jp061218w

[33] N. Pearce , E. S. Davies , R. Horvath , C. R. Pfeiffer , X.-Z. Sun , W. Lewis , J. Mc Master , M. W. George , N. R. Champness , Phys. Chem. Chem. Phys. 2018, 20, 752–764. 29139504 10.1039/c7cp06952a

[34] V.-N. Nguyen , S. Qi , S. Kim , N. Kwon , G. Kim , Y. Yim , S. Park , J. Yoon , J. Am. Chem. Soc. 2019, 141, 16243–16248. 31577431 10.1021/jacs.9b09220

[35] M. Hussain , J. Zhao , W. Yang , F. Zhong , A. Karatay , H. G. Yaglioglu , E. A. Yildiz , M. Hayvali , J. Lumin. 2017, 192, 211–217.

[36] M. H. Lee , H. J. Kim , S. Yoon , N. Park , J. S. Kim , Org. Lett. 2008, 10, 213–216. 18078343 10.1021/ol702558p

[37] Deposition Number 2075565 contains the supplementary crystallographic data for this paper. These data are provided free of charge by the joint Cambridge Crystallographic Data Centre and Fachinformationszentrum Karlsruhe Access Structures service.

[38] S. Sasaki , K. Hattori , K. Igawa , G.-i. Konishi , J. Phys. Chem. A 2015, 119, 4898–4906. 25915152 10.1021/acs.jpca.5b03238

[39] S. Fukuzumi , Org. Biomol. Chem. 2003, 1, 609–620. 12929444 10.1039/b300053b

[40] M. T. Colvin , A. B. Ricks , A. M. Scott , D. T. Co , M. R. Wasielewski , J. Phys. Chem. A 2012, 116, 1923–1930. 22296165 10.1021/jp212546w

[41] S. Kumar , M. R. Ajayakumar , G. Hundal , P. Mukhopadhyay , J. Am. Chem. Soc. 2014, 136, 12004–12010. 25093533 10.1021/ja504903j

[42] K. Zimmer , M. Hoppmeier , A. Schweig , Chem. Phys. Lett. 1998, 293, 366–370.

[43] L. Ma , P. Hu , H. Jiang , C. Kloc , H. Sun , C. Soci , A. A. Voityuk , M. E. Michel-Beyerle , G. G. Gurzadyan , Sci. Rep. 2016, 6, 28510. 27346797 10.1038/srep28510 PMC4921923

[44] S. Weber , eMagRes 2017, 6, 255–270.

[45] S. Richert , C. E. Tait , C. R. Timmel , J. Magn. Reson. 2017, 280, 103–116. 28579096 10.1016/j.jmr.2017.01.005

[46] T. Biskup , Front. Chem. 2019, 7, 10. 30775359 10.3389/fchem.2019.00010 PMC6367236

[47] R. Carmieli , A. L. Smeigh , S. M. Mickley Conron , A. K. Thazhathveetil , M. Fuki , Y. Kobori , F. D. Lewis , M. R. Wasielewski , J. Am. Chem. Soc. 2012, 134, 11251–11260. 22676136 10.1021/ja303721j

[48] N. Zarrabi , B. J. Bayard , S. Seetharaman , N. Holzer , P. Karr , S. Ciuti , A. Barbon , M. Di Valentin , A. van der Est , F. D'Souza , P. K. Poddutoori , Phys. Chem. Chem. Phys. 2021, 23, 960–970. 33367389 10.1039/d0cp05783e

[49] N. Hirofumi , T. Masahide , H. Noboru , N. Satoshi , O. B. Atsuhiro , Bull. Chem. Soc. Jpn. 1995, 68, 2193–2202.

[50] G. Tang , A. A. Sukhanov , J. Zhao , W. Yang , Z. Wang , Q. Liu , V. K. Voronkova , M. Di Donato , D. Escudero , D. Jacquemin , J. Phys. Chem. C 2019, 123, 30171–30186.

[51] B. H. Drummond , N. Aizawa , Y. Zhang , W. K. Myers , Y. Xiong , M. W. Cooper , S. Barlow , Q. Gu , L. R. Weiss , A. J. Gillett , D. Credgington , Y.-J. Pu , S. R. Marder , E. W. Evans , Nat. Commun. 2021, 12, 4532. 34312394 10.1038/s41467-021-24612-9 PMC8313702

[52] C. Zhang , J. Zhao , S. Wu , Z. Wang , W. Wu , J. Ma , S. Guo , L. Huang , J. Am. Chem. Soc. 2013, 135, 10566–10578. 23790008 10.1021/ja405170j

[53] K. Chen , M. Hussain , S. S. Razi , Y. Hou , E. A. Yildiz , J. Zhao , H. G. Yaglioglu , M. Di Donato , Inorg. Chem. 2020, 59, 14731–14745. 32864961 10.1021/acs.inorgchem.0c01932

[54] J. Hankache , O. S. Wenger , Chem. Commun. 2011, 47, 10145–10147. 10.1039/c1cc13831f 21833397

