# Supplementary material for: Long‐Lived Charge‐Transfer State in Spiro Compact Electron Donor–Acceptor Dyads Based on Pyromellitimide‐Derived Rhodamine: Charge Transfer Dynamics and Electron Spin Polarization
Source: Angew Chem Int Ed Engl. 2022 Apr 21;61(33):e202203758. doi: 10.1002/anie.202203758 (PMC9543469; doi:10.1002/anie.202203758)
Supplement: Supplementary file 1 — Supporting Information [file ANIE-61-0-s001.pdf]

Angewandte Chemie
Eine Zeitschrift der Gesellschaft Deutscher Chemiker

Supporting Information

Long-Lived Charge-Transfer State in Spiro Compact Electron Donor–Acceptor Dyads Based on Pyromellitimide-Derived Rhodamine: Charge Transfer Dynamics and Electron Spin Polarization

X. Chen, A. A. Sukhanov, Y. Yan, D. Bese, C. Bese, J. Zhao, V. K. Voronkova*, A. Barbon*, H. G. Yaglioglu**

Table of Contents

1. Molecular Structure Characterization Data……………………………………………………….……..……3

2. Crystal Data of **PI-Rho**……………………………………………………………………...............................16

3. UV-Vis Absorption and Fluorescence Emission Spectra……………...………………………………….........17

4. Fluorescence Lifetime and Fluorescence lifetime and phosphorescence lifetime Spectra…………....20

5. Nanosecond Transient Absorption Spectra……………………………………………………………..…24

6. Femtosecond Transient Absorption Spectra…………………………………………………………………28

7. Absolute fluorescence quantum yields………………………………………………………….…………32

8. Chemical reduction absorption spectra…………………….…………….……………….……………………33

9. Cyclic Voltammogram of the Compounds and Spectroelectrochemistry…………………….…………33

10. DFT Calculations………………………………………………………………………………….…………35

References……………..…………………………………………………………………………….................42

1. Molecular Structure Characterization Data.

General Methods. UV-vis absorption spectra were measured on UV–2550 UV–vis spectrophotometer (Shimadzu Ltd., Japan). Fluorescence spectra were recorded on FS5 Fluorescence/phosphorescence spectrometer (Edinburgh Instruments Ltd., U. K.) Luminescence lifetimes were recorded on an Edinburgh OB920 fluorescence/ phosphorescence lifetime spectrometer (Edinburgh Instruments Ltd., U. K.). ^{1}H NMR and ^{13}C NMR spectra were recorded on the Bruker Avance spectrometers (400 MHz). ^{1}H and ^{13}C chemical shifts were reported in parts per million (ppm) relative to TMS, with the residual solvent peak used as an internal reference. The mass spectra were measured by HRMS.

Nanosecond Transient Absorption Spectroscopy. Nanosecond time-resolved transient difference absorption spectra were recorded on a LP920 laser flash photolysis spectrometer (Edinburgh Instruments Ltd., UK). The solutions were purged with N_2 for 30 min before measurement. The samples were excited with a nanosecond pulsed laser at 355 nm. The data was analyzed with the L900 software.

Ultrafast Transient Absorption Spectroscopy. A Ti:sapphire laser amplifier followed by an optical parametric amplifier system (Spectra Physics; Spitfire Pro XP; TOPAS) with 52 fs pulse duration, 1 kHz repetition rate and a commercial pump probe experimental setup (Spectra Physics; Helios) with a white light continuum probe were used for the experiments. The pulse duration was measured as 120 fs by cross-correlation inside the pump probe setup. Wavelength of the pump beam was chosen according to the steady-state absorption spectra of studied compounds. A 2-mm thick cuvette containing the sample solution was used for pump probe experiments. The experimental data were analyzed by using the Surface Xplorer software, which is supported by Ultrafast Systems. All the experiments were performed with magic angle (54.7 degree) between pump and probe polarization directions.

Time-Resolved (TR) EPR Spectroscopy. The TR EPR measurements were performed on an X-bands EPR Elexsys E-580 spectrometer (Bruker) with the dielectric ring X-Band ER 4118X-MD5-W1 resonator. TR EPR can be performed using transient mode of spectrometer. The signal coming from the detector (diode) is digitized by a SpecJet ultra-fast transient signal average. The bandwidth of the video amplifier is 20 MHz. Laser excitation at 355 nm was achieved by an Nd:YAG laser (LQ 629 Solar LS) with the repetition rate of 100 Hz and energy of 1 mJ. The samples were dissolved in MeTHF/toluene (1/3, v/v) at 0.1 mM and transferred into 5 mm OD, 3.0 mm ID quartz tubes. The low temperature spectra of the triplet state were simulated using the EasySpin package based on MATLAB (function pepper).[54] Fitting of experimental data has been done using MATLAB's routine fmincon.

Theoretical Computations. Calculations of the ZFS parameters were conducted by using the point dipole approximation. The coefficients of the LCAO of the HOMO and LUMO molecular orbitals were obtained by an AM1 calculation.

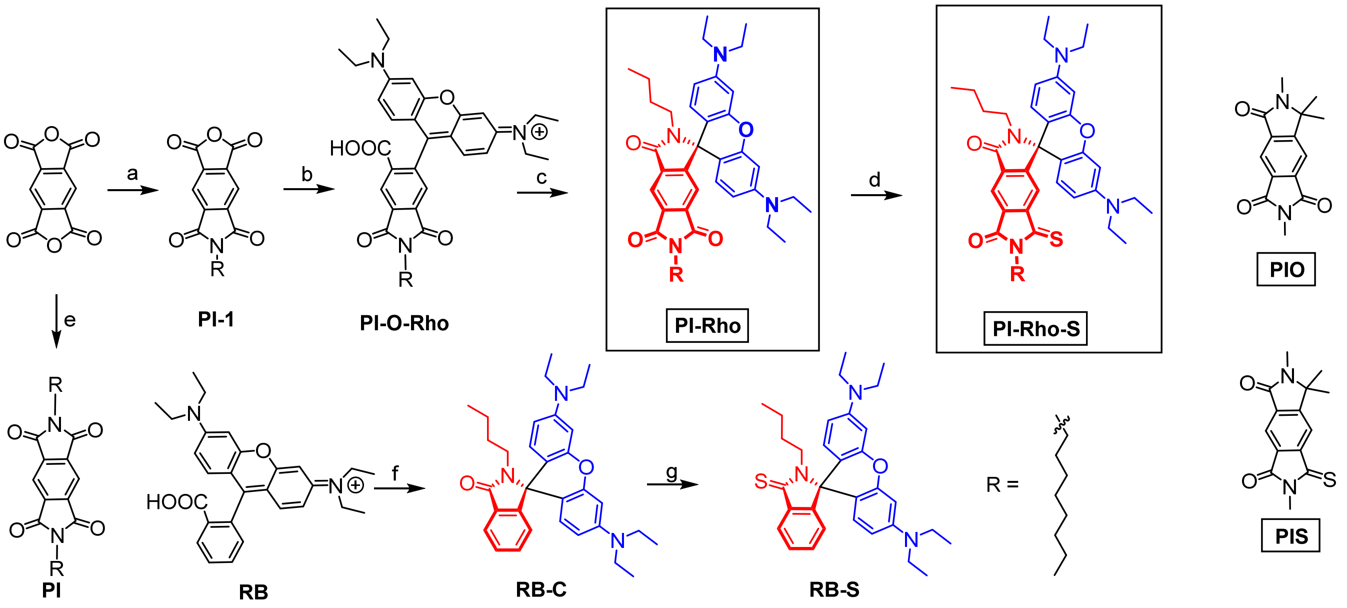

Scheme S1. Synthesis of RB Derivatives. Keys: (a) *n*-Octylamine, DMF, 80 °C, 10 h, under N_2, yield: 30%; (b) 3-Diethylaminophenol, H_2SO_4, 180 °C, 4 h, under N_2, yield: 35%; (c) $POCl_3$, 1,2-dichloroethane, 80 °C, 6 h; *n*-butylamine, CH_3CN, Et_3N, reflux, 25 h; yield: 40%; (d) Lawesson's Reagent, *p*-xylene, 150 °C, 3 h, under N_2, yield: 20%; (e) Similar to step (a), yield: 81%; (f) Similar to step (c), yield: 90%; (g) Similar to step (d), yield: 95%.

Synthesis of compound PI-1 and PI. Pyromellitic Dianhydride (9.2 mmol, 2.00 g) was added into a 100 mL two-neck bottle and dry DMF (35 mL) was added under N_2 atmosphere. The mixture was heated at 80°C, *n*-octylamine (1.2 mL, 9.2 mmol) with dry DMF (15 mL) were added into the mixture within an hour, then the temperature increased 110°C for 11 h. After the reaction solution was cooled to room temperature, put the reaction solution in the refrigerator and stand overnight. The solid was collected by filtration, which is a disubstituted product (**PI-1**). DMF in the filtrate was distilled off under reduced pressure. Then dissolve the viscous mixture in dichloromethane (15 mL), the un-reacted anhydride was removed by filtration. The solvent was removed by rotary evaporator. Then disubstituted product (**PI-1**) was purified by column chromatography (DCM/PE = 1/1) to give a white solid, the compound (PI) was purified by column chromatography (DCM to DCM/MEOH 20:1 gradient elution) to give a white solid. **PI** was obtained, yield: 1.10 g (38%). ^{1}H NMR (600 MHz, $CDCl_3$, ppm) δ: 8.26 (s, 2H), 3.75 –3.71 (m, 4H), 1.71–1.68 (m, 4H), 1.33–1.26 (m, 20H), 0.89–0.85 (m, 6H); APCI–HRMS ($[C_{26}H_{36}N_2O_4$+ H$]^+$): calcd, m/z = 441.2675; found, m/z = 441.2749. **PI-1** was obtained, yield: 0.92 g (30%). ^{1}H NMR (600 MHz, $CDCl_3$, ppm) δ: 8.11 (s, 1H), 8.08 (s, 1H), 3.68–3.66 (m, 2H), 1.66 (s, 2H), 1.32–1.26 (m, 10H), 0.88–0.85 (m, 3H). TOF–HRMS ($C_{18}H_{19}NO_5$): calcd, m/z = 329.1263; found, m/z = 329.1274.

Synthesis of compound PI-O-Rho.

m-Cresole (0.65 g, 6 mmol), **PI-1** (0.92, 2.79 mmol) and concentrated sulfuric acid (3 mL) were heated at 80 °C for 1 h under N_2 atmosphere, then heated to 180°C within 30 minutes, and reacted for 4 h. After cooling of the reaction

mixture to room temperature, the reaction mixture was poured into ice-water mixture (22 mL), stirred vigorously, and put it in the refrigerator to stand overnight. The viscous solid was collected by filtration, the solid was added to ammonia water (10 mL) (NH_3/H_2O = 1/4), concentrated hydrochloric acid was added to bring pH < 2, under ice bath condition. The solid was collected by filtration. Then the solid was dissolved in DCM, the organic solution was dried over anhydrous Na_2SO_4. The crude product was purified by column chromatography (silica gel, DCM/ MeOH = 10/1, v/v). **PI-O-Rho** was obtained as purple solid. Yield: 610 mg (35%). ^{1}H NMR (600 MHz, $CDCl_3$, ppm) δ: 8.58 (s, 1H), 7.61 (s, 1H), 6.67 (s, 2H), 6.57–6.53 (d, 4H), 3.70 (s, 2H), 3.46 (s, 8H), 2.04 (s, 6H), 1.68 (s, 6H), 0.88 (s, 15H); ^{13}C NMR (125 MHz, $CDCl_3$): δ = 167.61, 167.45, 166.12, 159.82, 153.23, 149.07, 136.25, 135.61, 131.92, 128.44, 119.22, 118.26, 108.16, 103.77, 98.08, 65.49, 44.43, 40.69, 31.75, 30.14, 29.12, 28.55, 26.84, 22.60, 20.37, 14.05, 13.56, 12.54. ESI–HRMS ($[C_{38}H_{46}N_3O_5 + H]^+$): calcd, m/z = 624.3432; found, m/z = 624.3438.

Synthesis of compound PI-Rho.

Under N_2 atmosphere, **PI-O-Rho** (300 mg, 0.5 mmol) were dissolved in dry 1,2-dichloroethane (5 mL), then $POCl_3$ (0.2 ml) was added dropwise into the mixture, the mixture was stirred and refluxed for 6 h. After cooling to room temperature, the solvent was removed under reduced pressure, the remaining mixture was dissolved in dry acetonitrile (10 mL). *n*-Butylamine (2 mL) and Et_3N (0.3 mL) were added dropwise to the mixture at room temperature under N_2 atmosphere, the mixture was refluxed for 25 h. The solvent was evaporated under reduced pressure. The product was purified by column chromatography (silica gel, DCM/MeOH from 1/0 to 20/1, v/v) to give a yellow solid. Yield: 130 mg (40%). Mp: 156.2–157.0 °C. ^{1}H NMR (400 MHz, $CDCl_3$): δ = 8.31 (s, 1H), 7.48 (s, 1H, 6.41 (s, 4H), 6.28 (s, 2H), 3.65–3.63 (m, 2H), 3.35–3.16 (m, 8H), 3.16 (s, 2H), 1.64–1.58 (m, 6H), 1.29–1.25 (m, 5H), 1.24 (s, 3H), 1.18 (m, 14H), 0.87–0.84 (m, 3H), 0.72–0.69 (m, 3H). ^{13}C NMR (125 MHz, $CDCl_3$): δ = 167.61, 167.45, 166.12, 159.82, 153.23, 149.07, 136.25, 135.61, 131.92, 128.44, 119.22, 118.26, 108.16, 103.77, 98.08, 65.49, 44.43, 40.69, 31.75, 30.14, 29.12, 28.55, 26.84, 22.60, 20.37, 14.05, 13.56, 12.54. ESI-HRMS($[C_{42}H_{54}N_4O_4+H]^+$): calcd. m/z = 679.4145; found m/z = 679.4213.

Synthesis of compound PI-Rho-S*.*

Under N_2 atmosphere, **PI-Rho** (100 mg, 0.15 mmol) and Lawesson's reagent (198 mg, 0.5 mmol) were dissolved in dry *p*-xylene (18 mL), then the mixture was stirred at 150°C for 3 h. After reaction, saturated $NaHCO_3$ aqueous solution (30 mL) was added, the mixture was extracted with ethyl acetate (3×10 mL). The brown organic layers were combined and MeOH (100 mL) was added. The solvent was evaporated under reduced pressure. The crude product was purified using column chromatography (silica gel, DCM: PE = 1:2, v/v) to give an orange solid. Yield: 20 mg (50%). Mp: 195.6 – 195.9 °C. ^{1}H NMR (400 MHz, $CDCl_3$): δ = 8.52 (s, 1H), 7.61 (s, 1H), 1.26–1.17 (m, 17H), 1.12–1.09 (m, 2H), 0.95–0.92 (m, 3H), 0.72–0.69 (m, 3H). ^{13}C NMR (125 MHz, $CDCl_3$): δ = 195.93, 188.18, 168.66, 157.05, 153.11, 149.33, 141.72, 139.75, 128.75, 127.75, 120.12, 119.42, 108.35, 102.49, 98.04, 73.72, 45.10, 44.47, 41.17, 31.59, 29.94, 28.73, 22.65, 20.43, 20.17, 14.12, 13.69, 13.48, 12.50. ESI-MS($[C_{42}H_{54}N_4O_3S+H]^+$): calcd. m/z = 695.39; found m/z = 695.51.

Synthesis of compound RB-C.

Under N_2 atmosphere, **RB** (100 mg, 0.23 mmol) were dissolved in dry 1,2-dichloroethane (5 mL), then $POCl_3$ (0.1 mL) was added dropwise to the mixture. Then the reaction mixture was stirred and refluxed for 6 h. After cooling

to room temperature, the solvent was removed by rotary evaporation, the remaining mixture was dissolved in dry acetonitrile (10 mL). Then under N_2 atmosphere, *n*-butylamine (1 mL) and Et_3N (0.3 mL) were added dropwise to the mixture at room temperature, the mixture was refluxed for 25 h. The solvent was evaporated under reduced pressure. The product was purified by column chromatography (silica gel, DCM/PE = 1/1) to give a pink solid. Yield: 101 mg (90%). ^{1}H NMR (400 MHz, DMSO): δ = 7.78–7.76 (m, 1H), 7.51–7.49 (m, 2H), 7.05–7.03(m, 1H), 6.38–6.35 (m, 4H), 6.30 (s, 1H), 6.28 (s, 1H), 3.35–3.30 (m, 8H), 2.95 (m, 2H) 1.10–1.06 (m, 12H), 0.99–0.98 (m, 4H), 0.63–0.59 (m, 3H). ESI-HRMS($[C_{32}H_{39}N_3O_2+H]^+$): calcd. *m*/*z* = 497.3042; found *m*/*z* = 497.3091.

Synthesis of compound RB-S.

Under N_2 atmosphere, **RB-C** (100 mg, 0.2 mmol) and Lawesson's Reagent (198 mg, 0.5 mmol) were dissolved in dry *p*-xylene (18 mL), then the mixture was stirred at 150°C for 3 h. After reaction, saturated $NaHCO_3$ aqueous solution (30 mL) was added, the mixture was extracted with ethyl acetate (3×10 mL), the brown organic layers were combined and MeOH (100 mL) was added. The solvent was evaporated under reduced pressure. The crude product was further purified using column chromatography (silica gel, DCM/ PE = 1/2) to give a white solid. Yield: 88 mg (95%). ^{1}H NMR (400 MHz, $CDCl_3$): δ = 8.18–8.16 (m, 1H), 7.50–7.49 (m, 2H), 7.11–7.09 (m, 1H), 6.40 (s, 4H), 6.30 (s, 2H), 3.49 (s, 1H), 3.38 (s, 1H), 1.59 (s, 4H), 1.22–1.19 (m, 12H), 0.72–0.69 (m, 3H).^{13}C NMR (125 MHz, $CDCl_3$): δ = 190.01, 155.47, 153.30, 149.07, 138.36, 132.12, 131.63, 128.99, 124.89, 123.12, 114.10, 113.56, 108.13, 104.20, 97.79, 96.26, 54.00, 46.20, 44.63, 44.41, 31.12, 28.97, 20.43, 13.51, 12.55. ESI-HRMS($[C_{32}H_{39}N_3OS+H]^+$): calcd. *m*/*z* = 513.2884; found *m*/*z* = 513.2889.

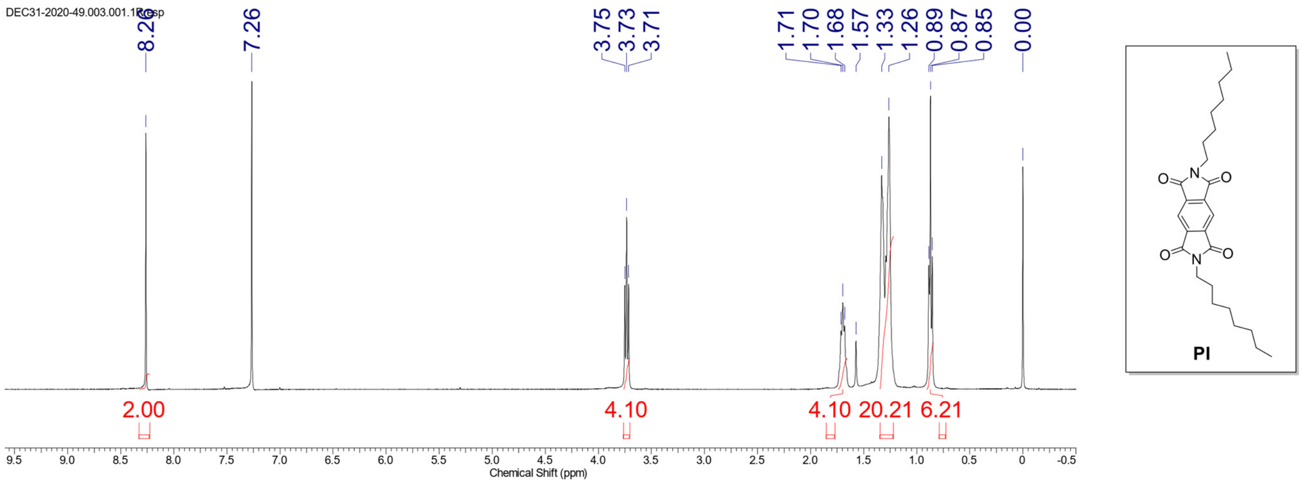

Figure S1. ^{1}H NMR spectrum of compound **PI** (600 MHz, $CDCl_3$, ppm).

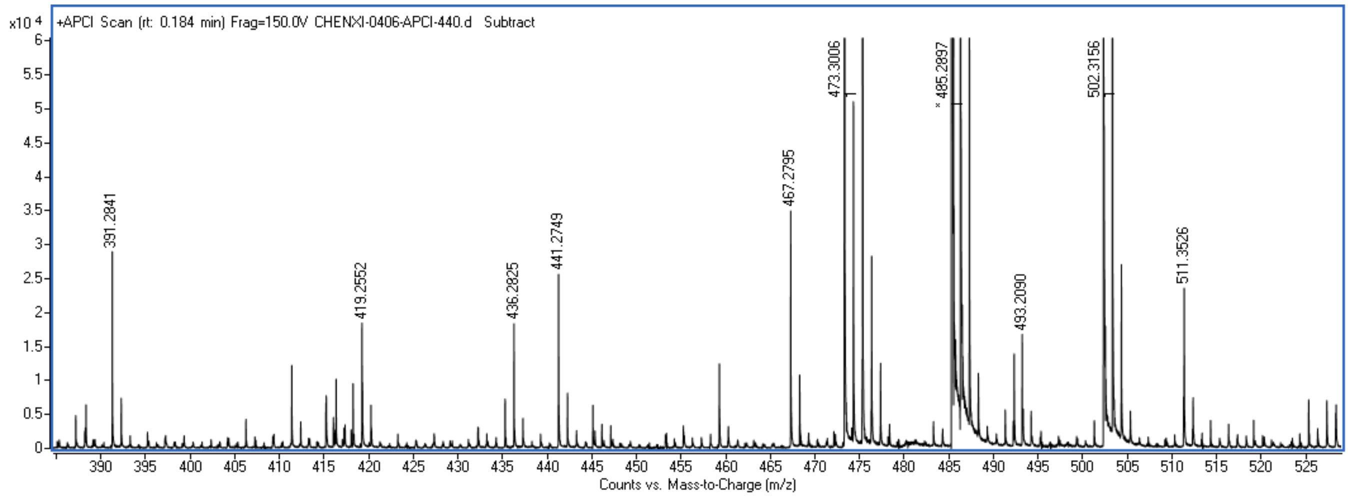

Figure S2. APCI-HRMS spectrum of compound **PI**.

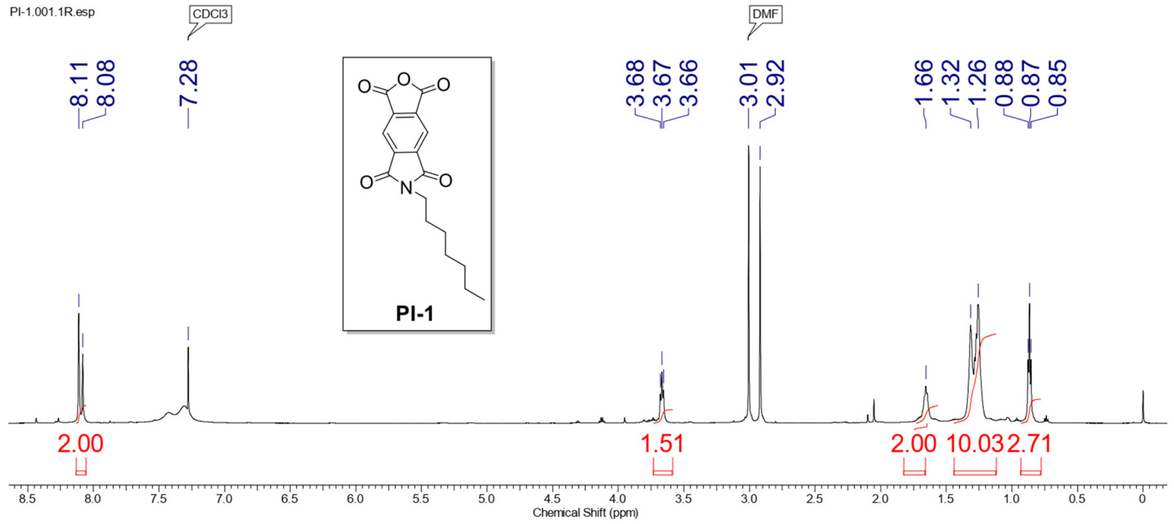

Figure S3. ^{1}H NMR spectrum of compound **PI-1** (600 MHz, $CDCl_3$, ppm).

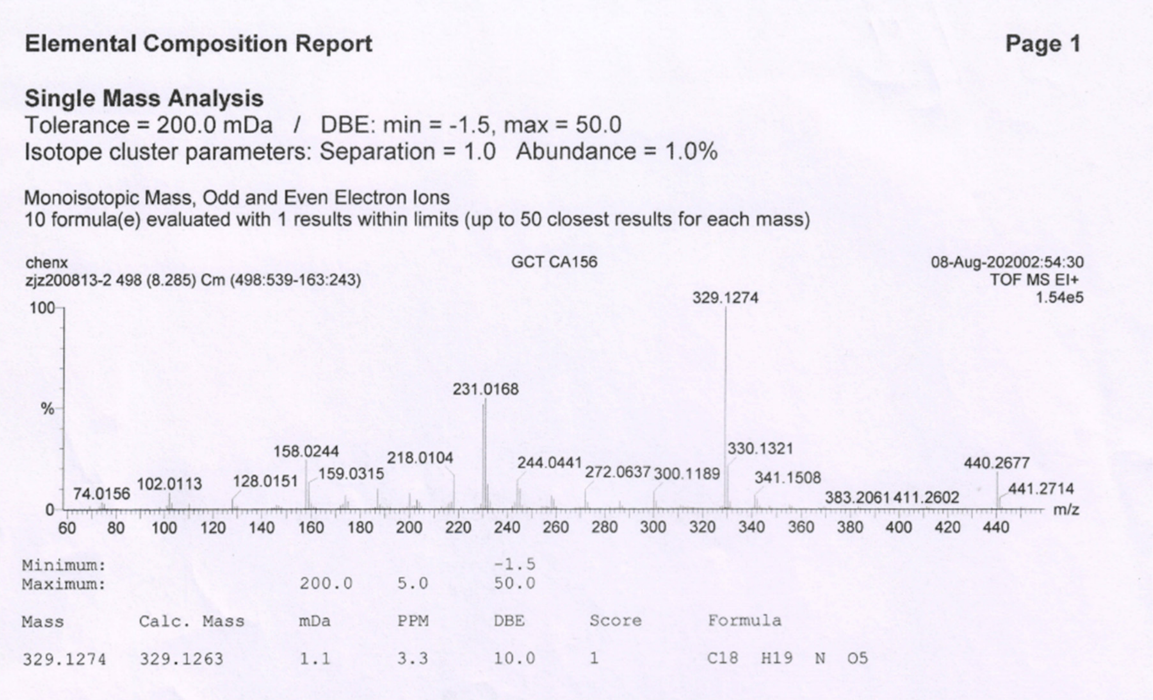

Elemental Composition Report Page 1

Single Mass Analysis
Tolerance = 200.0 mDa / DBE: min = -1.5, max = 50.0
Isotope cluster parameters: Separation = 1.0 Abundance = 1.0%

Monoisotopic Mass, Odd and Even Electron Ions
10 formula(e) evaluated with 1 results within limits (up to 50 closest results for each mass)

Minimum:				-1.5		
Maximum:		200.0	5.0	50.0		
Mass	Calc. Mass	mDa	PPM	DBE	Score	Formula
329.1274	329.1263	1.1	3.3	10.0	1	C18 H19 N O5

Figure S4. TOF-HRMS spectrum of compound **PI-1**.

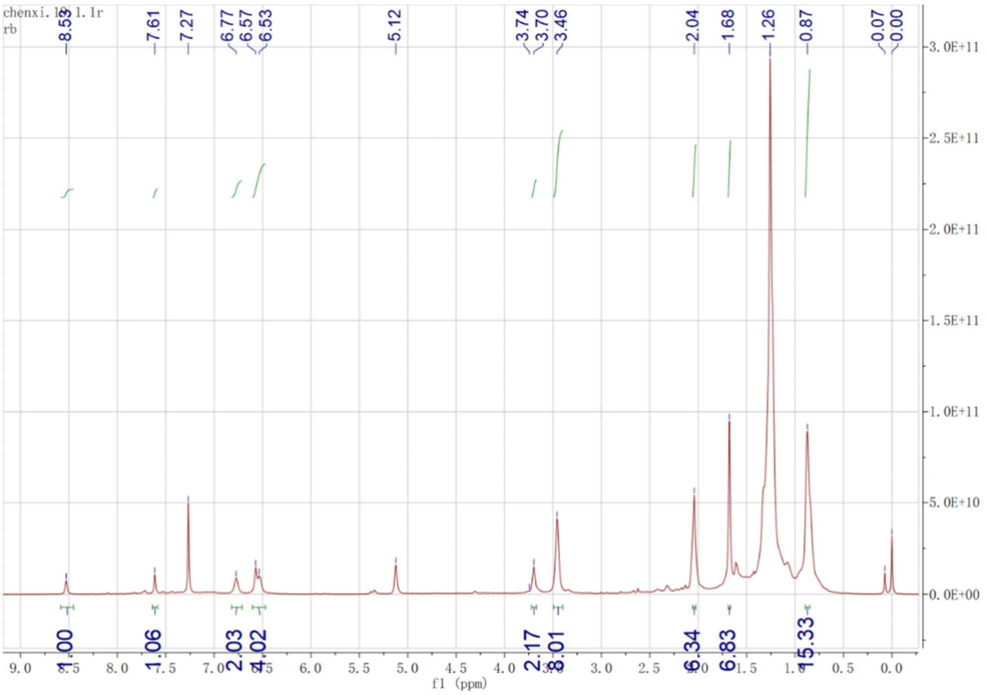

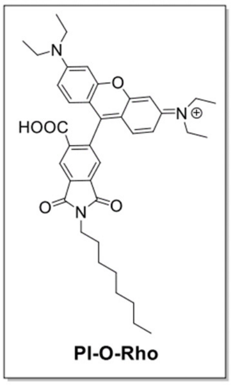

Figure S5. ^{1}H NMR spectrum of compound **PI-O-Rho** (600 MHz, $CDCl_3$, ppm)

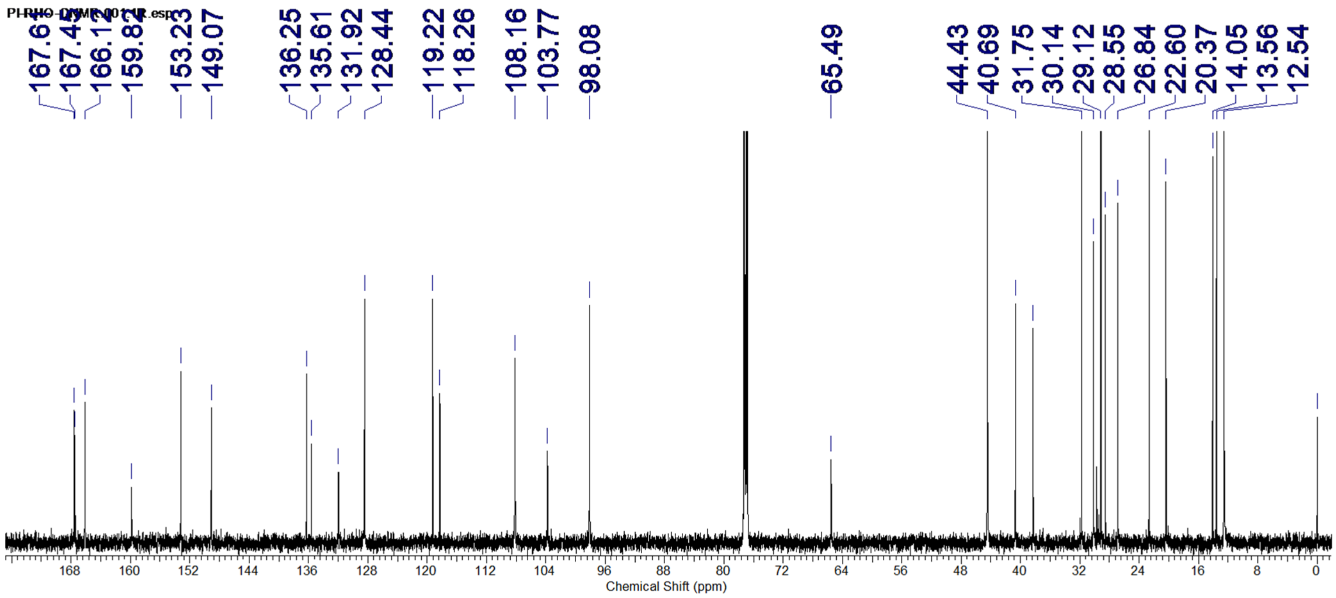

Figure S6. ^{13}C NMR spectrum of compound **PI-O-Rho** (125 MHz, $CDCl_3$, ppm).

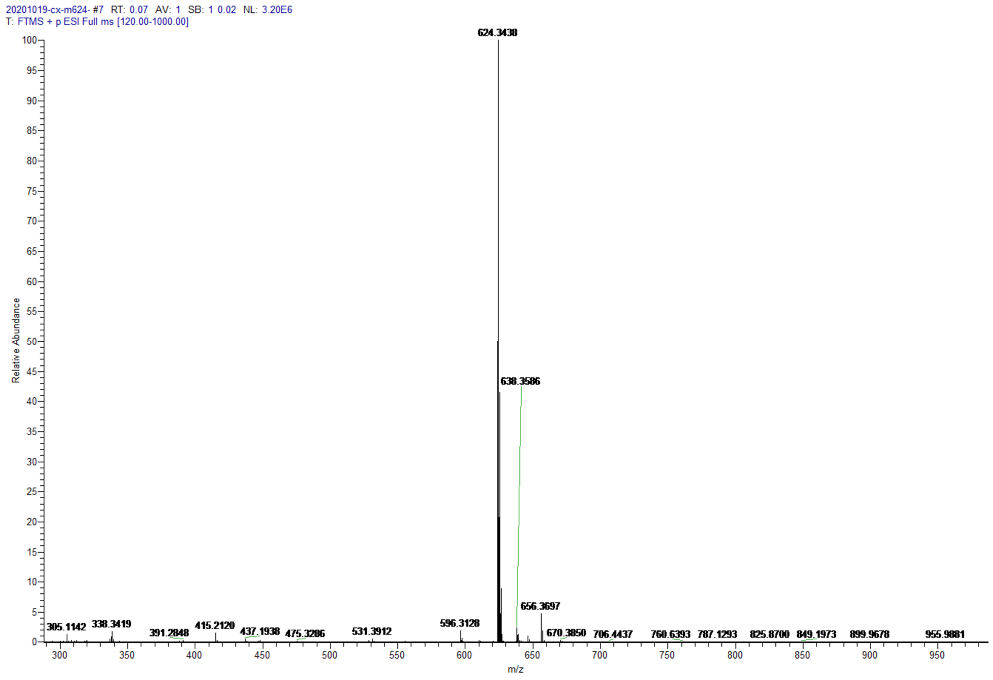

Figure S7. ESI-HRMS spectrum of compound **PI-O-Rho**.

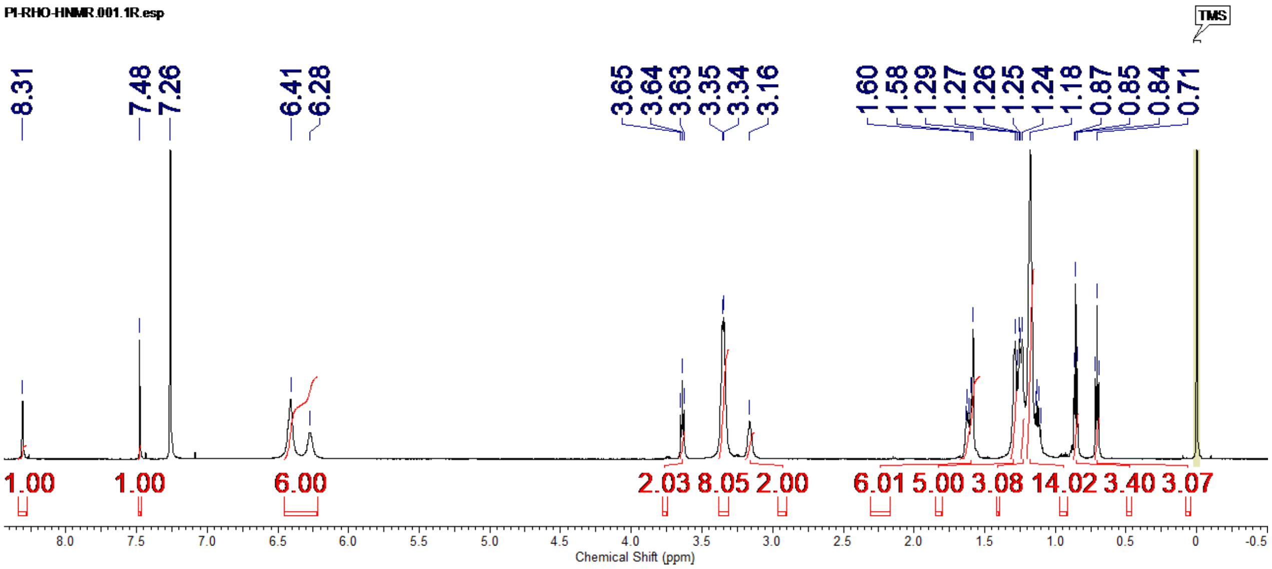

Figure S8. ^{1}H NMR spectrum of compound **PI-Rho** (400MHz, $CDCl_3$, ppm).

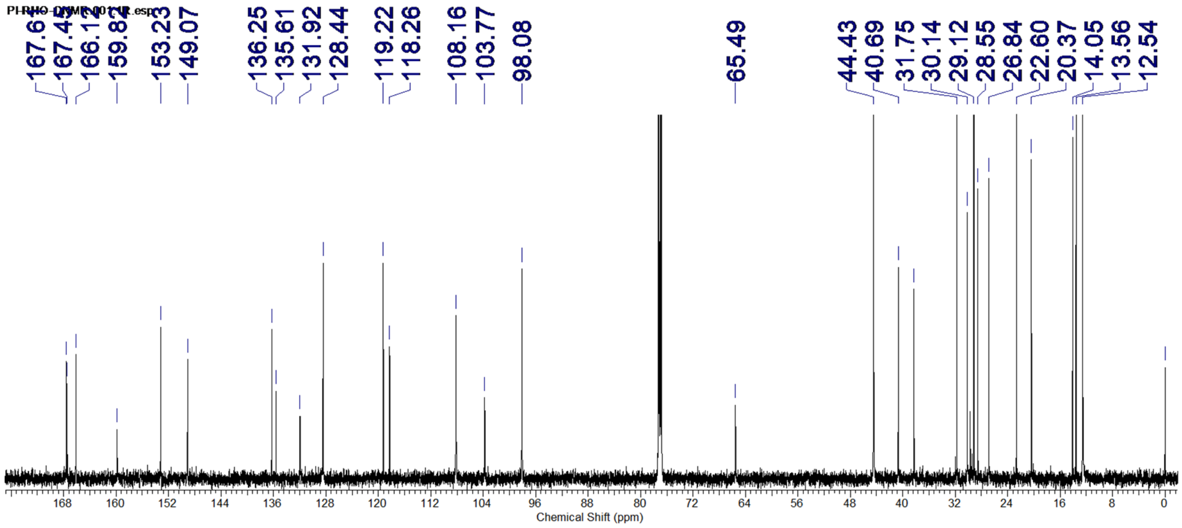

Figure S9. ^{13}C NMR spectrum of compound **PI-Rho** (125 MHz, $CDCl_3$, ppm).

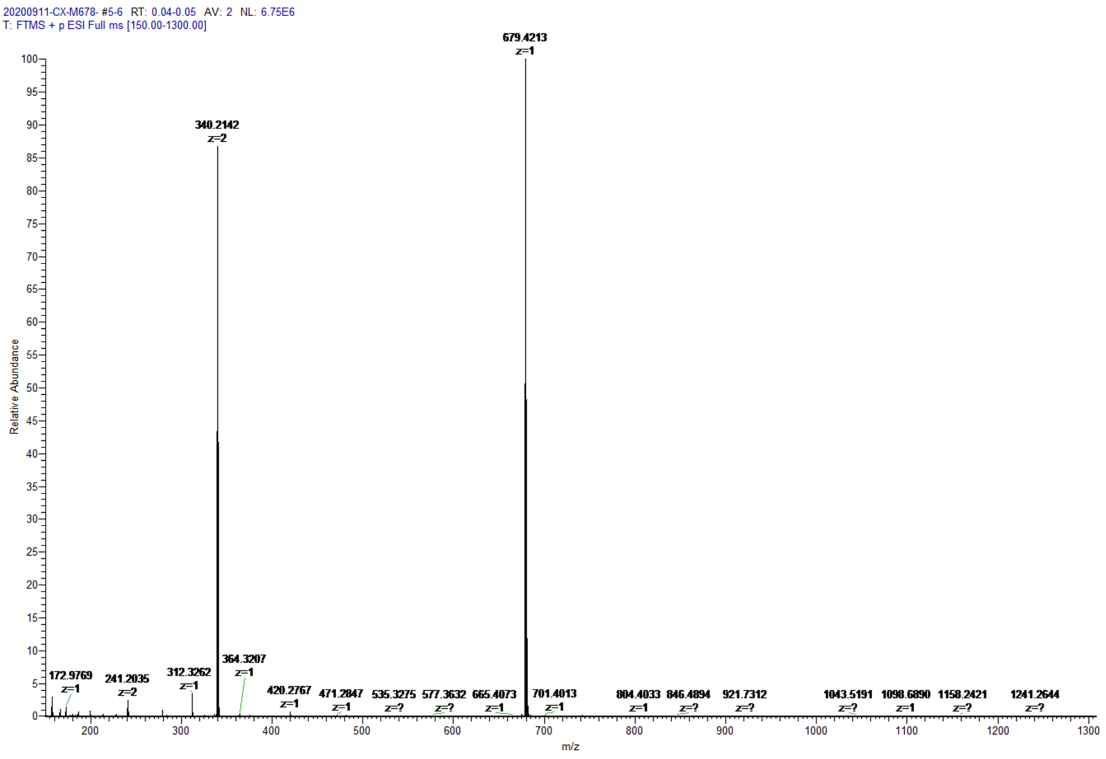

Figure S10. ESI-HRMS spectrum of compound **PI-Rho**.

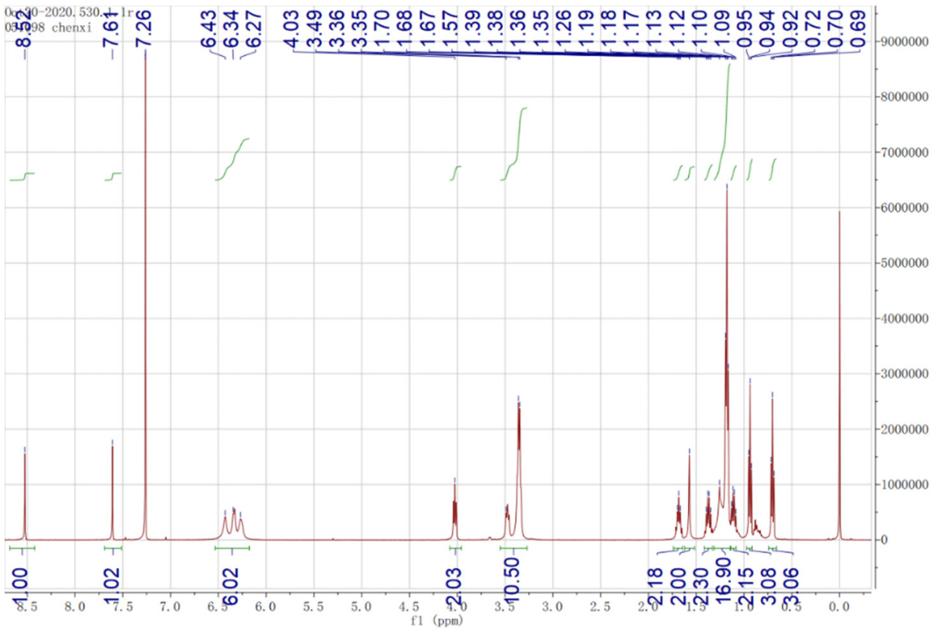

Figure S11. ^{1}H NMR spectrum of compound **PI-Rho-S** (400MHz, $CDCl_3$, ppm).

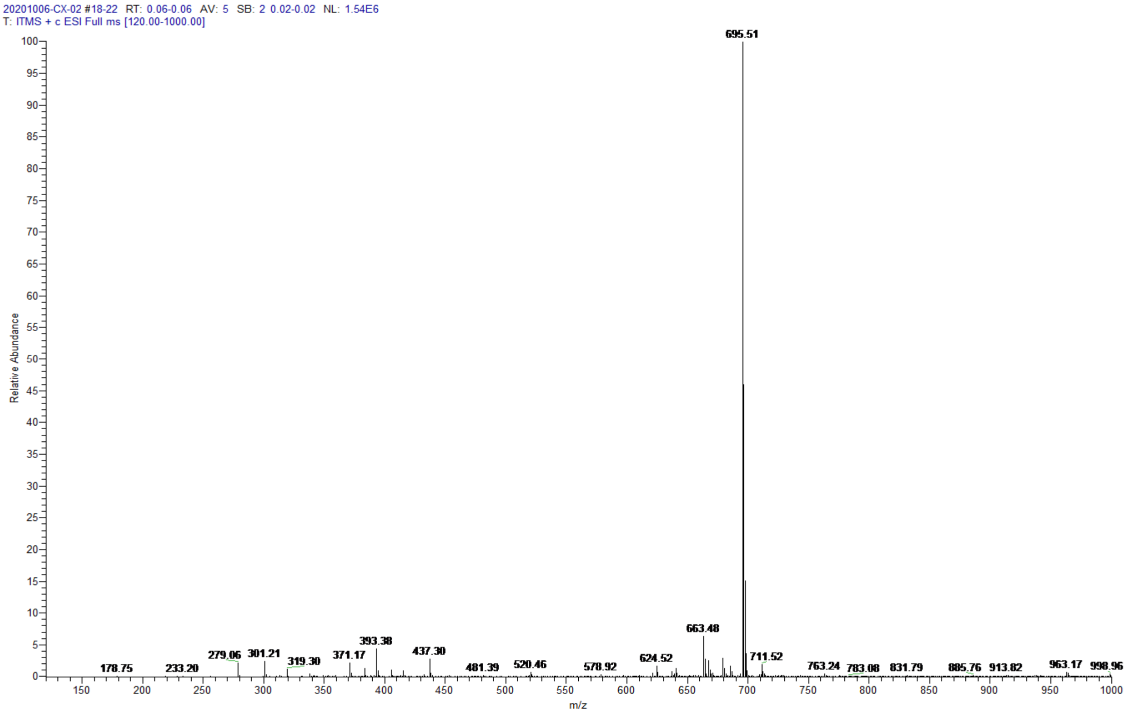

Figure S12. ESI-MS spectrum of compound **PI-Rho-S**.

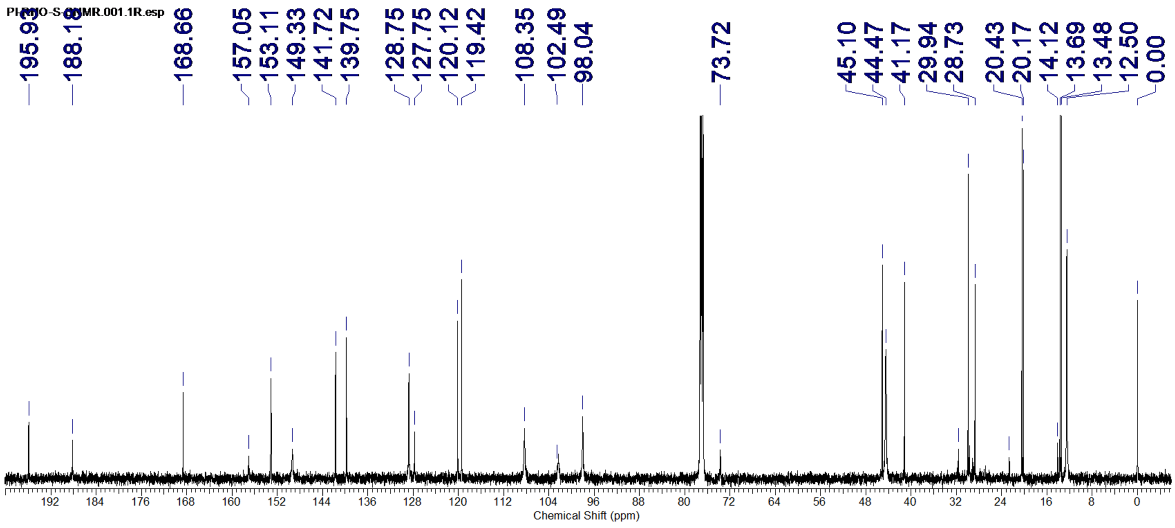

Figure S13. ^{13}C NMR spectrum of compound **PI-Rho-S** (125MHz, $CDCl_3$, ppm).

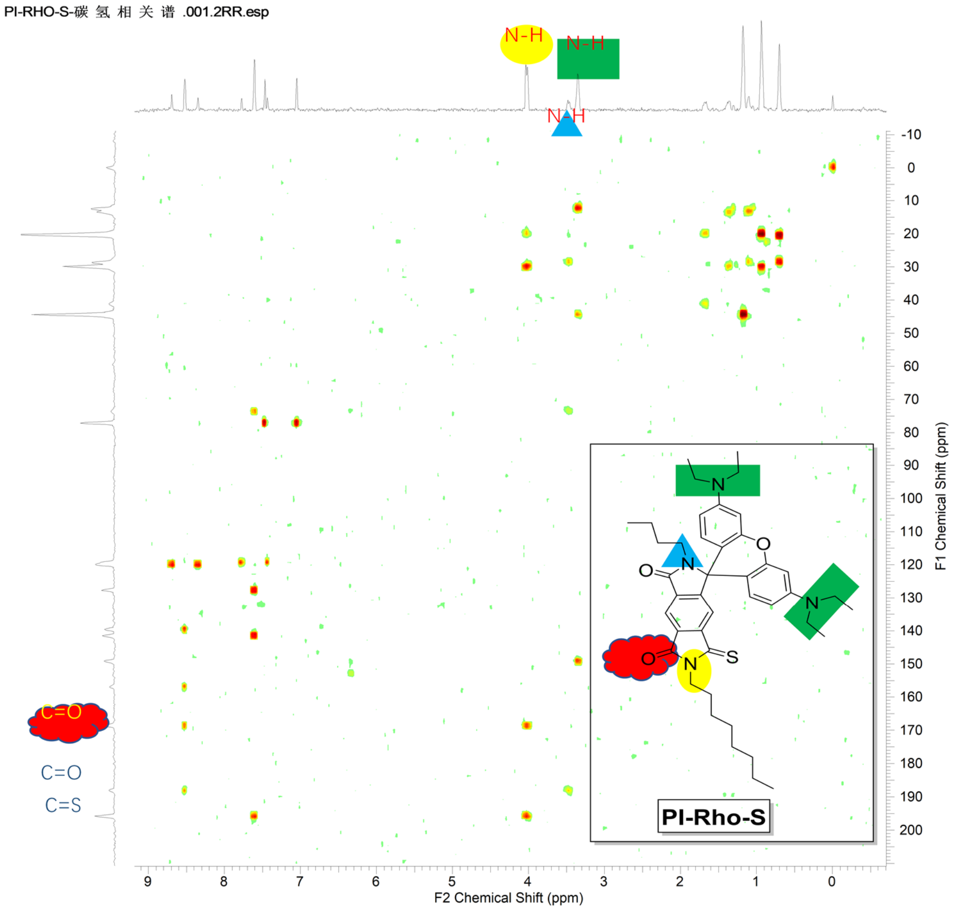

Figure S14. 2D Heteronuclear Multiple Bond Correlation (HMBC) NMR spectrum of compound **PI-Rho-S** (125MHz, $CDCl_3$, ppm).

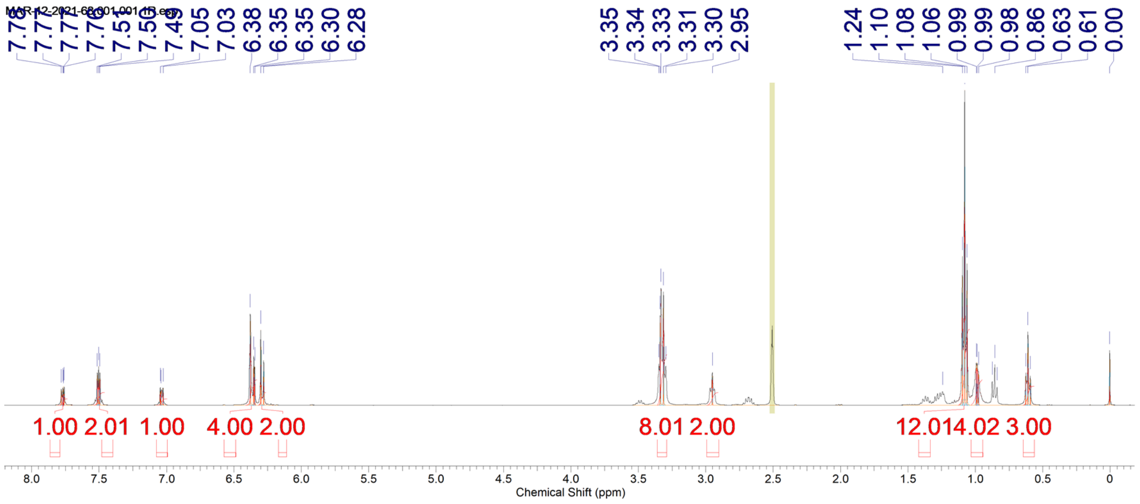

Figure S15. ^{1}H NMR spectrum of compound **RB-C** (400MHz, DMSO-d_6. ppm).

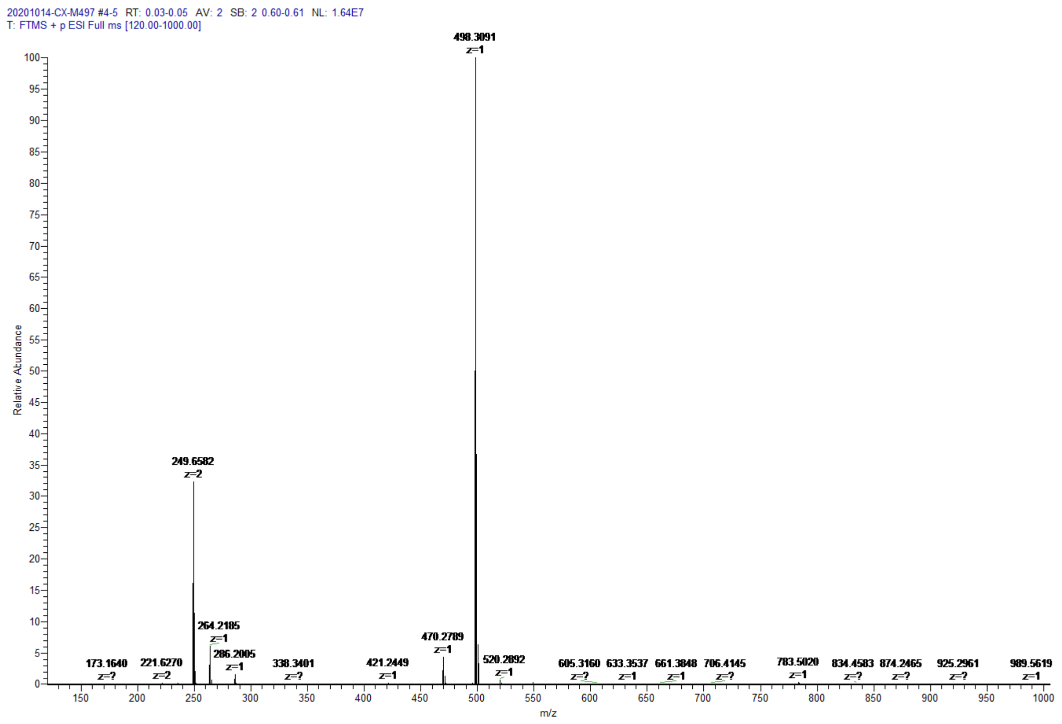

Figure S16. ESI-HRMS spectrum of compound **RB-C**.

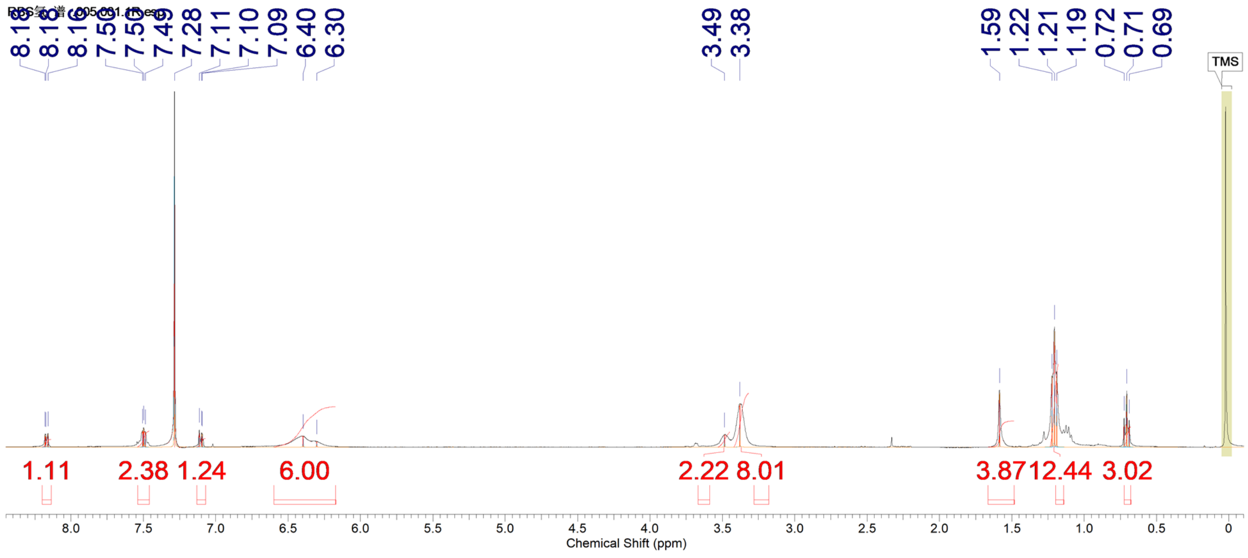

Figure S17. ^{1}H NMR spectrum of compound **RB-S** (400MHz, $CDCl_3$, ppm).

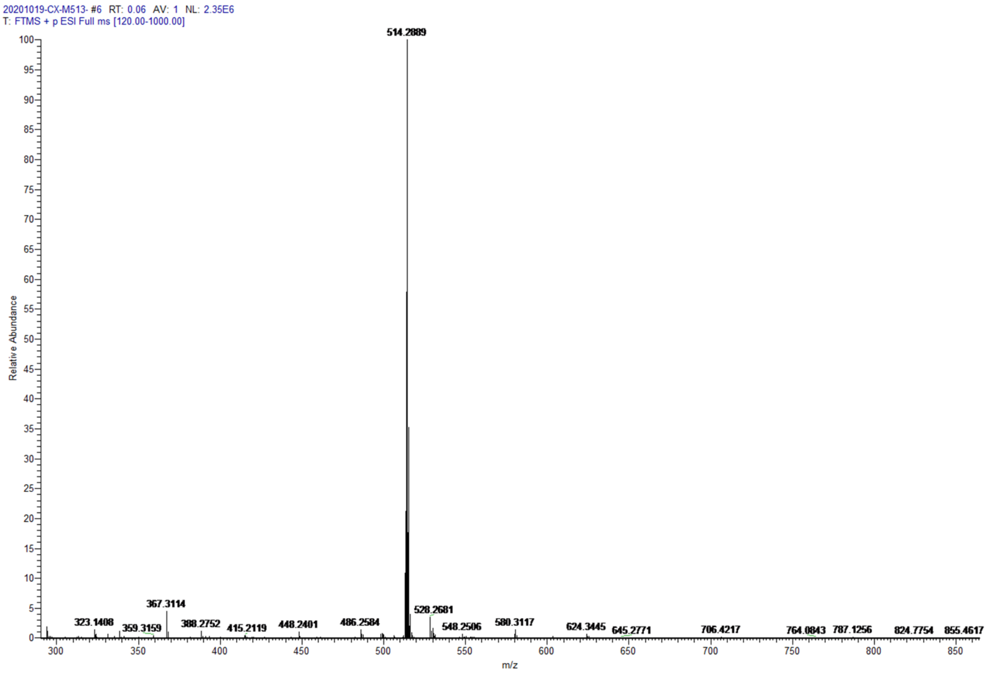

Figure S18. ESI-HRMS spectrum of compound **RB-S**.

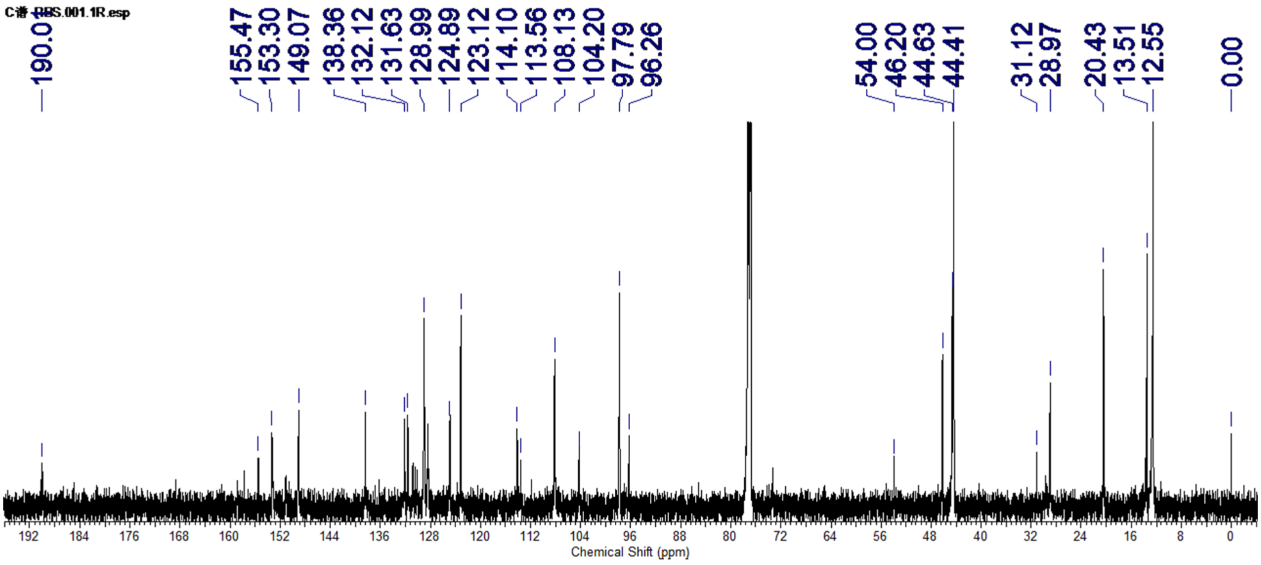

Figure S19. ^{13}C NMR spectrum of compound **RB-S** (125MHz, $CDCl_3$, ppm).

2. Crystal Data of PI-Rho

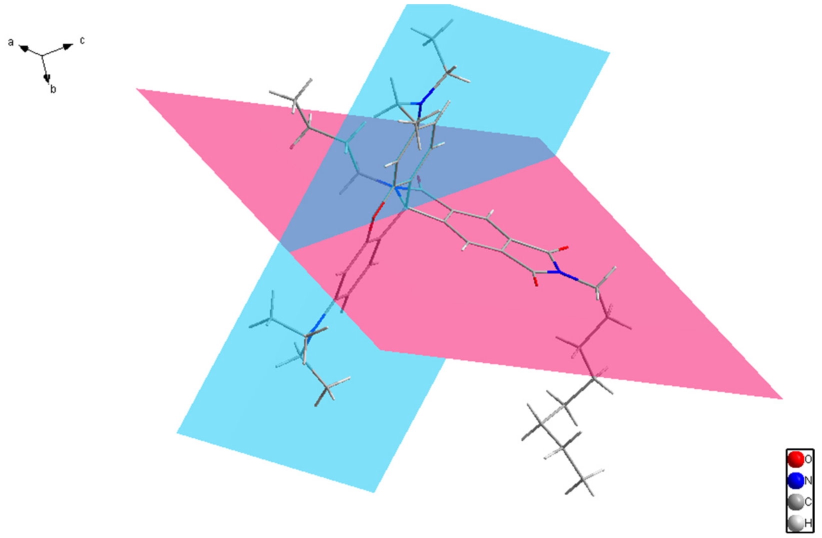

Figure S20. Dihedral angle between xanthene and the PI planes of **PI-Rho**, the two color sheets show the planes of the PI and the RB moieties.

Table S1. Single crystal X-ray diffraction data of **PI-Rho** (CCDC：2075565).

Datablock: zhl

```
Bond precision:   C-C = 0.0032 A          Wavelength=0.71073

Cell:            a=13.6758(13)    b=22.612(2)      c=12.9254(13)
                 alpha=90         beta=111.165(4)  gamma=90
Temperature:     150 K

                 Calculated                 Reported
Volume           3727.4(6)                  3727.4(6)
Space group      P 21/c                     P 1 21/c 1
Hall group       -P 2ybc                    -P 2ybc
Moiety formula   C42 H54 N4 O4              C42 H54 N4 O4
Sum formula      C42 H54 N4 O4              C42 H54 N4 O4
Mr               678.89                     678.89
Dx,g cm-3        1.210                      1.210
Z                4                          4
Mu (mm-1)        0.078                      0.078
F000             1464.0                     1464.0
F000'            1464.58
h,k,lmax         17,29,16                   17,29,16
Nref             8547                       8533
Tmin,Tmax        0.985,0.988                0.524,0.746
Tmin'            0.985

Correction method= # Reported T Limits: Tmin=0.524 Tmax=0.746
AbsCorr = MULTI-SCAN

Data completeness= 0.998          Theta(max)= 27.478

R(reflections)= 0.0590( 4735)     wR2(reflections)= 0.1440( 8533)

S = 1.013                 Npar= 457
```

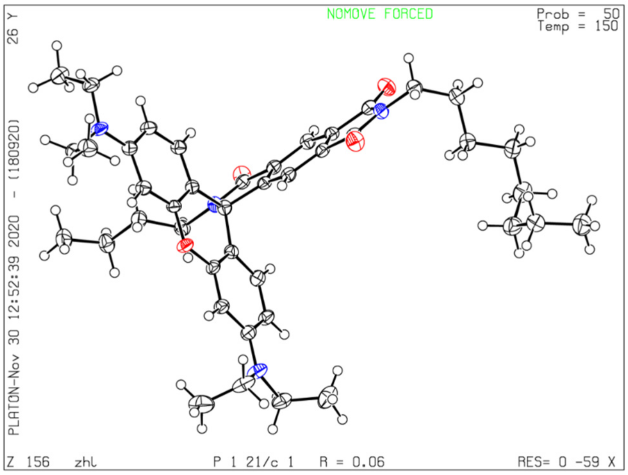

Figure S21. Molecular structure of **PI-Rho** determined by Single crystal X-ray diffraction (CCDC：2075565). Thermal ellipsoids are set at 50% probability.

3. UV–Vis Absorption and Fluorescence Emission Spectra.

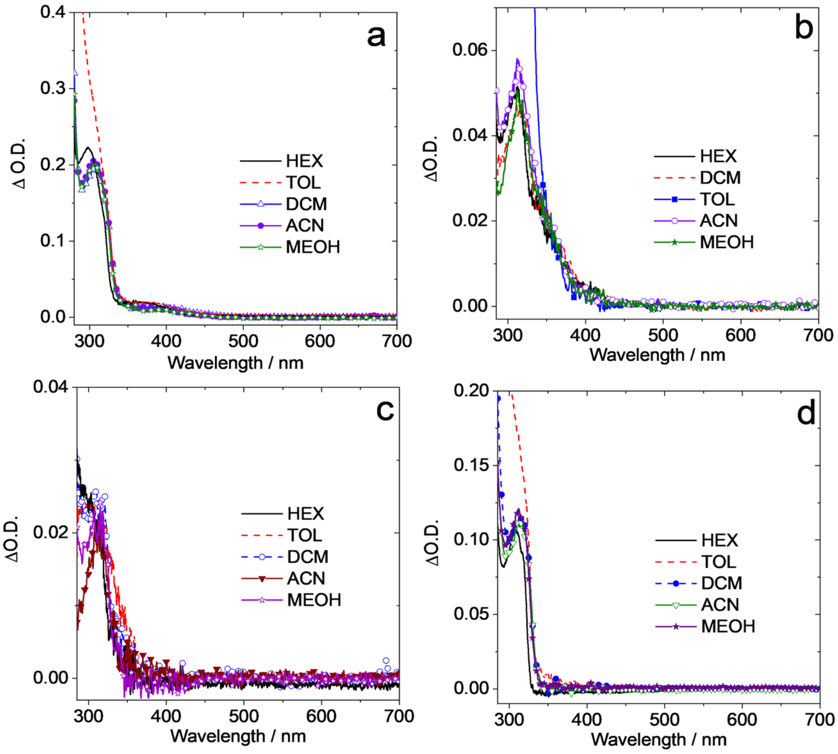

Figure S22. UV−vis absorption spectra of the compounds (a) **PI-Rho** and (b) **PI-Rho-S** (c) **PI** (d) **RB-C** in different solvents. $c = 1.0 \times 10^{-5}$ M. 20 °C.

We used Hush eq S1 to calculate the electronic coupling matrix element (H_{AB}) in neutral donor/acceptor systems.

$$H_{AB}(\mathrm{cm}^{-1}) = (\frac{2.06\times10^{-2}}{R})(\varepsilon_{\max}^{\mathrm{CT}}\nu_{\max}^{-\mathrm{CT}}\Delta\nu_{1/2}^{-\mathrm{CT}})^{1/2} \quad (1)$$

where R is the separation between the center of electron donor and acceptor, in Å; $\varepsilon_{\max}^{\mathrm{CT}}$ is the molar absorption coefficient at the maximum of the CT absorption band, in [$M^{-1}cm^{-1}$]; $\nu_{\max}^{-\mathrm{CT}}$ is the absorption maximum of the CT absorption band in wavenumber scale, in cm^{-1}; $\Delta\nu_{1/2}^{-\mathrm{CT}}$ is the full width of the band. The H_{AB} were calculated according to eq S1 and the data are presented in Table S2.

Table S2. The H_{AB} of the compounds **PI-Rho** and **PI-Rho-S**.

Compound	*Solvent*	R[a]	$\varepsilon_{\max}^{\mathrm{CT}}$[b]	$\nu_{\max}^{-\mathrm{CT}}$ (cm^{-1})	$\Delta\nu_{1/2}^{-\mathrm{CT}}$ (cm^{-1})	H_{AB} (cm^{-1})
**PI-Rho**	HEX	8	2000	27322	5384	1397
	TOL	8	2183	26882	5684	1487
	DCM	8	1391	26525	6290	1240
	ACN	8	1431	26455	5517	1177
	MeOH	8	1100	26178	5962	1067
**PI-Rho-S**	HEX	8	2490	30487	4377	1484
	TOL	8	—[c]	—[c]	—[c]	—[c]
	DCM	8	—[c]	—[c]	—[c]	—[c]
	ACN	8	—[c]	—[c]	—[c]	—[c]
	MeOH	8	—[c]	—[c]	—[c]	—[c]

[a] the separation between the center of electron donor and acceptor, in Å; [b] in [$M^{-1}cm^{-1}$]; [c] Not applicable.

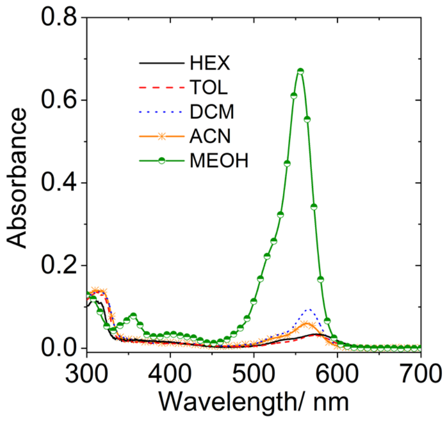

Figure S23. UV−vis absorption spectra of the **PI-O-Rho** in different solvents. $c = 1.0 \times 10^{-5}$ M. 20 °C.

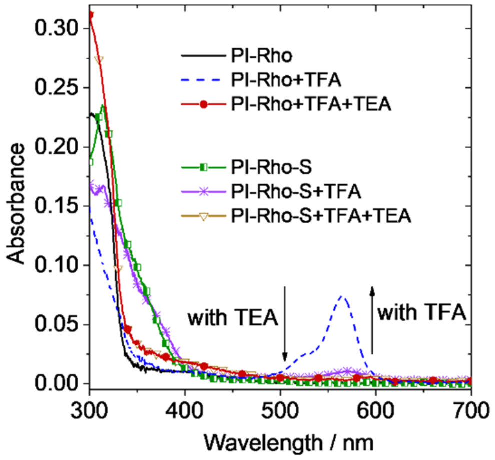

Figure S24. UV−vis absorption spectra of the compound **PI-Rho** and **PI-Rho-S** with addition of TFA (5 mol/L,150 μL) in CH_3OH or TEA (50μL, 150μL respectively) for switching purpose. c = 1.0 × 10^{-5} M. 20 °C.

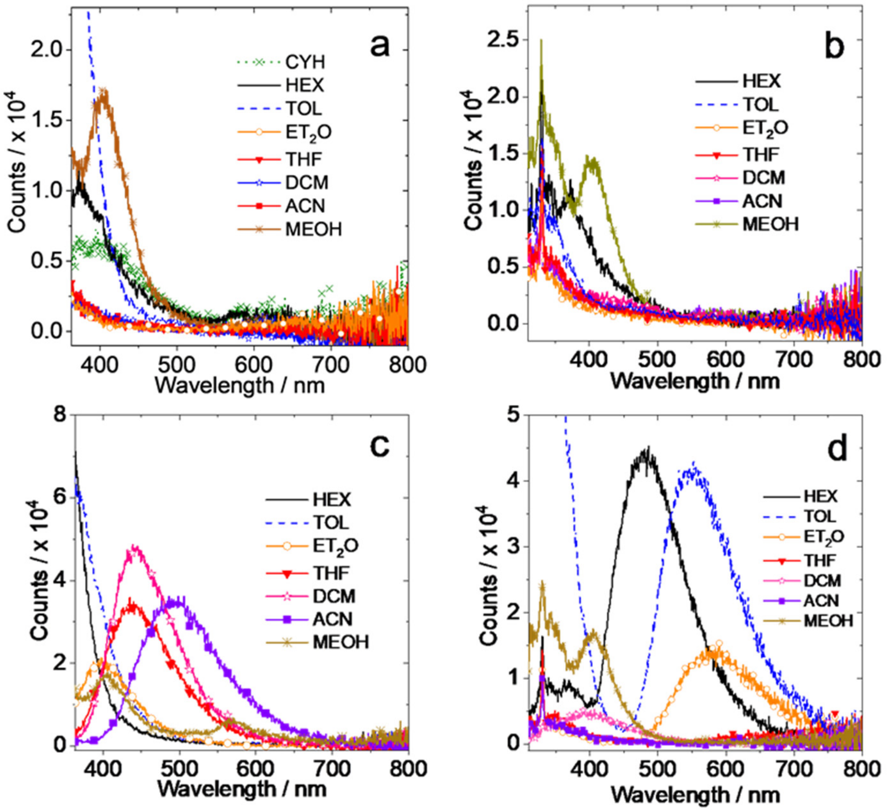

Figure S25. Fluorescence emission spectra of (a) **PI-Rho-S** (b) **PI** (c) **RB-C** (d) **PI-Rho** (optically matched solutions were used, A = 0.106, λ_{ex} = 300 nm). The peak at 331 nm in (b) and (d) represents the Raman scattering peak of the solvent.

4. Fluorescence lifetime and phosphorescence lifetime.

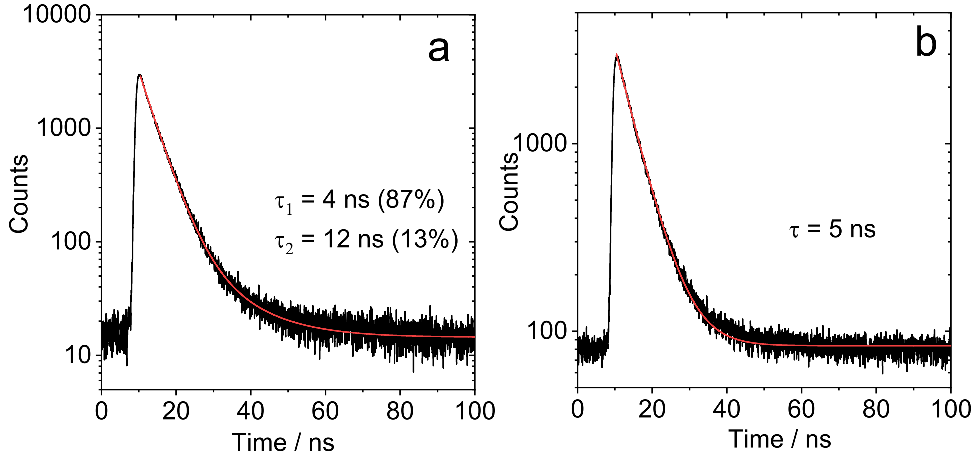

Figure S26. Fluorescence decay traces of **PI-Rho** in *n*-hexane at 460 nm (a) in air (b) in N_2 excited with a picosecond pulsed laser (λ_{ex} = 340 nm). The monoexponential fittings are also presented. $c = 1.0 \times 10^{-5}$ M, 20 °C.

The Fluorescence lifetime were measured in different atmosphere (Figure S26). The average fluorescence lifetime of **PI-Rho** in *n*-hexane at 460 nm at air atmosphere is 5 ns, which is similar to N_2 atmosphere.

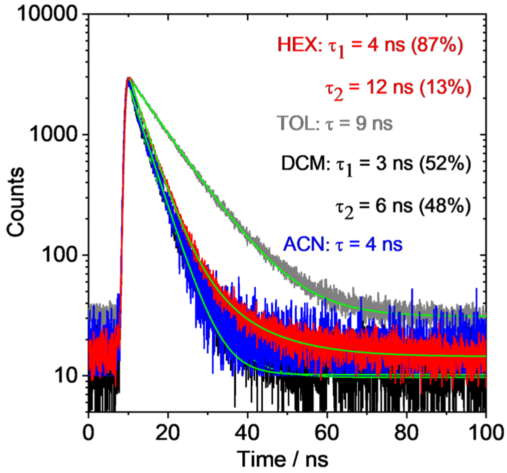

Figure S27. Fluorescence decay traces of **PI-Rho** in different solvents. (λ_{em} = 460 nm in HEX, λ_{em} = 535 nm in TOL; λ_{em} = 600 nm in DCM; λ_{em} = 600 nm in ACN), excited with a picosecond pulsed laser (λ_{ex} = 340 nm). *c* = 1.0 × 10^{-5} M, 20 °C.

Fluorescence lifetime was measured in different solutions. The fluorescence lifetime of **PI-Rho** in TOL is 9 ns, which is similar to that of PI (7 ns). In HEX and DCM solution, however, the fluorescence decay of **PI-Rho** is with a double exponential fit (at 460 nm), which indicates that two processes exist, one with a short lifetime of 4 ns (population: 87%) and 3 ns (population: 52%), the other with a long-life path attenuation of 12 ns (population: 13%) and 6 ns (population: 48%). The longer lifetime component shows the non-radiative transition of the emissive state of **PI-Rho** in HEX and DCM.

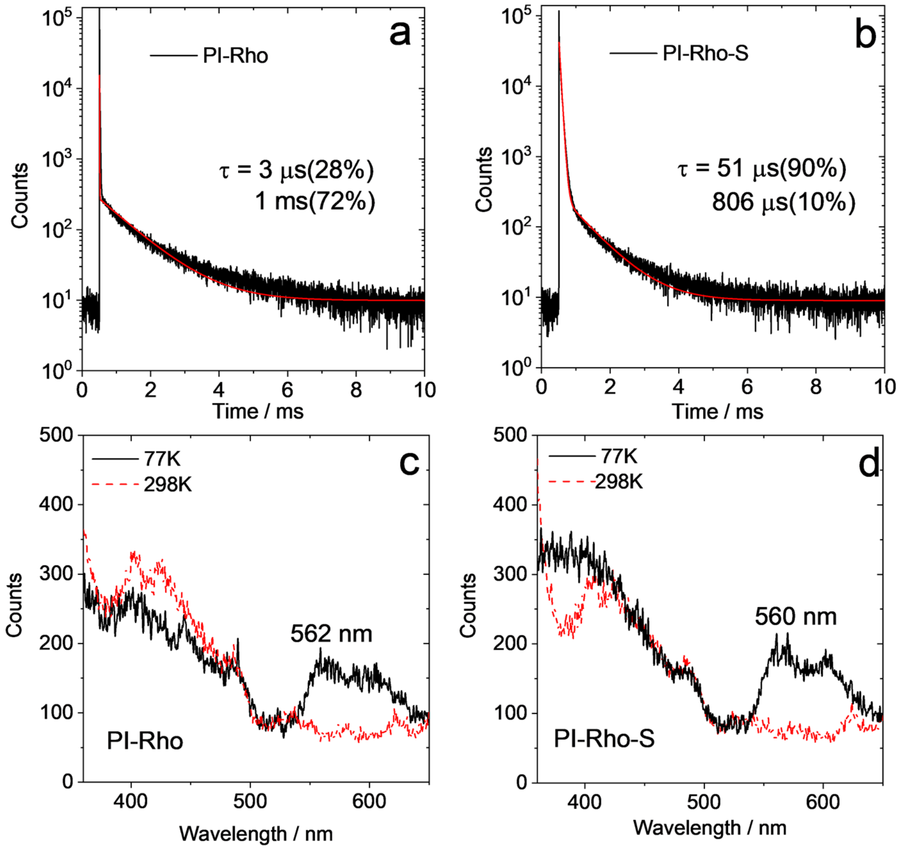

Figure S28. Phosphorescence decay traces of (a) **PI-Rho** at 562 nm, (b) **PI-Rho-S** at 560 nm. Phosphorescence spectra of the compounds (c) **PI-Rho** and (d) **PI-Rho-S**. (λ_{ex} = 340 nm) in aerated MeTHF, excited with a microsecond lamp (λ_{ex} = 340 nm). c = 1.0 × 10^{-5} M, 77K.

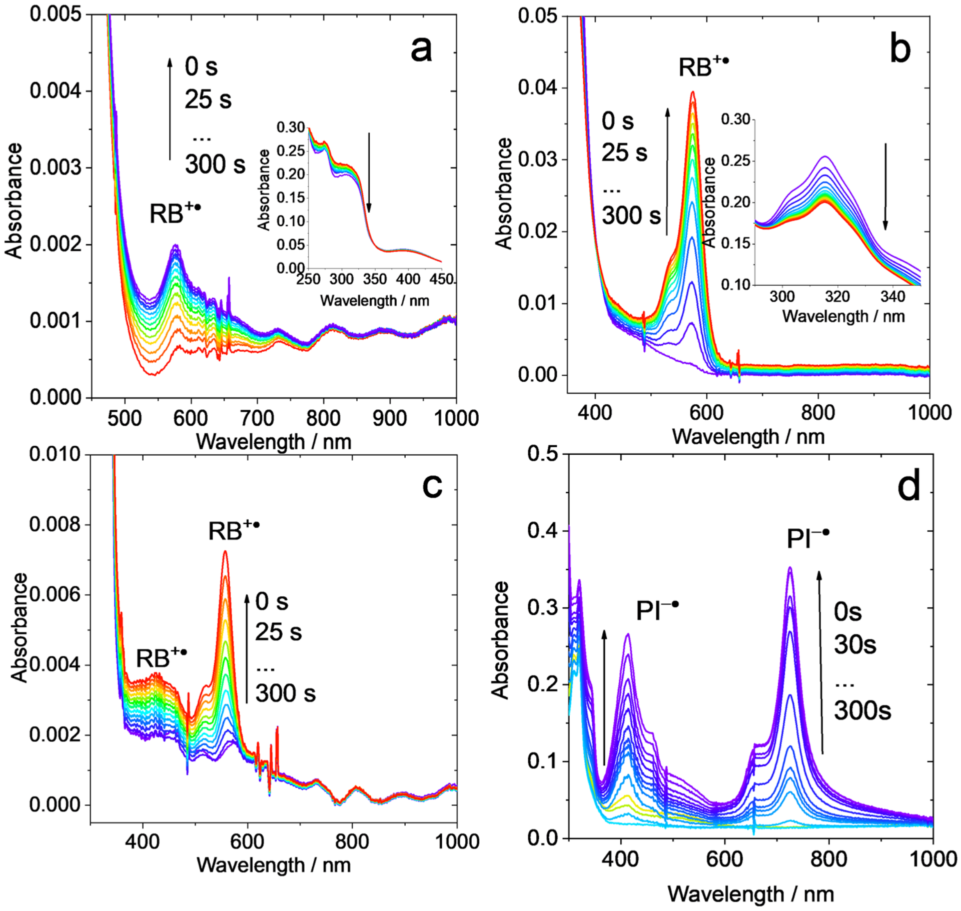

Figure S29. Spectroelectrochemistry spectra of (a) **PI-Rho** cation, upon reduction under 0.97 V (vs. Ag/$AgNO_3$), (b) **PI-Rho-S** cation upon reduction under 0.85 V (vs. Ag/$AgNO_3$), (c) **RB-C** cation upon reduction under 0.72 V (vs. Ag/$AgNO_3$) (d) **PI** anion upon reduction under −1.27 V (Ag/$AgNO_3$) in deaerated dichloromethane containing 0.10 mol/L $Bu_4N[PF_6]$ as supporting electrolyte, Ag/$AgNO_3$ as reference electrode. $c = 1.0 \times 10^{-5}$ M, 20 °C

5. Nanosecond Time-resolved Transient Absorption Spectra.

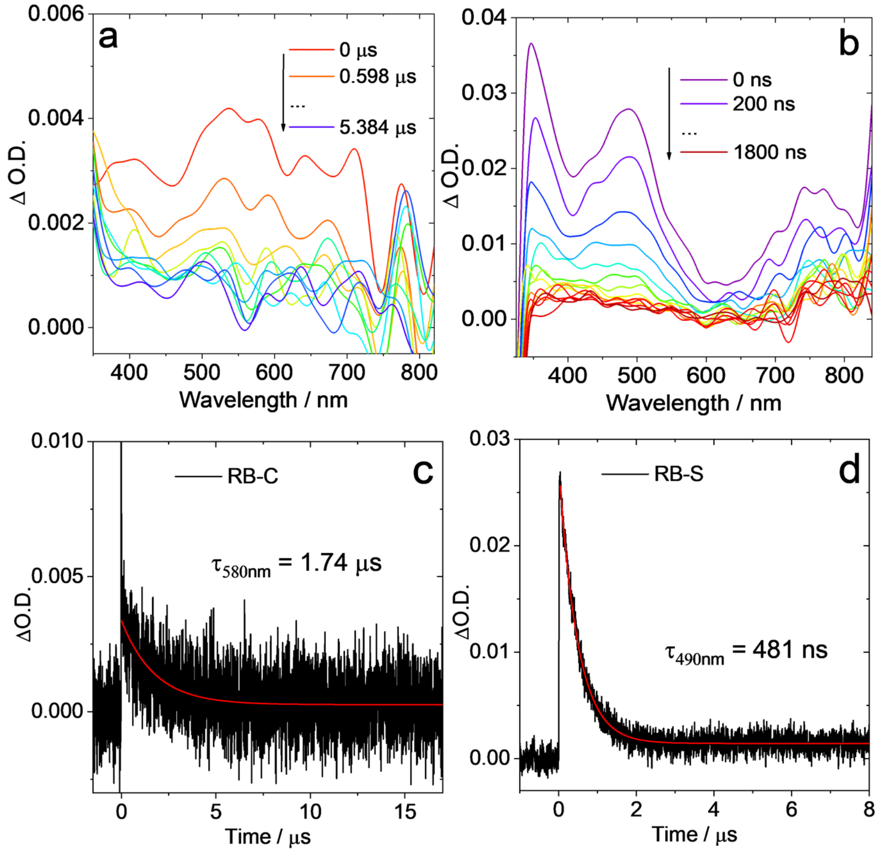

Figure S30. Nanosecond transient absorption spectra of (a) **RB-C** (b) **RB-S** in deaerated toluene, Decay kinetics of (c) **RB-C** at 580 nm, (d) **RB-S** at 580 nm. λ_{ex} = 355 nm, c = 1.0 × 10^{-4} M, 20 °C.

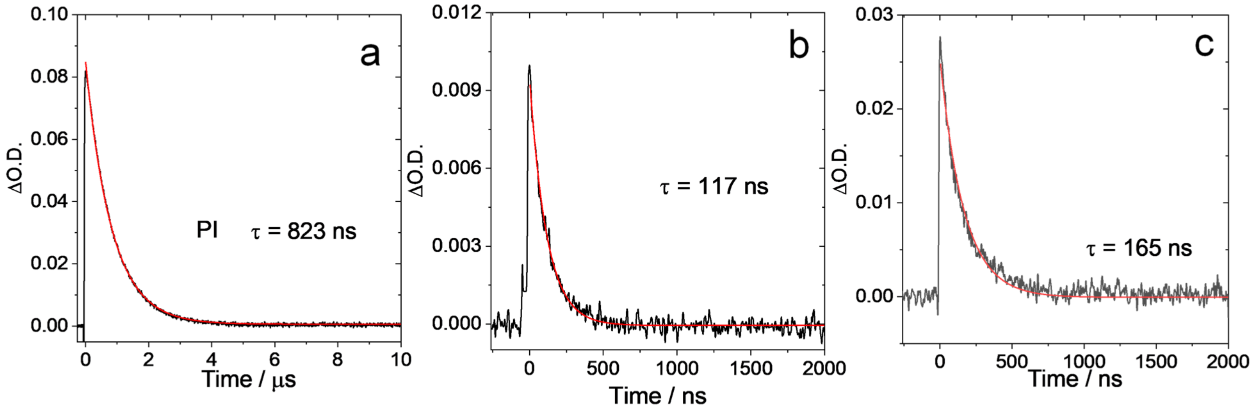

Figure S31. Decay kinetics of (a) **PI** at 450 nm, (b) **PI-Rho** at 430 nm, (c) **PI-Rho-S** at 430 nm in aerated toluene. λ_{ex} = 355 nm, c = 1.0 × 10^{-4} M, 20 °C.

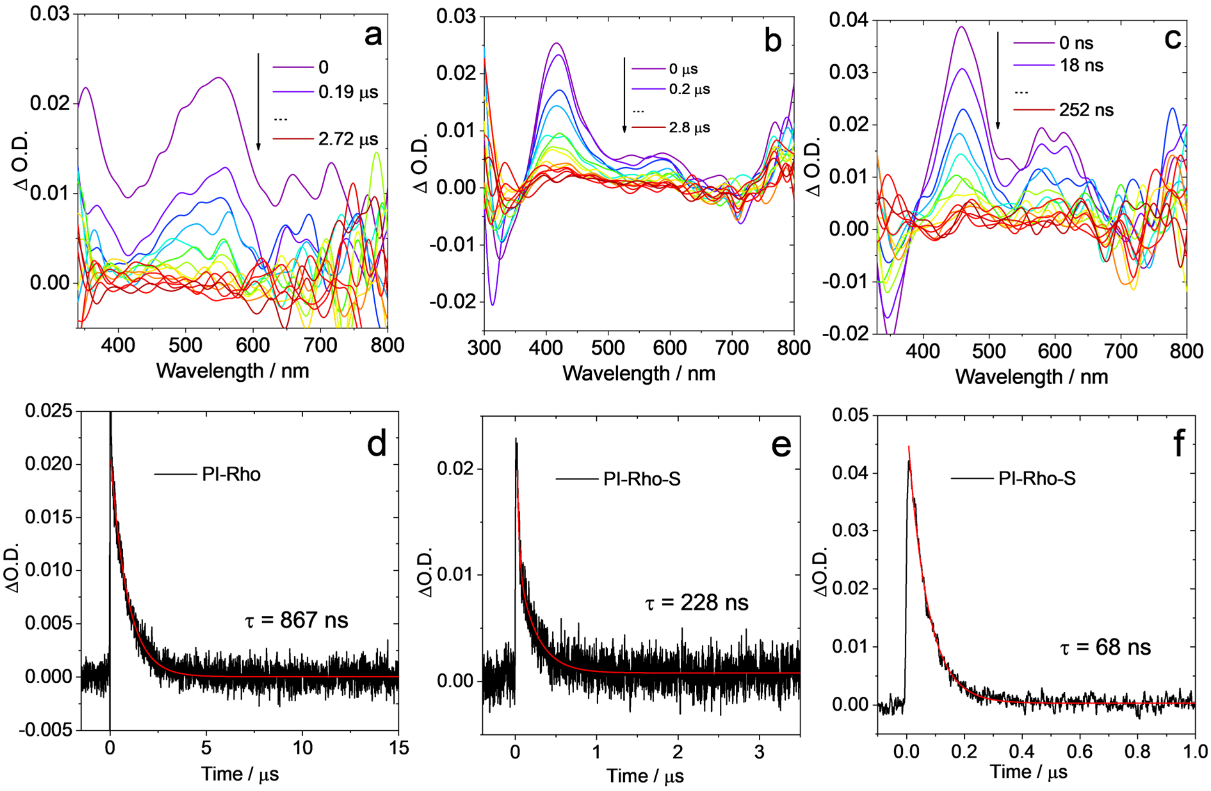

Figure S32. Nanosecond transient absorption spectra of (a) **PI-Rho** in deaerated hexane. (b) **PI-Rho-S** in deaerated hexane. (c) **PI-Rho-S** in deaerated acetonitrile. Decay kinetics of (d) **PI-Rho** at 430 nm in deaerated hexane. (e) **PI-Rho-S** at 420 nm in deaerated hexane. (f) **PI-Rho-S** at 460 nm in deaerated acetonitrile, λ_{ex} = 355 nm, c = 1.0 × 10^{-4} M, 20 °C.

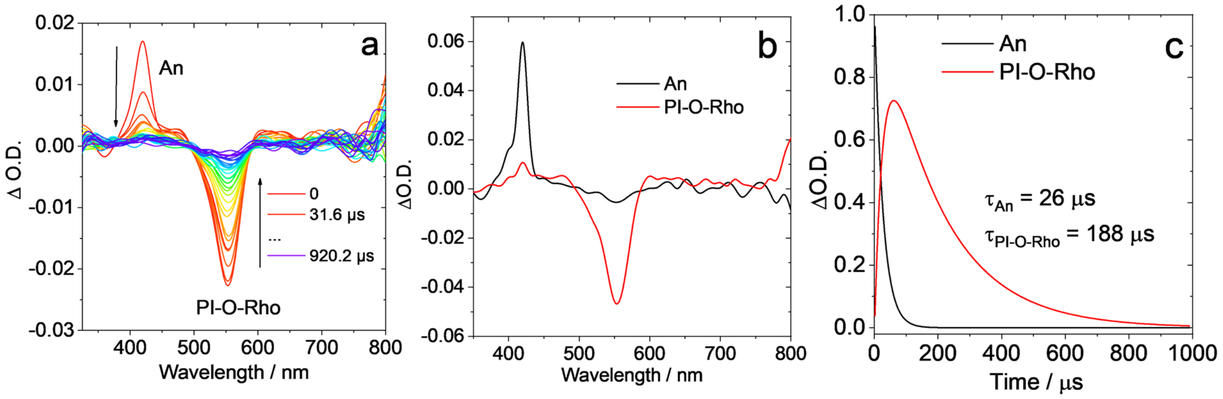

Figure S33. Intermolecular triplet−triplet energy transfer (TTET) with Anthracene (An) (c = 1 × 10^{-5} M) as the triplet photosensitizer and **PI-O-Rho** (c = 1 ×10^{-5} M) as the triplet energy acceptor in toluene, λ_{ex} = 355 nm. (a) Nanosecond transient absorption contour map spectra of the mixture of An and **PI-O-Rho**, (b) Evolutions-associated difference spectra (EADS) of nanosecond transient absorption spectra of TTET obtained by global fitting (sequential model), and (c) Decay trace at 560 nm. 25°C.

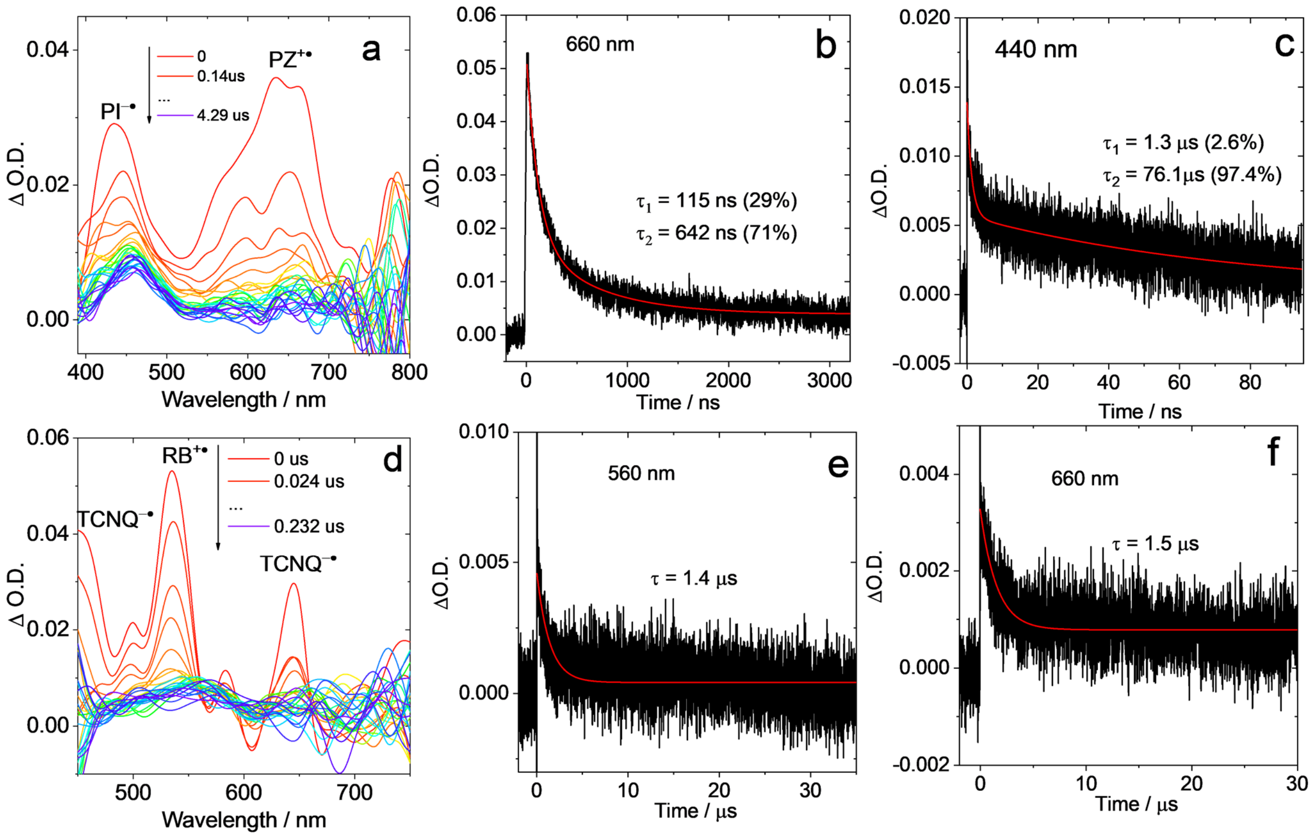

Figure S34. Nanosecond transient absorption spectra of (a) quenching of the radical anion of the CT state of **PI-Rho**, c = 1 ×10^{-4} M (PZ as electron donor, c = 3 ×10^{-4} M), (b) decay trace of radical cation at 660 nm, (c) decay trace of radical anion at 440 nm and (d) quenching of the radical cation of **PI-Rho**, c = 1 ×10^{-4} M (TCNQ as electron acceptor, c = 1 ×10^{-4} M), (e) decay trace of radical cation at 560 nm, (f) decay trace of radical anion at 660 nm.

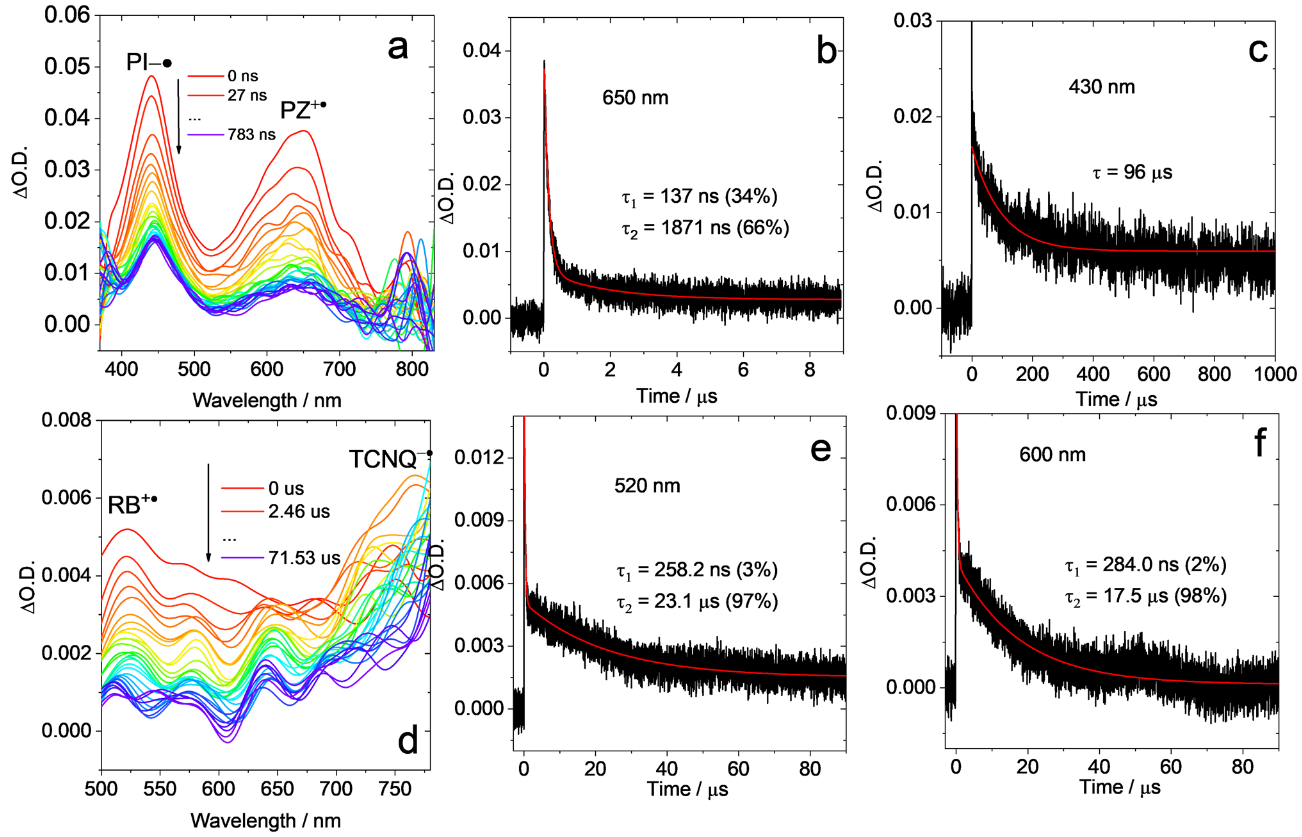

Figure S35. Nanosecond transient absorption spectra of (a) quenching of the radical anion of the CT state of **PI-Rho-S**, *c* = 1 ×10^{-4} M (PZ as electron donor, *c* = 3 ×10^{-4} M), (b) decay trace of radical cation at 650 nm, (c) decay trace of radical anion at 430 nm and (d) quenching of the radical cation of **PI-Rho-S**, *c* = 1 ×10^{-4} M (TCNQ as electron acceptor, *c* = 1 ×10^{-4} M), (e) decay trace of radical cation at 520 nm, (f) decay trace of radical anion at 600 nm.

6. Femtosecond Time-resolved Transient Absorption Spectra.

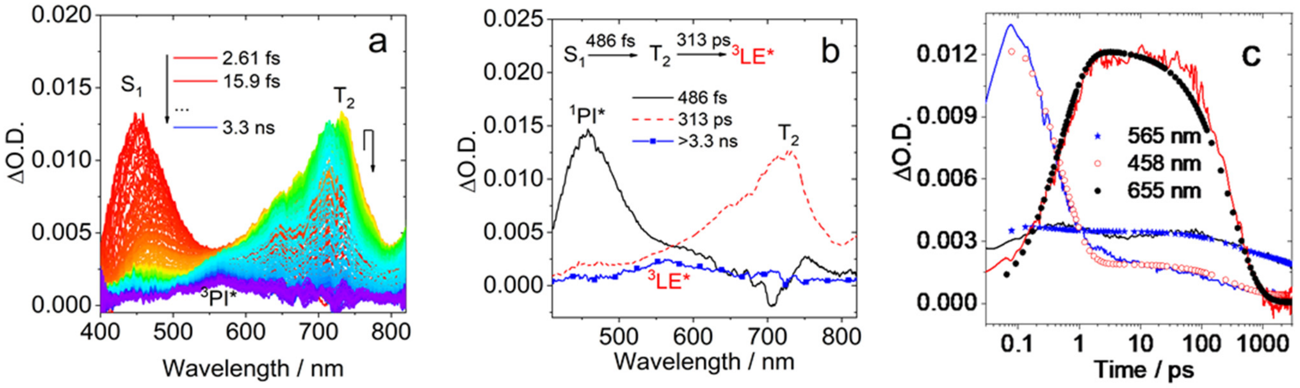

Figure S36. (a) Femtosecond transient absorption spectra of (a) **PI**, color code goes from red to blue covering the time interval from 2.6 fs to 3.3 ns. SADS of (b) **PI** obtained from global analysis. (c) Decay kinetics of **PI** at 458 nm, 565 nm and 635 nm. In deaerated toluene, λ_{ex} = 330 nm, c = 1.0 × 10^{-3} M, 20 °C.

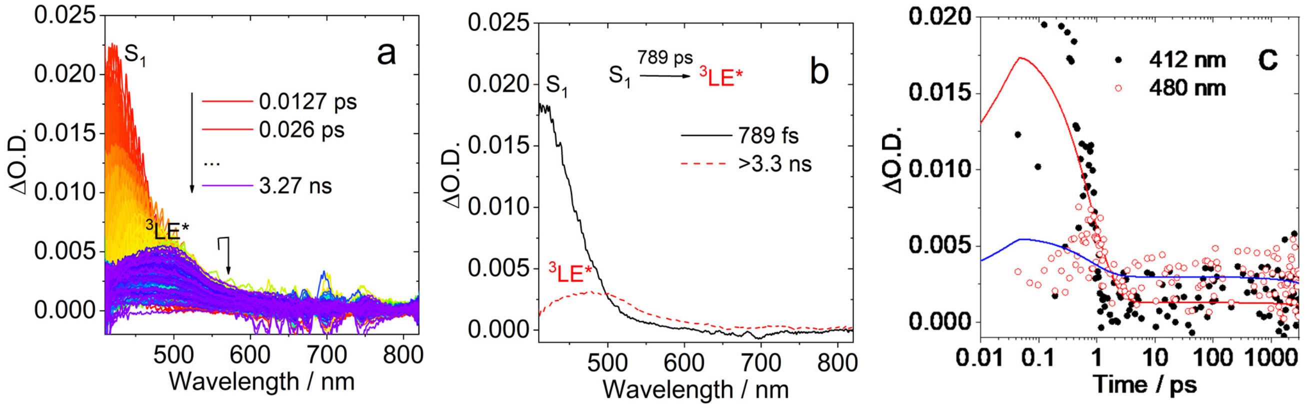

Figure S37. (a) Femtosecond transient absorption spectra of (a) **RB-S**, color code goes from red to blue covering the time interval from 1.3 fs to 3.3 ns. SADS of (b) **RB-S** obtained from global analysis. (c) Decay kinetics of **RB-S** at 412 nm and 480 nm. In deaerated toluene, λ_{ex} = 330 nm, c = 1.0 × 10^{-3} M, 20 °C.

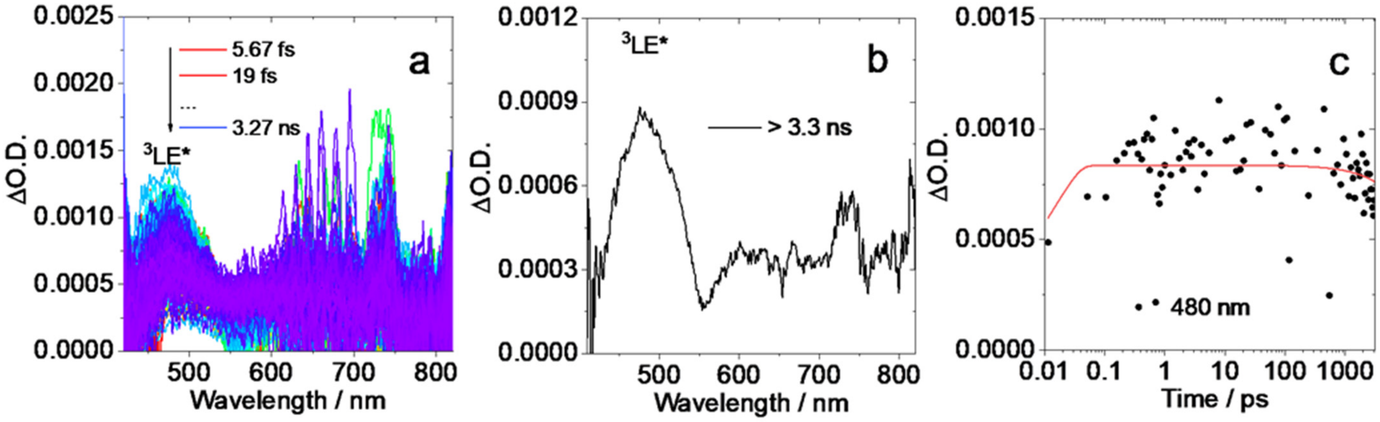

Figure S38. (a) Femtosecond transient absorption spectra of (a) **RB-C**, color code goes from red to blue covering the time interval from 5.7 fs to 3.3 ns. SADS of (b) **RB-C** obtained from global analysis. (c) Decay kinetics of **RB-C** at 480 nm. In deaerated hexane, λ_{ex} = 330 nm, *c* = 1.0 × 10^{-3} M, 20 °C.

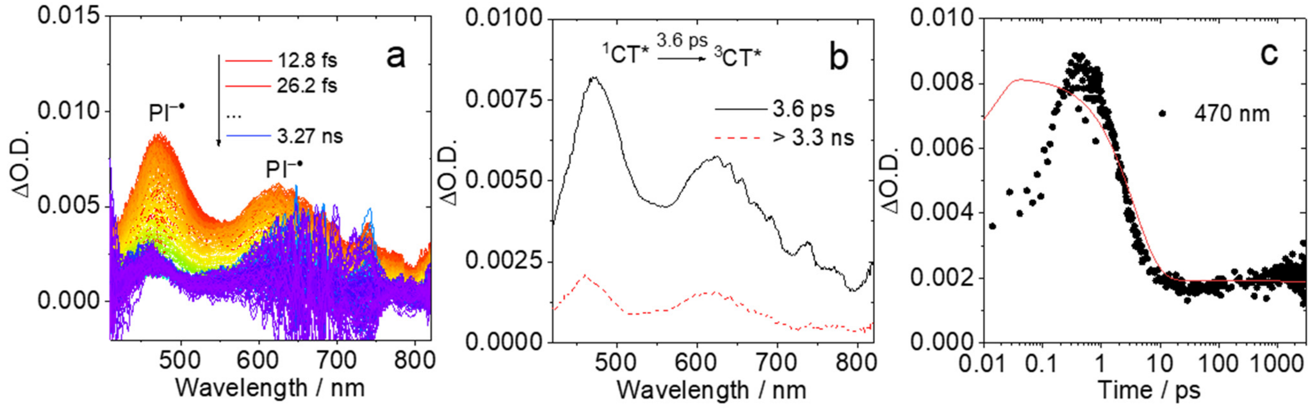

Figure S39. (a) Femtosecond transient absorption spectra of (a) **PI-Rho-S**, color code goes from red to blue covering the time interval from 12.8 fs to 3.3 ns. SADS of (b) **PI-Rho-S** obtained from global analysis. (c) Decay kinetics of **PI-Rho-S** at 470 nm. In deaerated acetonitrile, λ_{ex} = 330 nm, *c* = 1.0 × 10^{-3} M, 20 °C.

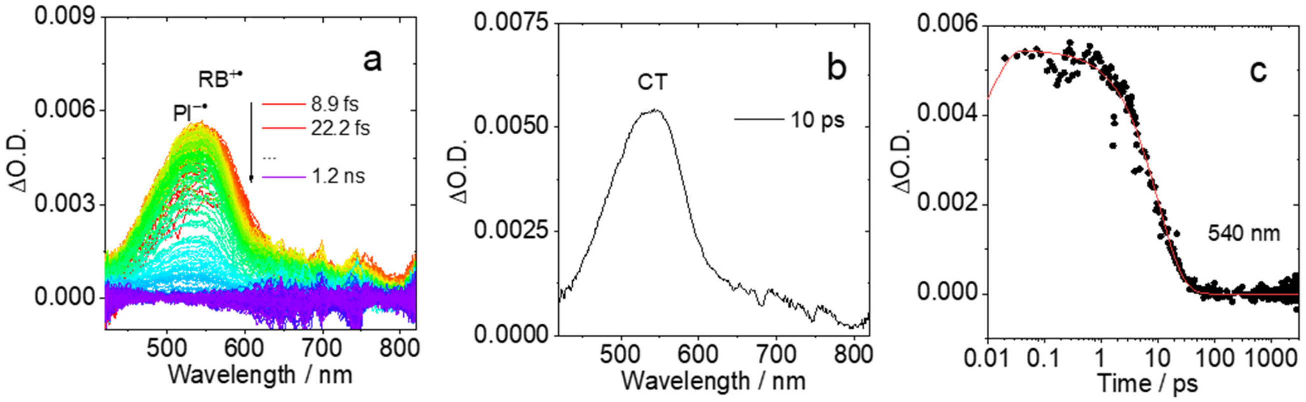

Figure S40. (a) Femtosecond transient absorption spectra of (a) **PI-Rho**, color code goes from red to blue covering the time interval from 8.9 fs to 1.2 ns. SADS of (b) **PI-Rho** obtained from global analysis. (c) Decay kinetics of **PI-Rho** at 540 nm. In deaerated acetonitrile, λ_{ex} = 330 nm, c = 1.0 × 10^{-3} M, 20 °C.

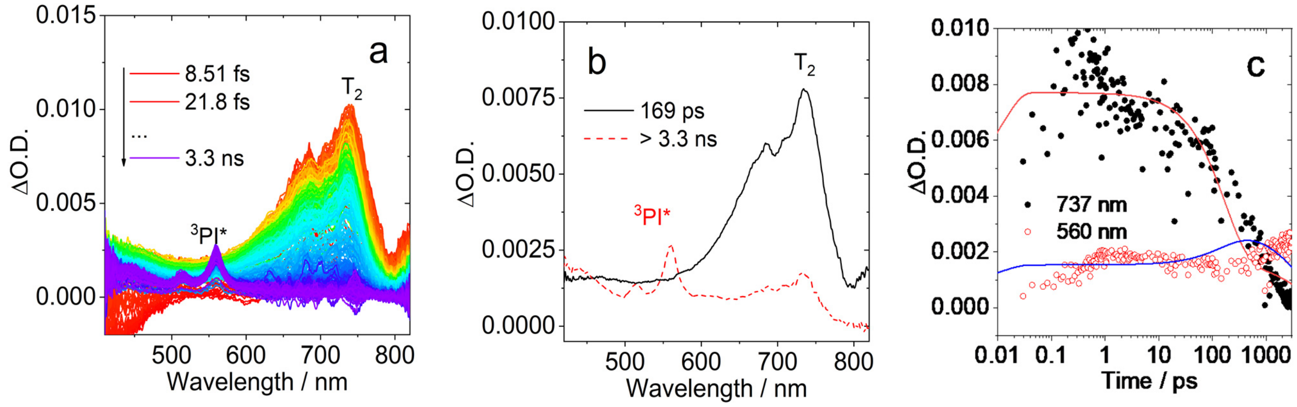

Figure S41. (a) Femtosecond transient absorption spectra of (a) **PI**, color code goes from red to blue covering the time interval from 8.5 fs to 3.3 ns. SADS of (b) **PI** obtained from global analysis. (c) Decay kinetics of **PI** at 737 nm and 560 nm. In deaerated acetonitrile, λ_{ex} = 330 nm, c = 1.0 × 10^{-3} M, 20 °C.

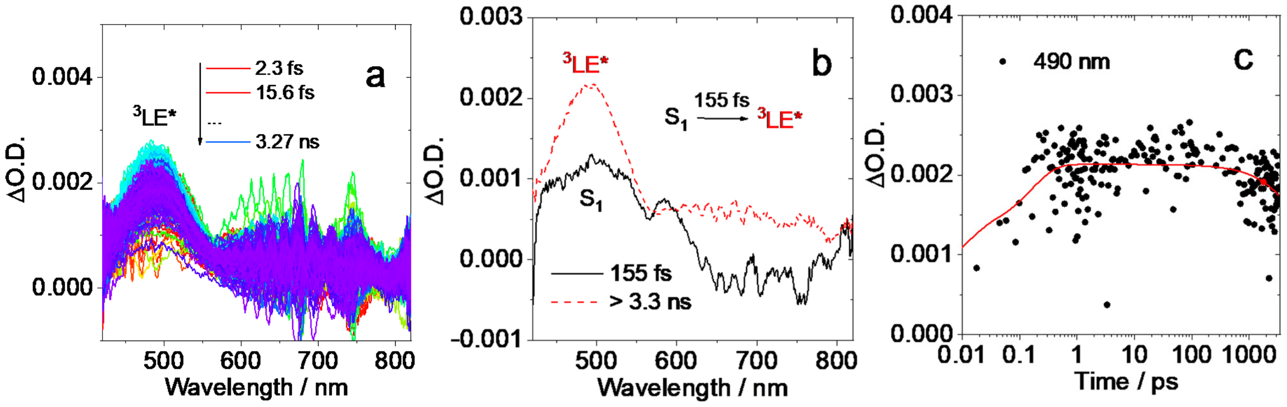

Figure S42. (a) Femtosecond transient absorption spectra of (a) **RB-S**, color code goes from red to blue covering the time interval from 2.3 fs to 3.3 ns. SADS of (b) **RB-S** obtained from global analysis. (c) Decay kinetics of **RB-S** at 490 nm. In deaerated acetonitrile, λ_{ex} = 330 nm, c = 1.0 × 10^{-3} M, 20 °C.

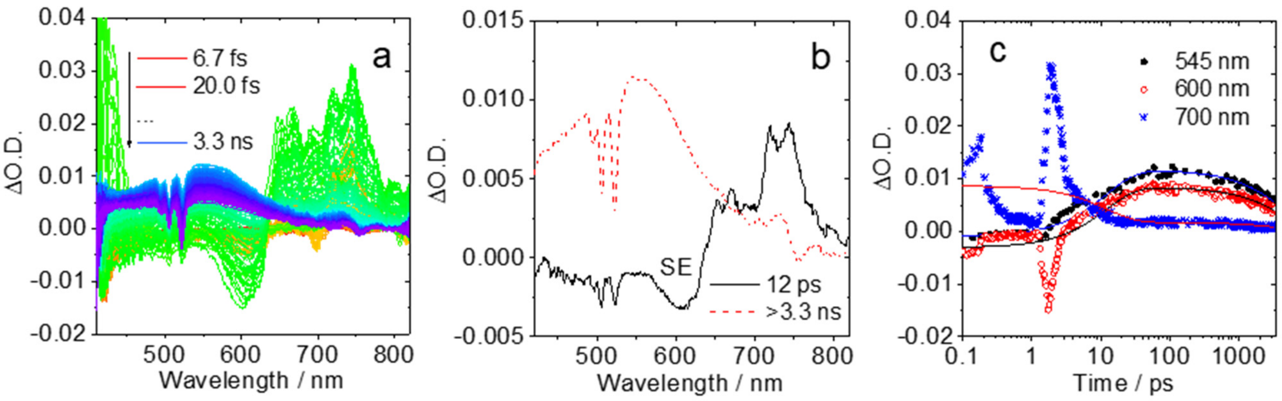

Figure S43. (a) Femtosecond transient absorption spectra of **PI-Rho**, color code goes from red to blue covering the time interval from 6.7 fs to 3.3 ns. (b) SADS of **PI-Rho** obtained from global analysis. (c) Decay kinetics of **PI-Rho** at 545 nm, 600 nm, 700 nm. In deaerated toluene, λ_{ex} = 370 nm, c = 1.0 × 10^{-3} M, 20 °C.

For the femtosecond transient absorption, excitation at the CT absorption band at 370 nm were repeated in Figure S43 and S44. We don't expect drastic change of the photophysics of the D-A compounds upon photoexcitation at different wavelength, and the conclusion of the manuscript will not be changed by excitation at different wavelength. When using 370 nm excitation of the compound, the first species at 600-800 nm was attributed to ^{1}CT state with incomplete charge separation that appears around 12 ps. Another long-lived species (>3.3 ns) centred 545 nm was attributed to ^{3}CT absorption.

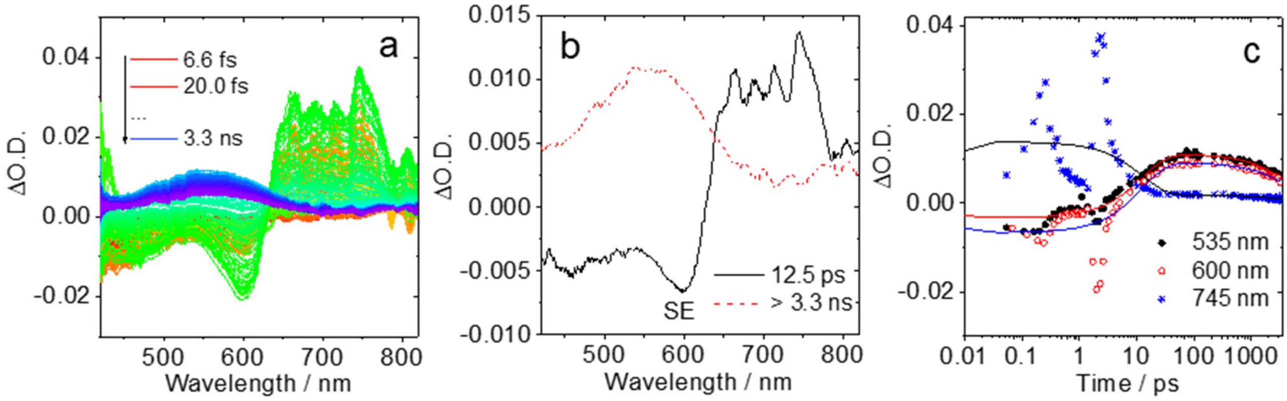

Figure S44. (a) Femtosecond transient absorption spectra of **PI-Rho-S**, color code goes from red to blue covering the time interval from 6.6 fs to 3.3 ns. (b) SADS of **PI-Rho-S** obtained from global analysis. (c) Decay kinetics of **PI-Rho-S** at 535 nm, 600 nm and 745 nm. In deaerated toluene, λ_{ex} = 370 nm, c = 1.0 × 10^{-3} M, 20 °C.

7. Absolute fluorescence quantum yields.

Table S3. Absolute fluorescence quantum yields (Φ_F) of the compounds in different solvents.[a]

Compounds	HEX (31.0)	TOL (33.9)	DCM (40.7)	ACN (45.6)	MEOH (55.4)
**PI-Rho**	0.230	0.103	0.025	0.006	0.004
**PI-Rho-S**	0.022	0.017	0.015	0.002	0.001
**RB-C**	0.085	0.022	0.131	0.032	0.007
**RB-S**	0.023	0.012	0.022	0.006	0.004
**PI**	0.026	0.013	0.034	0.011	0.003

[a] The values in the parenthesis are the E_T (30) values of the solvents, in kcal/mol.

8. Absorption of the radical anions obtained with chemical reduction

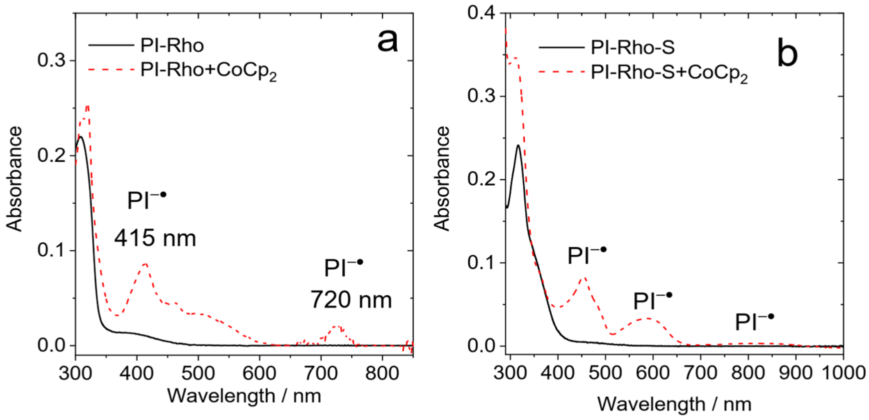

Figure S45. Chemical reduction absorption spectra of (a) **PI-Rho**, $c = 1.0 \times 10^{-5}$ M; (b) **PI-Rho-S**, $c = 5.0 \times 10^{-5}$ M in deaerated DMF, 20 °C.

9. Cyclic Voltammogram of the Compounds and Spectroelectrochemistry.

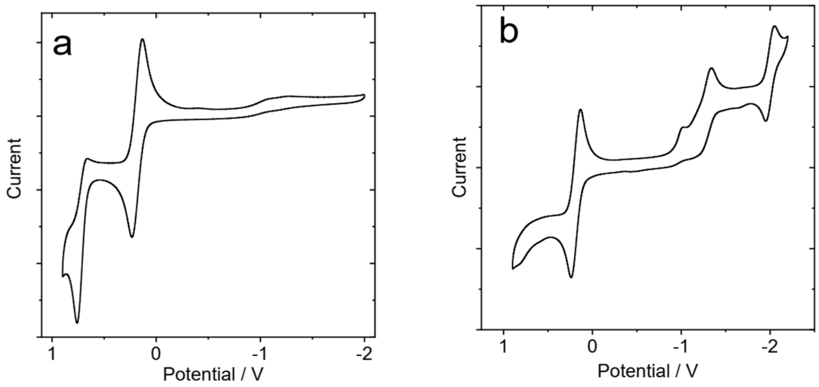

Figure S46. Cyclic voltammograms of (a) **RB-S**, (b) **PI-O-Rho** in deaerated dichloromethane and Ferrocene (Fc/Fc^+) was used as internal reference (set as 0 V in the cyclic voltammograms). Condition: in deaerated dichloromethane containing 0.10 mol/L $Bu_4N[PF_6]$ as supporting electrolyte, $Ag/AgNO_3$ as reference electrode. Scan rates: 50 mV/s. $c = 1.0 \times 10^{-5}$ M, 20 °C.

$$\Delta G_{\mathrm{CS}} = e(E_{\mathrm{OX}} - E_{\mathrm{RED}}) - E_{00} + \Delta G_{\mathrm{S}} \qquad (2)$$

$$\Delta G_{\mathrm{S}} = -\frac{e^2}{4\pi\varepsilon_{\mathrm{S}}\varepsilon_0 R_{\mathrm{CC}}} - \frac{e^2}{8\pi\varepsilon_0}\left(\frac{1}{R_{\mathrm{D}}} + \frac{1}{R_{\mathrm{A}}}\right)\left(\frac{1}{\varepsilon_{\mathrm{REF}}} - \frac{1}{\varepsilon_{\mathrm{S}}}\right) \qquad (3)$$

$$E_{\mathrm{CSS}} = e(E_{\mathrm{OX}} - E_{\mathrm{RED}}) + \Delta G_{\mathrm{S}} \qquad (4)$$

where ΔG_{S} is correction term for the effects of solvent polarity as well as the static Coulombic energy between donor and acceptor, *e* represents the electronic charge, ε_{S} is the static dielectric constant of the solvent, HEX (1.88), TOL (2.38), DCM (8.90) and ACN (37.5). ε_0 is the permittivity of free space, $\varepsilon_{\mathrm{REF}}$ is the static dielectric constant of the solvent used for electrochemical studies, DCM (8.90). R_{CC} is the center-to-center separation distance between the electron donor and electron acceptor, determined by the conformation optimized with DFT calculations, R_{D} is the radius of electron donor, R_{A} is the radius of electron acceptor; E_{OX} is the half-wave potential for one-electron reduction of the electron donor unit, E_{RED} is the half-wave potential for one-electron reduction of the electron acceptor unit, E_{00} is the energy approximated with the crossing point of the normalized UV–vis absorption spectra and fluorescence emission spectra. For **PI-Rho**, R_{D} =5.10 Å, R_{A} =4.83 Å, R_{CC} =8.13 Å, E_{00} = 3.88 eV, For **PI-Rho-S**, R_{D} =5.10 Å, R_{A} =4.83 Å, R_{CC} =8.13 Å, E_{00} = 3.84 eV. In *n*-hexane and toluene solution the Born equation does not give correct values for ΔG_{CS} because it overestimates the polarity of *n*-hexane and toluene. Thus in the Scheme 2 for the CT state energy levels in n-hexane and toluene were calculated based on CT absorption band and CT fluorescence spectra.

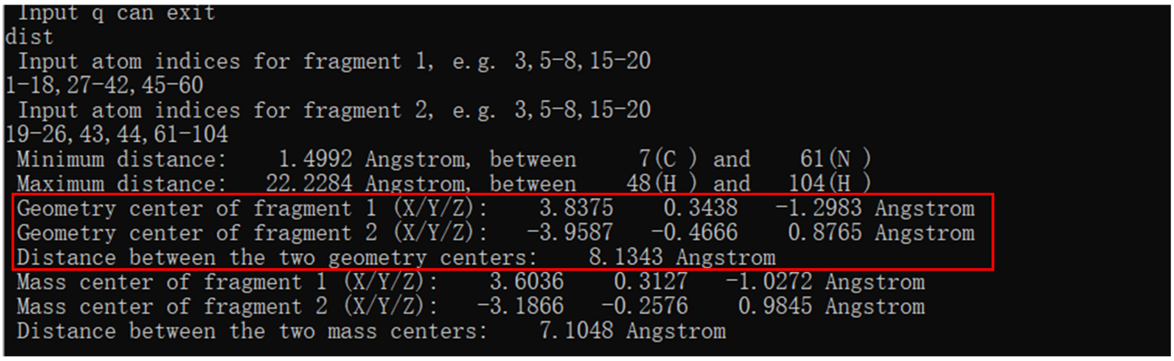

```
 Input q can exit
dist
 Input atom indices for fragment 1, e.g. 3,5-8,15-20
1-18,27-42,45-60
 Input atom indices for fragment 2, e.g. 3,5-8,15-20
19-26,43,44,61-104
 Minimum distance:    1.4992 Angstrom, between     7(C ) and    61(N )
 Maximum distance:   22.2284 Angstrom, between    48(H ) and   104(H )
 Geometry center of fragment 1 (X/Y/Z):    3.8375    0.3438   -1.2983 Angstrom
 Geometry center of fragment 2 (X/Y/Z):   -3.9587   -0.4666    0.8765 Angstrom
 Distance between the two geometry centers:    8.1343 Angstrom
 Mass center of fragment 1 (X/Y/Z):    3.6036    0.3127   -1.0272 Angstrom
 Mass center of fragment 2 (X/Y/Z):   -3.1866   -0.2576    0.9845 Angstrom
 Distance between the two mass centers:    7.1048 Angstrom
```

Figure S47. R_{CC} =8.13 Å for **PI-Rho** and **PI-Rho-S**, the value was obtained by multiwfn.

Table S4. Electrochemical Redox and Reduction Potentials [a]

	E_{OX} (V)	E_{RED} (V)
**PI**	–[b]	–1.37, –1.95
**RB-C**	+0.54, 0.73	– [b]
**RB-S**	+0.76	– [b]
**PI-O-Rho**	– [b]	–1.34, –2.00

[a] Cyclic voltammetry in N_2-saturated DCM containing a 0.10 M Bu_4NPF_6 supporting electrolyte; Pt electrode was used as the counter electrode; the working electrode is glassy carbon electrode; Ag/AgCl couple is the reference electrode. The value was obtained by setting the oxidation potential of Fc^+/Fc as 0. [b] Not observed.

10. DFT Calculations.

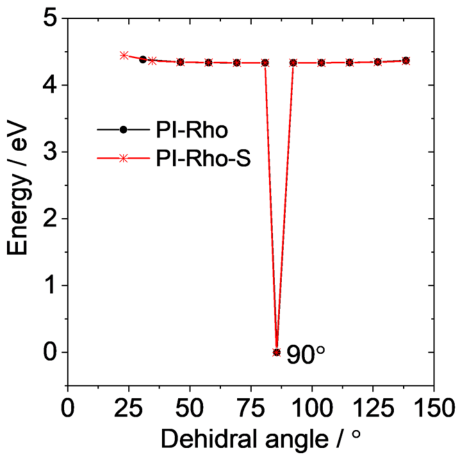

Figure S48. Singlet ground state (S_0) potential energy curves of **PI-Rho, PI-Rho-S**. Calculated by DFT at B3LYP/6-31G(d) level with Gaussian 09. Fix all other bond lengths and bond angles of the molecule, only rotate the dihedral angles of the spiro ring carbon atoms.

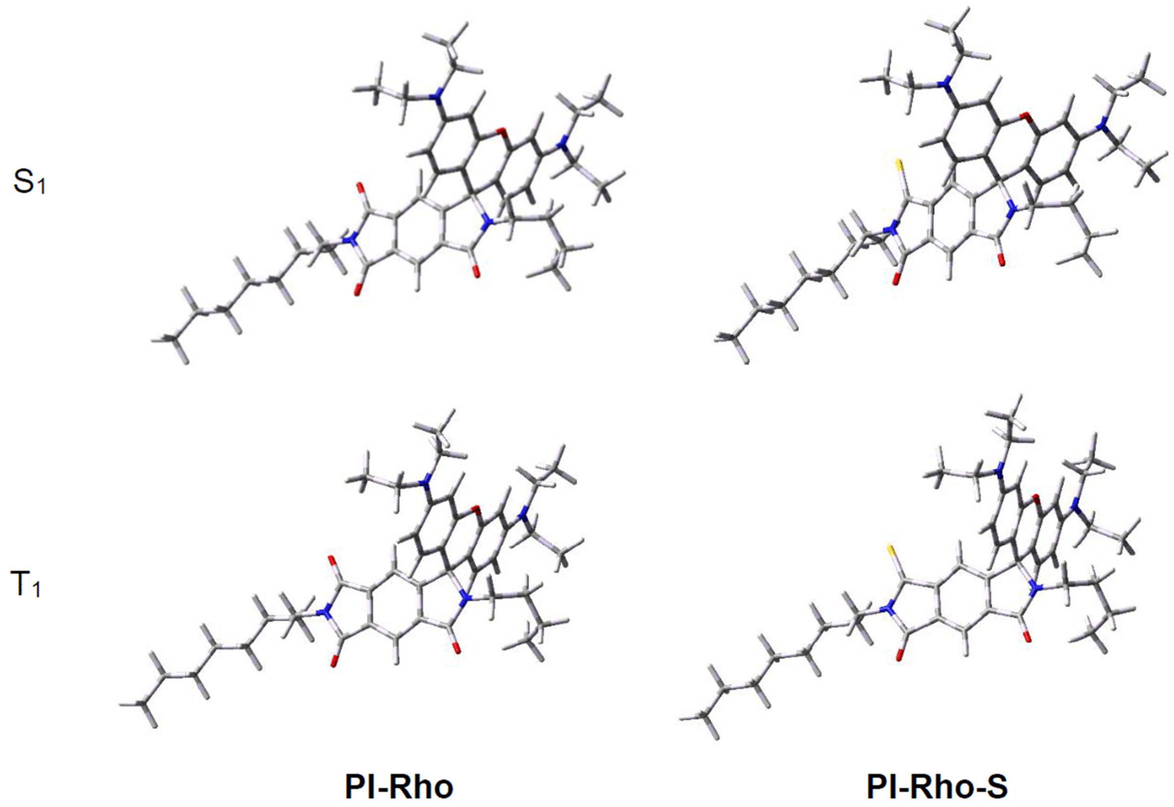

Figure S49. Singlet excited state (S_1) and triplet excited state (T_1) of **PI-Rho** and **PI-Rho-S**, calculated by DFT at B3LYP/6-31G(d) level with Gaussian 09.

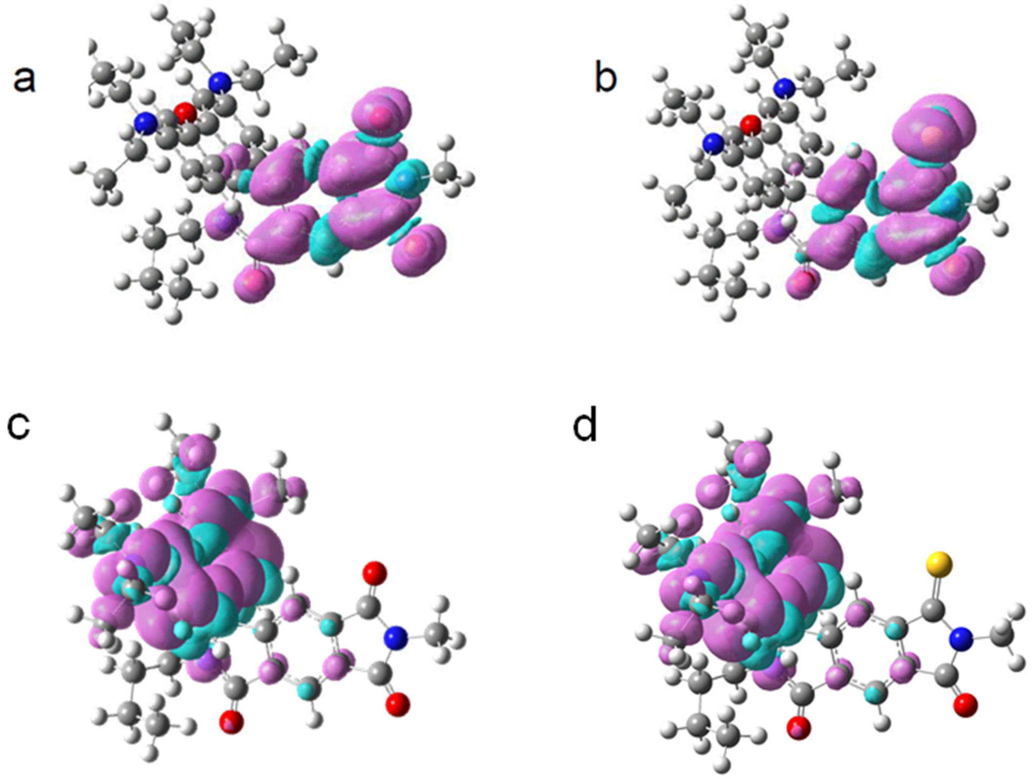

Figure S50. Triplet state spin density of (a) **PI-Rho** anion, (b) **PI-Rho-S** anion, (c) **PI-Rho** cation and (d) **PI-Rho-S** cation (isovalue = 0.0004 a.u.) in acetonitrile (CPCM model). Calculated by DFT at B3LYP/6-31G(d) level with Gaussian 09.

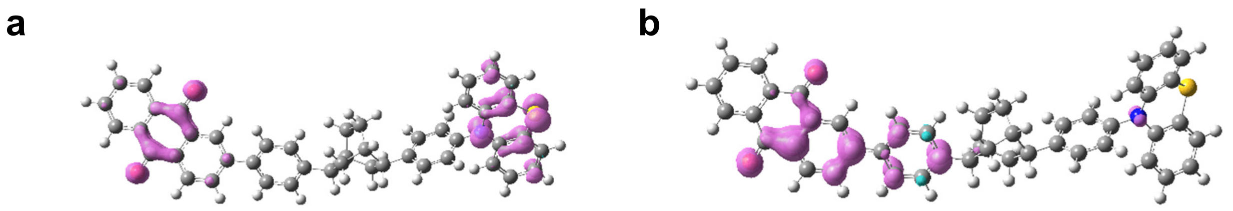

Figure S51. Triplet state spin density of **PTZ-AQ** (a) THF and (b) TOL (isovalue = 0.004 a.u.) (CPCM model). Calculated by DFT at B3LYP/6-31G(d) level with Gaussian 09.

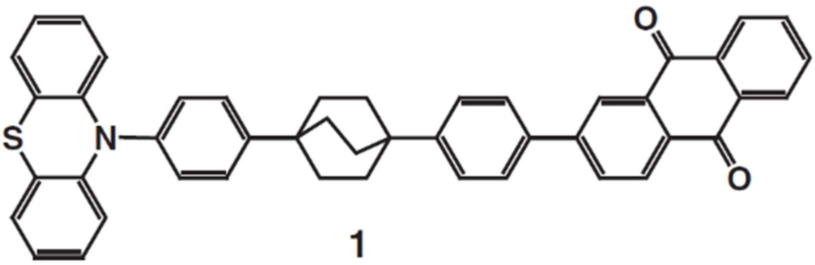

1

Figure S52. Molecular structure of **PTZ-AQ**, reported in Okada et al., *Chem. Lett.* **2013**, *42*, 794–796.

This compound was reported in Keiji Okada et al., *Chem. Lett.* **2013**, *42*, 794–796. Picosecond transient absorption spectra show that a CT state was observed in THF, but a ^{3}AQ state was observed in toluene. Herein we show that these states can be successfully predicted by DFT computations. These results show that it is convincing to use the DFT computation on the spin density of T_1 state predict the formation of either a ^{3}LE state or a ^{3}CT state.

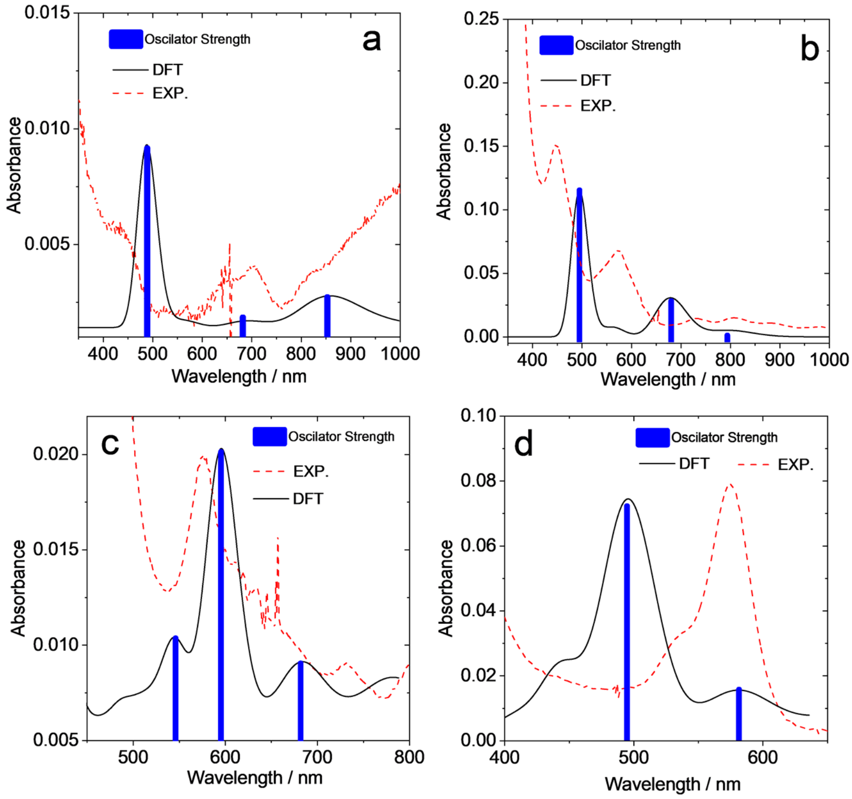

Figure S53. UV−vis absorption spectra of the compounds (a) **PI-Rho** anion, (b) **PI-Rho-S** anion, (c) **PI-Rho** cation, (d) **PI-Rho-S** cation calculated by DFT at B3LYP/6-31G(d) level with Gaussian 09.

Table S5. Triplet energy of the compounds **RB-C**, **PI**, **PIO** and **PIS** calculated by DFT at B3LYP/6-31G(d) level with Gaussian 09.

T_1 energy /eV	Calculate	Experiment
**RB-C**	3.42	3.36 [a]
**PI**	2.81	2.45
**PIO**	3.14	– [b]
**PIS**	2.16	– [b]

[a] Triplet state energy of **RB-C**, reported in *J. Org. Chem.* **2015**, *80*, 568−581; [b] Not studied.

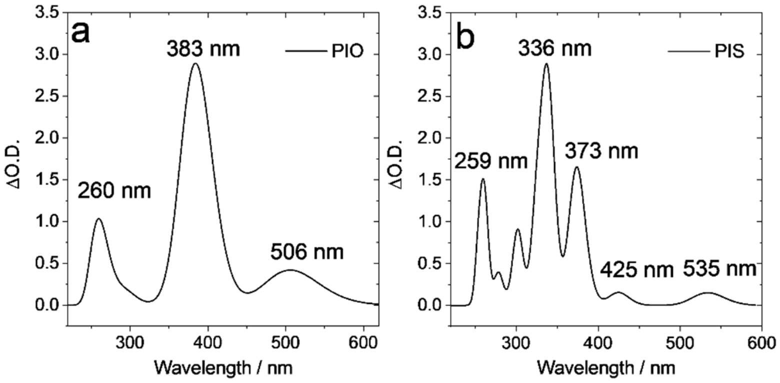

Figure S54. The calculated nanosecond transient absorption spectra of (a) **PIO**, (b) **PIS** in acetonitrile (CPCM model). Calculated by DFT at B3LYP/6-31G(d) level with Gaussian 09.

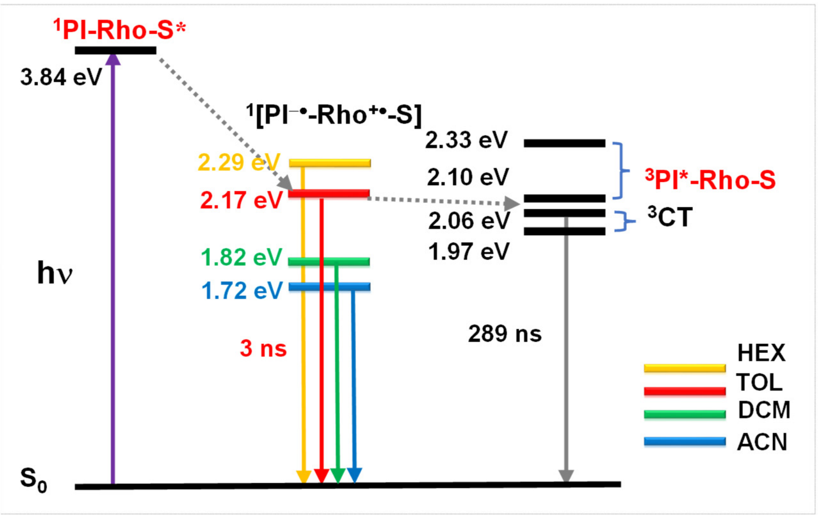

Scheme S2. Simplified Jablonski diagram illustrating the photophysical processes involved in **PI-Rho-S**.[1]CT energy levels were calculated based on the electrochemical data. TDDFT calculations were performed at the B3LYP/6-31G(d) level in toluene (CPCM model) by using Gaussian 09.

Table S6. Main Transition Orbitals, Electronic Excitation Energies (eV) and Corresponding Oscillator Strengths (*f*) of Singlet and triplet Excited States of **PI-Rho**. Calculation Was Performed at the B3LYP/6-31G(d) Level in vacuum with Gaussian 09. Base on the optimized ground state geometry.

	Electronic transition [a]	Energy [b]	*f* [c]	CI [d]	main transition	Composition [e]
Singlet	$S_0 \to S_1$	2.70 eV/459 nm	0.0154	0.6958	H→L	CT
	$S_0 \to S_2$	2.75 eV/450 nm	0.0055	0.6929	H−1→L	CT
	$S_0 \to S_3$	3.32 eV/374 nm	0.0055	0.7031	H−2→L	CT
	$S_0 \to S_4$	3.60 eV/344 nm	0.0138	0.6870	H→L+1	CT
	$S_0 \to S_5$	3.65 eV/340 nm	0.0024	0.6885	H−1→L+1	CT
Triplet [f]	$S_0 \to T_1$	2.61 eV/474 nm	0.0000	0.6793	H→L	CT
	$S_0 \to T_2$	2.72 eV/455 nm	0.0000	0.6987	H−1→L	CT
	$S_0 \to T_3$	3.17 eV/391 nm	0.0000	0.3358	H−6→L	CT
	$S_0 \to T_4$	3.30 eV/376 nm	0.0000	0.6830	H−2→L	CT
	$S_0 \to T_5$	3.35 eV/370 nm	0.0000	0.6577	H−7→L	LE

[a] TDDFT//B3LYP/6-31G(d), based on the DFT//B3LYP/6-31G(d)-optimized ground state geometries. [b] Only the selected low-lying excited states are presented. [c] Oscillator strengths. [d] CI coefficients are in absolute values. [e] TDDFT//B3LYP/6-31G(d)-optimized excited state geometries. [f] No spin−orbital coupling effect was considered; thus, the *f* values are zero.

Table S7. Main Transition Orbitals, Electronic Excitation Energies (eV) and Corresponding Oscillator Strengths (*f*) of Singlet and triplet Excited States of **PI-Rho-S**. Calculation Was Performed at the B3LYP/6-31G(d) Level in toluene (CPCM model) with Gaussian 09. Base on the optimized ground state geometry.

	Electronic transition [a]	Energy [b]	*f* [c]	CI [d]	main transition [e]	Composition
Singlet	$S_0 \rightarrow S_1$	2.01 eV/616 nm	0.0165	0.7045	H→L	CT
	$S_0 \rightarrow S_2$	2.08 eV/596 nm	0.0048	0.7025	H–1→L	CT
	$S_0 \rightarrow S_3$	2.42 eV/513 nm	0.0001	0.7022	H–3→L	LE
	$S_0 \rightarrow S_4$	2.66 eV/466 nm	0.0035	0.7046	H–2→L	CT
	$S_0 \rightarrow S_5$	3.33 eV/384 nm	0.0320	0.6790	H–5→L	LE
Triplet [f]	$S_0 \rightarrow T_1$	1.97 eV/630 nm	0.0000	0.6998	H→L	CT
	$S_0 \rightarrow T_2$	2.06 eV/601 nm	0.0000	0.6809	H–1→L	CT
	$S_0 \rightarrow T_3$	2.10 eV/590 nm	0.0000	0.6756	H–3→L	LE
	$S_0 \rightarrow T_4$	2.33 eV/532 nm	0.0000	0.6322	H–5→L	LE
	$S_0 \rightarrow T_5$	2.66 eV/467 nm	0.0000	0.7041	H–2→L	CT

[a] TDDFT//B3LYP/6-31G(d), based on the DFT//B3LYP/6-31G(d)-optimized ground state geometries. [b] Only the selected low-lying excited states are presented. [c] Oscillator strengths. [d] CI coefficients are in absolute values. [e] TDDFT//B3LYP/6-31G(d)-optimized excited state geometries. [f] No spin−orbital coupling effect was considered; thus, the *f* values are zero.

References

[1] R. T. Hayes, C. J. Walsh, and M. R. Wasielewski, Competitive electron transfer from the S_2 and S_1 excited states of zinc meso-tetraphenylporphyrin to a covalently bound pyromellitimide: dependence on donor−acceptor structure and solvent, *J. Phys. Chem. A*, **2004**, *108*, 2375–2381.

[2] K. H. Cruickshank and M. L. Bittner, Green fluorescent labeled nucleotides for use in probes. WO1994006812 A1. 1994.

[3] A. Karimata, H. Kawauchi, S. Suzuki, M. Kozaki, N. Ikeda, K. Keyaki, K. Nozaki, K. Akiyama, and K. Okada, Photoinduced charge separation of 10-phenyl-10h-phenothiazine–2-phenylanthraquinone dyad bridged by bicyclo[2.2.2]octane, *Chem. Lett.*, **2013**, *42*, 794–796.

[4] J Herbich, A Kapturkiewicz. Electronic and molecular structure of charge transfer singlet states: 4-(9 anthryl)-julolidine and 4-(9-acridyl)julolidine, *Chem. Phys. Lett.* **1997**, *273*, 8.

[5] Hicks, F. A., Kablaoui, N. M., Buchwald, S. L. Titanocene-catalyzed cyclocarbonylation of enynes to cyclopentenones. *J. Am. Chem. Soc.*, **1996**, *118*, 9450–9451.

[6] S. Stoll, A. Schweiger, *J. Magn. Reson.* **2006**, *178*, 42–55.

[7] A. Barbon, M. Bortolus, A. L. Maniero, M. Brustolon, *Phys. Chem. Chem. Phys.* **2005**, *7*, 2894–2899.
